# Advancing bioinformatics with language models: components, applications, and perspectives

**DOI:** 10.1093/bib/bbag367

**Published:** 2026-07-10

**Authors:** Jiajia Liu, Mengyuan Yang, Yankai Yu, Haixia Xu, Tiangang Wang, Kang Li, Xiaobo Zhou

**Affiliations:** Center for Computational Systems Medicine, McWilliams School of Biomedical Informatics, The University of Texas Health Science Center at Houston, 7000 Fannin St, Houston, Houston, TX 77030, United States; Department of Cell Biology and Genetics, School of Basic Medical Sciences, Xi’an Jiaotong University Health Science Center, No. 28 Xianning West Road, Xian City, Shaanxi Province, 710049, P.R. China; School of Computing and Artificial Intelligence, Southwest Jiaotong University, No. 999, Xi'an Road, Pidu District, Chengdu, Sichuan, 611756, P.R. China; Center for Computational Systems Medicine, McWilliams School of Biomedical Informatics, The University of Texas Health Science Center at Houston, 7000 Fannin St, Houston, Houston, TX 77030, United States; Center for Computational Systems Medicine, McWilliams School of Biomedical Informatics, The University of Texas Health Science Center at Houston, 7000 Fannin St, Houston, Houston, TX 77030, United States; West China Biomedical Big Data Center, West China Hospital, Sichuan University, No. 17, Section 3, South Renmin Road, Chengdu, Sichuan, 610041, P.R. China; Center for Computational Systems Medicine, McWilliams School of Biomedical Informatics, The University of Texas Health Science Center at Houston, 7000 Fannin St, Houston, Houston, TX 77030, United States; McGovern Medical School, The University of Texas Health Science Center at Houston, 6431 Fannin St, Houston, Houston, TX 77030, United States; School of Dentistry, The University of Texas Health Science Center at Houston, 7500 Cambridge St, Houston, TX 77030, United States

**Keywords:** language model, foundation model, transformer architecture, multi-omics application, drug discovery, single-cell analysis

## Abstract

Large language models (LLMs) are deep learning-based artificial intelligence models that have achieved remarkable success in natural language processing. Typically composed of neural networks with billions of parameters, they are trained on massive unlabeled datasets using self-supervised or semi-supervised learning. Beyond language, LLMs hold immense potential for addressing complex bioinformatics challenges. This review provides a comprehensive overview of transformer-based model applications in genomics, transcriptomics, proteomics, drug discovery, and single-cell analysis. We discuss critical components, including tokenization strategies for diverse biological data, transformer architectures, attention mechanisms, and pretraining approaches. We also survey currently available foundation models and their downstream applications across bioinformatics domains. Finally, we highlight major challenges that remain insufficiently addressed in prior reviews and outline future perspectives and design principles for next-generation biological language models, offering practical guidance for both users and developers.

## Introduction

The rapid expansion of biological data, driven by high-throughput sequencing, single-cell multi-omics, proteomics, and spatial transcriptomics, has created unprecedented opportunities and challenges for bioinformatics. Traditional computational methods often struggle to capture the complex, context-dependent relationships inherent in these data. Large language models (LLMs), originally developed for natural language processing, offer a transformative solution by learning rich, high-dimensional representations from sequences and structured biological data. Leveraging transformer architectures, LLMs can model dependencies across genes, proteins, and other biomolecules, enabling tasks ranging from molecular property prediction to single-cell trajectory inference [1, 2]. Beyond sequence modeling, LLMs facilitate integrative analyses across modalities, capturing interactions between DNA, RNA, and proteins.

**Figure 1:**
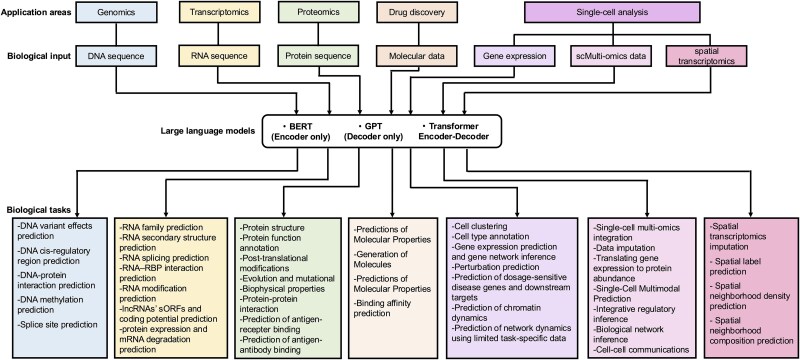
Summary of the application of language models in bioinformatics in this review. Applications of language models (LMs) in bioinformatics include applications in genomics, transcriptomics, proteomics, drug discovery, and single-cell analysis. Applications of LMs in genomics focus on LMs using DNA sequence; applications of LMs in transcriptomics focus on LMs using RNA sequence; applications of LMs in proteomics focus on LMs using protein sequence; applications of LMs in drug discovery focus on LMs using molecular data; and applications of LMs in single-cell analysis focus on LMs using scRNA-seq, scMulti-omics, and spatial transcriptomics data. Each corresponds to a variety of biological downstream tasks.

In this review, we provided a comprehensive overview of the essential components of language models in bioinformatics, spanning genomics, transcriptomics, proteomics, drug discovery, and single-cell analysis, including large foundation models and task-specific transformer-based models. To avoid potential misunderstanding, we clarify that in the context of bioinformatics, the term large language models specifically refers to foundation models with billions of parameters that require extensive pretraining and fine-tuning, whereas language models more broadly encompass both these large foundation models and smaller, task-specific transformer architectures. Key aspects covered include tokenization methods for diverse data types, transformer architecture, attention mechanism, and pretraining processes. Additionally, we will introduce currently available foundation models and highlight their downstream applications across various bioinformatics domains (Fig. 1). Finally, we will highlight major challenges that remain insufficiently addressed in prior reviews and outline future perspectives and design principles for next-generation biological language models, offering practical guidance for both users and developers.

## Understanding the building blocks of language models in bioinformatics

To establish a foundation for subsequent applications, we first examine the core technical components that underpin language models in bioinformatics.

### Tokenization and input embedding

**Figure 2:**
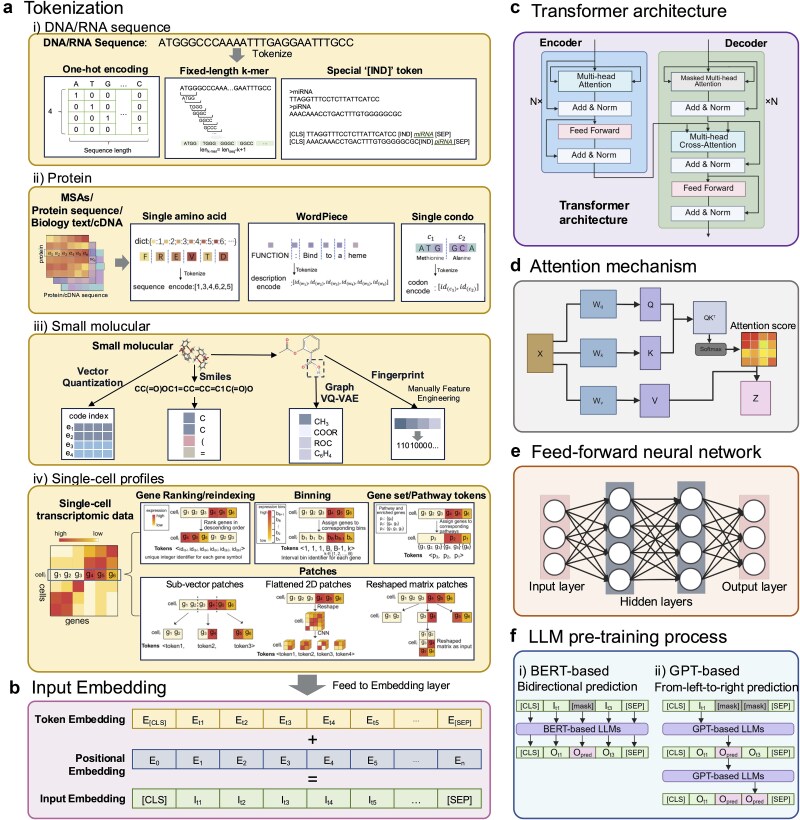
Building blocks of large language models in bioinformatics. (a) Tokenization methods tailored to various data types, including DNA/RNA sequences, proteins, small molecules, and single-cell data. (b) Input embedding strategies used in large language models to encode tokenized data. (c) Schematic representation of the transformer architecture, a foundational structure in LLMs. (d) The attention mechanism, enabling models to focus on important features in sequences. (e) The feed-forward network, a critical component of transformers for learning hierarchical representations. (f) Pretraining processes for BERT and GPT-based models, highlighting BERT’s bidirectional prediction approach and GPT’s left-to-right prediction strategy.

Tokenization methods are essential for processing raw input data, breaking it down into smaller, manageable units (tokens) that can be analyzed and processed by models. The choice of tokenization method varies depending on the type of data (Fig. 2a, Table 1).

**Table 1:** Tokenization methods for different types of data.

**Application area**	**Data type**	**Method**	**Example**
Genomics/Transcriptomics	DNA/RNA sequence	Single-nucleotide tokenization	RNA-FM, RNA-MSM, ERNIE-RNA, RiNALMo
		Fixed-length k-mers	DNABERT, Nucleotide Transformer, DNABERT-2, DNAGPT, RNABERT, SpliceBERT, CodonBERT, UTR-LM
		Special ‘[IND]’ token	RNAErnie
Proteomics	MSAs/Protein sequences	Single amino acid tokenization	MSA Transformer/TAPE, ESM-1b, ProtTrans, Progen
	Biomedical text	WordPiece	ProtST
	cDNA	Single codon tokenization	CaLM
Drug discovery	Simplified Molecular-Input Line-Entry system (SMILES)	Vector Quantization	UniMoT
		SmilesTokenizer	ChemBERTa, ChemBERTa-2, MolGPT, DrugCLIP, DrugGPT, DrugReAlign
		Graph VQ-VAE	Mole-BERT
		fingerprint	SMILES-BERT
Single-cell analysis	Expression profiles	Gene expression Ranking	Geneformer, tGPT, iSEEEK
		Binning	scBERT, scGPT, scFormer, CellLM, BioFormers, CancerFoundation
		Gene set/Pathway tokens	TOSICA
		Patches	CIForm, scTranSort, scCLIP
		Gene value projection	scTranslator, scFoundation, scMulan, scGREAT
		Cell tokens	CellPLM, ScRAT, mcBERT

In DNA and RNA sequence analysis, tokenization converts raw nucleotide sequences (A, T, C, G for DNA or A, U, C, G for RNA) into numerical representations suitable for computational models. One-hot encoding, where each nucleotide is represented as a binary vector e.g. [1, 0, 0, 0] for A in DNA, is used in models like RNA-FM [3] and RNA-MSM [4]. K-mer tokenization, which segments sequences into overlapping substrings of fixed length “k” (e.g. for k = 3, “ATGC” becomes “ATG” and “TGC”), is widely applied in DNABERT [5], DNAGPT [6], and RNABERT [7]. Specialized tokens, such as “[IND],” can mark sequence boundaries or unknown characters, as implemented in RNAErnie [8].

Protein language models primarily use amino acid sequences, multiple sequence alignments (MSAs), biomedical text, and cDNA. Single-amino-acid tokenization is common, analogous to k-mer segmentation, as seen in ESM-1b [9], ProtTrans [10], and ProGen [11]. Biomedical and biological texts, including general descriptions, conditioning tags in generative models, and resources like Gene Ontology (GO), leverage NLP-inspired methods, such as WordPiece tokenization in ProtST [12], while cDNA sequences are tokenized into codons in models like CaLM [13].

In drug discovery, tokenization methods capture molecular structure and properties. SMILES tokenization segments molecules into atoms, bonds, and ring markers (MolGPT [13]). In contrast, UniMoT [14] adopts a learned vector-quantized tokenizer that produces high-level molecule tokens aligned with LLMs. Graph-based VQ-VAE encodes atoms into chemically meaningful subclasses, whereas fingerprint tokens, as in SMILES-BERT [14], summarize molecular patterns or properties into numerical vectors.

Single-cell data employs diverse tokenization strategies. Gene ranking/reindexing orders genes by expression, as in Geneformer [15] and tGPT [16]. Binning expression to discrete values is used in scBERT [17] and scGPT [18], while gene set/pathway-based methods group genes into biologically meaningful sets, exemplified by TOSICA [19]. Patch-based methods segment gene vectors into sub-vectors such as CIForm [20] or 2D patches used in scTranSort [21] and scCLIP [22]. Direct projection of gene expression is used in scFoundation [23] and scMulan [24]. Some approaches tokenize cells instead of genes, as in CellPLM [25], ScRAT [26], and mcBERT [27] (Table 1).

After tokenization, embedding converts discrete tokens into continuous vector representations, capturing semantic relationships. Positional encoding adds information about the order of tokens, either relatively or absolutely. The final input embedding combines token and positional embeddings, forming a unified representation for downstream model processing (Fig. 2b).

### Transformer architecture

Building on these representations, transformer architectures provide the computational framework for modeling complex dependencies within biological sequences and features. A transformer is the core architecture of language models, consisting of an encoder and a decoder. The encoder processes input sequences in parallel to capture relationships, while the decoder generates outputs from the processed information. Both use stacked layers of multi-head attention, add-and-norm, and feed-forward networks (Fig. 2c).

#### Attention mechanism

The attention mechanism, particularly self-attention [28], enables the model to assign relative importance to different token pairs in a sequence. In self-attention, each token computes a score based on how much attention it should pay to other tokens in the sequence. This is done by calculating three key components: Query (Q), Key (K), and Value (V) vectors for each token (Fig. 2d). The attention score is computed as the dot product between the Query of one token and the Key of another, followed by a softmax normalization. These scores are then used to weight the Value vectors, which are aggregated to form the output representation for each token as follows [28]:


(1) \begin{align*} Attention\left(Q,K,V\right)= softmax\left(\frac{Q{K}^T}{\sqrt{d_k}}\right)V \end{align*}

#### Multi-head attention

Extends this idea by running multiple attention mechanisms (or “heads”) in parallel. Each attention head processes the input tokens in a slightly different way by using different sets of learned weights for the Q, K, and V vectors. The results of all heads are concatenated and linearly transformed, allowing the model to capture different aspects of relationships between tokens simultaneously.

#### Add and norm layer

The add and norm layer performs layer normalization and residual connections, helping stabilize training by ensuring that the output of each layer is added to the input before normalization.

#### Feed-forward layer

After the attention mechanism, the feed-forward network is a fully connected neural network (Fig. 2e), helping the model learn complex mappings and capture more abstract representations of the input data.

#### Transformer encoder

Transformer encoders generate context-aware token representations using self-attention to capture dependencies across the input sequence (Supplementary Fig. 1). BERT [29] is a key example that uses bidirectional training to capture context from both sides of each token (Fig. 2f). Pretrained via a masked language model (MLM) task, it predicts randomly masked tokens, enabling richer, context-aware representations. Adaptations like scBERT apply BERT to single-cell RNA-seq data, masking gene tokens to learn dependencies and co-expression patterns.

#### Transformer decoder

Transformer decoders generate sequences autoregressively, predicting each token from previous ones. GPT [30] is a representative decoder that processes sequences unidirectionally, predicting each token from left to right using autoregressive learning (Fig. 2f). This makes it well-suited for generative tasks and zero-shot learning, performing tasks without task-specific training.

#### Transformer encoder-decoder

Encoder-decoder models combine contextual encoding and generative decoding, using cross-attention to link encoder outputs to decoder inputs. In this review, we categorize models into three groups: BERT-based encoders, GPT-based decoders, and transformer-based models (Supplementary Tables 1–4), where the latter specifically refers to encoder-decoder architectures.

## Foundation models in bioinformatics

**Figure 3:**
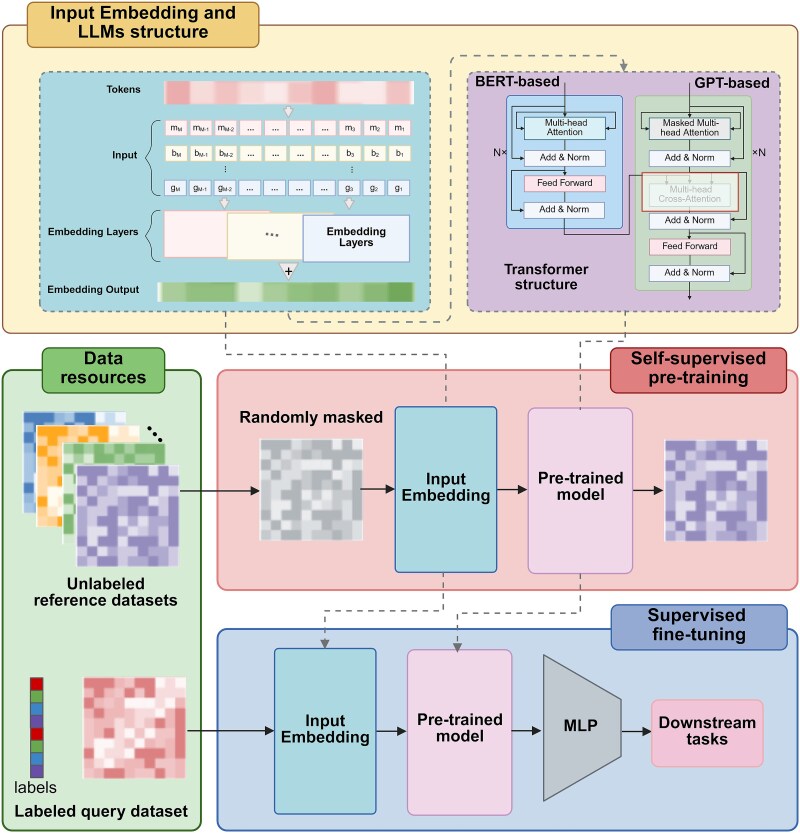
Schematic diagram of the large language model pretraining and fine-tuning process. The workflow begins with tokenizing the input data, which is then fed into the embedding layer and language models. The training process comprises two stages: pretraining and fine-tuning. Pretraining employs self-supervised learning on large-scale, unlabeled reference datasets to develop a general-purpose model with robust generalization capabilities. Fine-tuning builds upon the pretrained model, involving task-specific training to optimize performance for designated applications.

Having outlined the fundamental components, we next consider how these elements are integrated into large-scale foundation models with broad applicability across biological domains. Foundation models are large-scale, pretrained models designed to be versatile and adaptable across a wide range of downstream tasks [31]. They are trained on extensive and diverse datasets to capture broad, generalizable patterns. Their architectures, often transformer-based (e.g. GPT, BERT), are designed for flexibility and scalability, supporting a variety of input types and tasks. Self-supervised learning is central to their pretraining, using tasks such as masked prediction, next-token prediction, or contrastive learning to learn representations without labeled data. Foundation models exhibit multi-task transferability through pretraining on large datasets to learn general patterns, followed by fine-tuning on task-specific data to adapt to particular applications (Fig. 3). Finally, these models require substantial computational resources, often employing GPU or TPU clusters, and typically consist of billions to trillions of parameters.

### DNA foundation models

Currently, DNA sequence-based foundation models are powerful tools that leverage advanced transformer architectures to analyze and interpret genomic data [32] for the specific challenges of genomics (Table 2). For example, DNABERT [5] is a BERT-based model trained on the human reference genome, which captures nucleotide context for tasks like sequence classification and variant prediction. DNABERT-2 [33], trained on 135 species, enables cross-species analysis, while GROVER [34] focuses on functional genomics and gene expression in humans. DNAGPT [6], based on the GPT architecture, incorporates genomes from 10 species to facilitate sequence generation and evolutionary studies.

**Table 2:** Foundation models in bioinformatics.

**Application area**	**Model**	**Architecture**	**Pretraining data**	**Code available**
Genomics	GPN	Transformer-based	Reference genomes from 8 species	https://github.com/songlab-cal/gpn
	Nucleotide Transformer	Transformer-based	3.2 billion nucleotides in GRCh38/hg38 reference assembly, 20.5 trillion nucleotides including 125 million mutations (111 million SNPs, 14 million indels), and 174 billion nucleotides from 850 species	https://github.com/instadeepai/nucleotide-transformer
	DNABERT	BERT-based	2.75 billion nucleotide based human genome dataset	https://github.com/jerryji1993/DNABERT
	DNABERT-2	BERT-based	2.75 billion nucleotide based human genome dataset and 32.49 billion nucleotide bases from 135 species, spread across 6 categories	https://github.com/MAGICS-LAB/DNABERT_2
	MoDNA	BERT-based	Same as Nucleotide Transformer	https://github.com/uta-smile/MoDNA
	GROVER	BERT-based	*Homo sapiens* (human) genome assembly GRCh37 (hg19)	https://github.com/rowanz/grover
	MuLan-Methyl	BERT-based	3 main types of DNA methylation sites (6mA, 4mC, and 5hmC) across 12 genomes, in total 250 599 positive samples	https://github.com/husonlab/mulan-methyl
	iDNA-ABF	BERT-based	Same as MuLan-Methyl	https://github.com/FakeEnd/iDNA_ABF
	iDNA-ABT	BERT-based	Same as MuLan-Methyl	https://github.com/YUYING07/iDNA_ABT
	DNAGPT	GPT-based	Reference genomes from the Ensembl database include 3 billion bps, with a total of 1 594 129 992 bps across 9 species	https://github.com/TencentAILabHealthcare/DNAGPT
	GENA-LM	BERT-based	Human T2T v2 genome with gnomAD v3.1.2 variant augmentation; multispecies genomes from Ensembl release 106, including taxon-specific datasets	https://github.com/AIRI-Institute/GENA_LM
	Evo/Evo2	GPT-based	Reference genomes from multiple species, with Evo2 supporting pretraining on ultra-long genomic sequences of up to ~131 kb.	https://github.com/evo-design/evo https://github.com/ArcInstitute/evo2
Transcriptomics	RNABERT	BERT-based	76 237 human-derived small ncRNAs from RNAcentral	https://github.com/mana438/RNABERT
	RNA-FM	BERT-based	About 27 million ncRNA sequences across 47 different databases	https://github.com/ml4bio/RNA-FM
	RNA-MSM	BERT-based	4069 RNA families from rfam	https://github.com/yikunpku/RNA-MSM
	SpliceBERT	BERT-based	2 million sequences and approximately covering 65 billion nucleotides of 72 vertebrates from UCSC genome browser	https://github.com/biomed-AI/SpliceBERT
	UNI-RNA	BERT-based	23 million ncRNA sequences obtained from the RNAcentral database	https://github.com/ComDec/unirna-tools
	3UTRBERT	BERT-based	108 573 unique mRNA transcripts from the GENCODE and each contains 3754 nucleotides (median 3048 nts) on average.	https://github.com/yangyn533/3UTRBERT
	UTR-LM	BERT-based	214 349 unlabeled 5′ UTR sequences from Ensembl across 5 species	https://github.com/a96123155/UTR-LM
	RNAErnie	Transformer-based	23 million ncRNA sequences obtained from the RNAcentral database	https://github.com/CatIIIIIIII/RNAErnie
	HydraRNA	BERT-based	28.1 million protein-coding and noncoding RNAs from RNAcentral and NCBI RefSeq, covering ~51 billion nucleotide tokens across >1200 species	https://github.com/GuipengLi/HydraRNA
	RiNALMo	BERT-based	RNA sequences from ~4000 Rfam RNA families, covering tens of billions of nucleotides across diverse species.	https://github.com/lbcb-sci/RiNALMo
	ERNIE-RNA	BERT-based	~20 million noncoding RNA sequences obtained from RNAcentral and curated public RNA databases across multiple species.	https://github.com/Bruce-ywj/ERNIE-RNA
Proteomics	TAPE	BERT-based	31 million protein sequences from Pfam	https://github.com/songlab-cal/tape
	ESM-1b	BERT-based	250 million protein sequences from UniRef50	https://github.com/facebookresearch/esm
	ProtTrans	Transformer-XL, XLNet, BERT, Albert, Electra, T5	About 2.3 billion protein sequences from UniRef and BFD	https://github.com/agemagician/ProtTrans
	ProtGPT2	GPT-based	50 million protein sequences from UniRef50	https://huggingface.co/docs/transformers/main_classes/trainer
	ProteinBERT	BERT-based	106 million protein sequences with GO annotations from UniRef50	https://github.com/nadavbra/protein_bert
	KeAP	BERT-based	5 million Triplet in the format of (Protein, Relation, Attribute) with nearly 600 k protein, 50 k attribute terms, and 31 relation terms included	https://github.com/RL4M/KeAP
	CaLM	BERT-based	9 858 385 cDNA sequences of 7 model organisms	https://github.com/oxpig/CaLM
	ESM3	BERT-based	~3.15 billion protein sequences and ~236 million protein structures (with function annotations for ~539 million proteins), totaling ~771 billion unique tokens, aggregated from 8 major sources including UniRef, MGnify, JGI, OAS, PDB, AlphaFoldDB, and ESMAtlas	https://github.com/evolutionaryscale/esm
	ProTrek	BERT-based	Over 5 billion proteins, integrating 7 databases: SWISS-PROT, UniRef50, Protein Data Bank, Open MetaGenomic (OMG), MGnify, global ocean microbiome protein catalog, National Center for Biotechnology Information	https://github.com/westlake-repl/ProTrek
	S^2^ALM	BERT-based	Overall pretraining scale (summary statement in paper): 75 million 1D sequences +11.7 million 3D structures	https://github.com/yashasdevasurmutt/S2ALM
Drug discovery	SMILES-BERT	BERT-based	Two datasets from NCATS (NIH) and 128 datasets from PubChem	https://github.com/uta-smile/SMILES-BERT
	ChemBERTa	BERT-based	77 million unique SMILES	https://github.com/seyonechithrananda/bert-loves-chemistry
	UniMoT	GPT-based	MoleculeNet (~700 K compounds); PubChem (>77 M SMILES); Mol-Instructions (~706 K instructions)	http://uni-mot.github.io
	Mole-BERT	BERT-based	2 million molecules	https://github.com/junxia97/Mole-BERT
	MolGPT	GPT-based	Datasets from MOSES and GuacaMol	https://github.com/devalab/molgpt
	ProtBERT	BERT-based	Datasets from Uniref50, UniRef100, and BFD	https://github.com/agemagician/ProtTrans/
	DeepDDS	BERT-based	Datasets from NCI-ALMANAC	https://github.com/sorachel/DFFNDDS
	SynerGPT	GPT-based	Datasets from DrugCombDB	Code will be made available upon publication
	DrugGPT	GPT-based	Pretraining on ZINC20 with ~2 billion molecular compounds	https://github.com/LIYUESEN/druggpt
	DrugReAlign	GPT-based	Pretraining on four drug-target interaction networks covering enzymes, ion channels, GPCRs, and nuclear receptors (total: 932 drugs, 989 targets, and 5127 interactions)	https://github.com/kkkayle/DrugReAlign
	DrugCLIP	Dual-encoder representation model	Pretraining on drug–disease interaction data from DrugBank, ZINC, and ICD-10, comprising 4803 positive pairs and 9606 synthesized negative pairs	unavailable
Single-cell analysis	scBERT	BERT-based	1 126 580 cells from 209 datasets across 74 tissues and 451 513 cells from 4 sequencing platforms	https://github.com/TencentAILabHealthcare/scBERT
	scGPT	GPT-based	33 million human cells from the CellXGene collection	https://github.com/bowang-lab/scGPT
	Geneformer	BERT-based	29.9 million human single-cell transcriptomes	https://huggingface.co/ctheodoris/Geneformer
	scFoundation	BERT-based	About 50 million human single-cell transcriptomic profiles	https://github.com/biomap-research/scFoundation
	tGPT	GPT-based	22.3 million single-cell transcriptomes	https://github.com/deeplearningplus/tGPT
	GeneCompass	BERT-based	Over 120 million single-cell transcriptomes from humans and mice	https://github.com/xCompass-AI/GeneCompass
	scMulan	GPT-based	More than 10 million manually annotated single-cell RNA-seq data	https://github.com/SuperBianC/scMulan
	UCE	BERT-based	300 datasets from the CellXGene corpus include over 36 million cells, 1000+ cell types, dozens of tissues, and 8 species	https://github.com/snap-stanford/uce
	scPRINT	BERT-based	More than 50 M cells from the CellXGene database	https://github.com/cantinilab/scPRINT
	CancerFoundation	BERT-based	50 million cells with roughly a quarter being tumor cells	https://github.com/BoevaLab/CancerFoundation
	GLEmLN	Graph Transformer	11 M scRNA-seq profiles spanning 19 K genes from the CELLxGENE	https://github.com/czi-ai/GREmLN
	Nicheformer	BERT-based	57 million dissociated and 53 million spatially resolved cells across 73 tissues from both human and mouse	https://github.com/theislab/nicheformer
	Novae	Graph Attention Network (GAT)-based	A large dataset of nearly 30 million cells across 18 tissues	https://github.com/MICS-Lab/novae
	stFormer	BERT-based	0.58 million of two human Visium datasets	https://github.com/csh3/stFormer
	scGPT-spatial	GPT-based	SpatialHuman30M (30 million human cells and spots from 4 sequencing protocols: Visium, Visium HD, MERFISH, and Xenium)	https://github.com/bowang-lab/scGPT-spatial

### RNA foundation models

RNA foundation models are trained using a wide variety of RNA types, including noncoding RNAs (ncRNAs), coding RNA, and untranslated regions (UTRs), across diverse organisms (Table 2). For instance, RNABERT [7], RNA-FM [3], RNA-MSM [4], and UNI-RNA [35] focus on all ncRNA types from a broad range of species, enabling insights into RNA function and interactions. Models like SpliceBERT [36] specialize in coding RNA sequences from 72 vertebrates, while 3UTRBERT [37] is specifically designed for human mRNA transcripts, particularly the 3′ untranslated regions.

### Protein foundation models

Protein foundation models can generate high-quality protein embeddings and support various downstream applications (Table 2). For example, TAPE [38] made a significant contribution by introducing a comprehensive benchmark for protein bioinformatics tasks. ESM-1b [39] applied the transformer architecture of LLMs in a highly standardized manner to protein representation learning. ProtTrans [10], compared to ESM-1b, significantly expanded model size and training data and is widely used as a frozen encoder for protein sequences. ProtGPT2 [40] extends GPT-2 into the protein domain. Recent foundation models like ProtBert [41] and KeAP [42] integrate biomedical text information alongside protein sequences. CaLM [13] represents proteins using cDNA and embeds cross-omics biological information.

### Drug discovery foundation models

Drug discovery foundation models exploit advanced tokenization, embedding, and pretraining strategies to enhance molecular representation learning, supporting diverse downstream tasks (Table 2). Mol-BERT [43] encodes atoms into chemically meaningful discrete values using a context-aware tokenizer, while SMILES-BERT [44], a semi-supervised transformer, is pretrained on datasets like LogP, PM2, and PCBA-686978 via a Masked SMILES Recovery task, enabling robust generalization for molecular property prediction. Mol-GPT [45] facilitates the generation of molecules with specific scaffolds and desired properties by conditioning on scaffold SMILES and property values. SynerGPT [46] extends pretrained GPT models to in-context learning of drug synergy functions, highlighting potential applications in personalized combination therapies.

### Single-cell foundation models

Single-cell foundation models are transforming single-cell analysis by providing scalable solutions that leverage both cell- and gene-level representations (Table 2). Models such as scBERT [17], tGPT [16], scMulan [24], UCE [47], and CancerFoundation [48] focus on learning robust cell embeddings, enabling tasks like cell clustering, cell type annotation, batch effect correction, trajectory inference, and drug response prediction. Models like scGPT [18], scFoundation [23], Geneformer [15], GeneCompass [49], and scPRINT [50] integrate cell- and gene-level representations, modeling inter-gene relationships and regulatory networks for applications including gene expression profiling, Gene Regulatory Network (GRN) inference, gene perturbation prediction, and drug dose–response prediction. scGPT further supports the integration of scRNA-seq and scATAC-seq. For spatial transcriptomics, Nicheformer [51], stFormer [52], and scGPT-spatial [53] excel at learning cell embeddings for spatial label prediction.

## Applications of language models in bioinformatics

**Figure 4:**
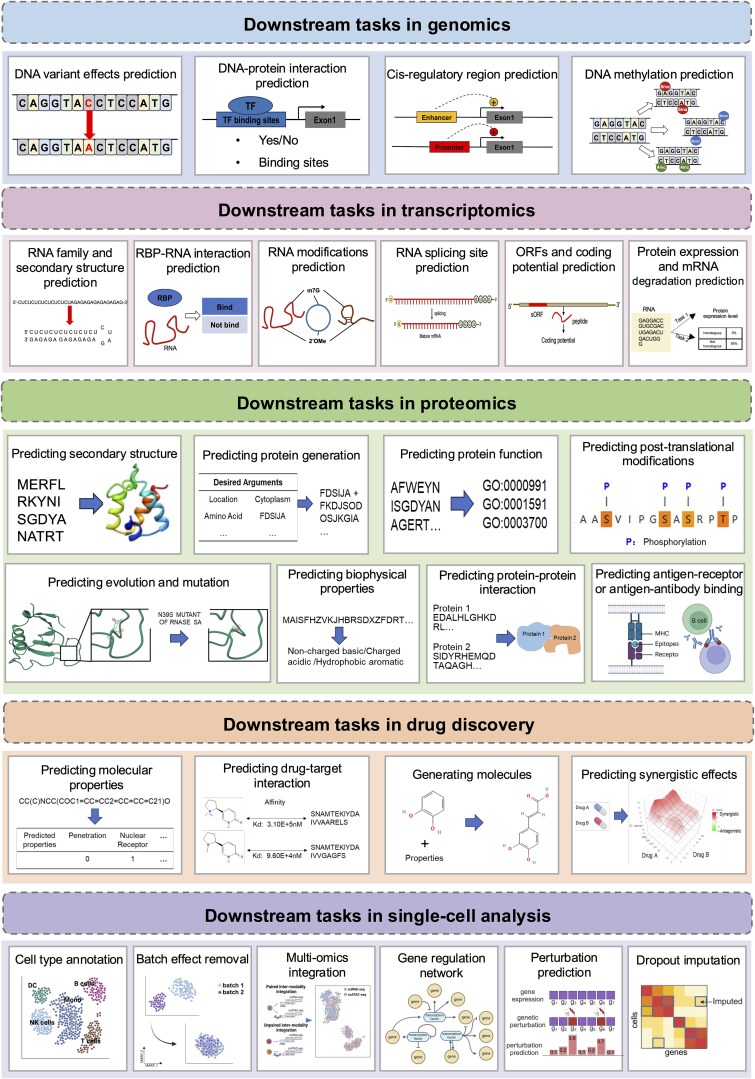
Downstream tasks of language models in bioinformatics. Language models have seen numerous successful applications in bioinformatics, addressing a wide array of tasks across DNA, RNA, protein, drug discovery, and single-cell analysis.

With both theoretical foundations and model architectures established, we now examine how language models are applied to address key challenges across bioinformatics, spanning DNA, RNA, protein, drug discovery, and single-cell analysis (Fig. 4).

### Applications of language models in genomics

The DNA language models take DNA sequence as input and are pretrained using language-model-style objectives (e.g. masked or autoregressive nucleotide prediction) to learn sequence representations, enabling a range of biological tasks, including genome-wide variant effects prediction, DNA *cis*-regulatory regions prediction, DNA–protein interaction prediction, DNA methylation (6mA, 4mC, 5hmC) prediction, and splice sites prediction from DNA sequence (Table 3, Supplementary Fig. 2). While LLMs provide a flexible framework for modeling genomic sequences, their utility is strongly task-dependent and constrained by the biological complexity of genome regulation.

#### Genome-wide variant effects prediction

Genome-wide association studies (GWAS) identify trait-associated loci but often fail to resolve causal variants [32, 54]. Models like DNABERT [5], DNABERT-2 [33], and the Nucleotide Transformer [55] use masked-language pretraining on genomic sequences to predict variant impact, capturing local sequence patterns effectively. Evolution-aware models like GPN-MSA [56] leverage multi-species genome alignments to improve deleteriousness prediction, outperforming single-sequence approaches for coding and noncoding variants. Evo and its scaled variants exemplify large-scale DNA LLMs capable of zero-shot variant effect prediction across extensive sequence collections [57, 58], while hybrid approaches such as JanusDNA [59] explore bidirectional pretraining for long-context representation. Sequence-to-function models, including Borzoi [60] and AlphaGenome [61], further predict transcriptional outcomes and integrate long-range genomic context, highlighting the interplay between LLM representations and functional interpretation. Nevertheless, zero-shot LLMs often underperform evolution-informed methods for rare or cell-type-specific variants, underscoring the continued relevance of comparative genomics.

#### *Cis*-regulatory regions prediction

Accurate identification of promoters and enhancers is critical for understanding gene regulation [62, 63]. Pretrained models such as DNABERT [5], DNABERT-2 [33], GROVER [34], and DNAGPT [6] predict promoter regions and their activity, with BERT-Promoter [64] further improving performance via SHAP-based feature selection. Enhancer prediction benefits from hybrid transfer learning approaches, exemplified by iEnhancer-BERT [65], while models such as Enformer [66] capture long-range enhancer–promoter interactions. Despite promising results, *cis*-regulatory prediction remains challenging due to sparse annotations, cell-type specificity, and complex 3D chromatin interactions, limiting LLM generalization.

#### DNA–protein interaction prediction

Accurate identification of DNA–protein interactions is crucial for gene expression regulation and understanding evolutionary processes [67]. Language models such as DNABERT [5], DNABERT-2 [33], and GROVER [34] are pretrained on ChIP-seq data to capture DNA-binding patterns. Models like TFBert [68] and MoDNA [69] explicitly incorporate sequence motifs and domain knowledge to enhance promoter and transcription factor binding site predictions. While these models effectively extract semantic sequence representations, prediction accuracy depends on motif density, training data diversity, and representation of distal interactions.

#### DNA methylation prediction and splice site identification

Post-replicative methylation and accurate pre-mRNA splicing are critical epigenetic and posttranscriptional processes [70–72]. Language models such as BERT6mA [73], iDNA-ABT [74], iDNA-ABF [75], and MuLan-Methyl [76] predict 6mA, 5hmC, and 4mC methylation sites using multi-scale and multi-model transformer architectures. DNABERT [5] and DNABERT-2 [33] identify splice donor/acceptor sites, revealing attention to intronic regulatory regions, yet their sensitivity remains constrained by motif redundancy and limited representation of alternative splicing events.

The landscape of genomic language models remains highly fragmented, with performance strongly dependent on the biological task rather than on any universal model. Large-scale benchmarks consistently show that effective model selection requires aligning task objectives with model inductive biases, rather than relying on aggregate metrics [77–80]. Comparisons across DNA foundation models, such as Caduceus-Ph and GENA-LM, reveal trade-offs between context length, sequence resolution, and task specificity [81, 82]. Models optimized for long-range dependencies, like context-extended Nucleotide Transformer variants, excel at tasks involving distal regulatory interactions and chromatin-level features. However, extended context does not uniformly improve performance: for tasks driven by localized sequence signals, such as splice-site detection, motif-level binding, or pathogenic variant classification, short-context, base-pair-resolution models (e.g. DNABERT-2) often perform equally well or better. Evolutionary information remains critical: alignment-based or conservation-aware approaches (e.g. GPN-MSA [56], CADD [83]) outperform single-sequence DNA language models for zero-shot variant effect prediction, highlighting limitations of context-agnostic models in capturing cell-type-specific regulatory effects. Benchmarking studies further indicate that pretrained embeddings alone rarely provide universally transferable regulatory representations; downstream performance depends largely on task-specific adaptation, fine-tuning, and evaluation design [79, 84]. These observations underscore the need for task-aware, evidence-driven selection of genomic language models, alongside careful consideration of computational cost and interpretability.

**Table 3:** Language models for downstream tasks in bioinformatics.

**Input data**	**Biological tasks**	**Models**
DNA sequence	Genome-wide variant effects prediction	DNABERT, DNABERT-2, GPN, Nucleotide Transformer, GENA-LM, Evo/Evo2
	DNA cis-regulatory regions prediction	DNABERT, DNABERT-2, BERT-Promoter, iEnhancer-BERT, Nucleotide Transformer, GENA-LM
	DNA-protein interaction prediction	DNABERT, DNABERT-2, TFBert, GROVER, and MoDNA
	DNA methylation (6mA, 4mC, 5hmC) prediction	BERT6mA, iDNA-ABF, iDNA-ABT, and MuLan-Methyl
	RNA splice sites prediction from DNA sequence	DNABERT, DNABERT-2
RNA sequence	RNA 2D/3D structure prediction	RNA-FM, RNA-MSM, RNABERT, RiNALMo, ERNIE-RNA
	RNA structural alignment, RNA family clustering	RNABERT, RNA-MSM, RNA-FM
	RNA splice sites prediction from RNA sequence	SpliceBERT, UNI-RNA
	RNA N7-methylguanosine modification prediction	BERT-m7G, RNAErnie
	RNA 2′-O-methylation modifications prediction	Bert2Ome, RNAErnie
	Multiple types of RNA modifications prediction	Rm-LR, RNAErnie
	Predicting the association between miRNA, lncRNA, and disease	BertNDA, RNAErnie
	Identifying lncRNAs	LncCat, RNAErnie
	Protein expression and mRNA degradation prediction	CodonBERT
Protein sequences MSAs Gene ontology annotations Triplets of protein-relation-attribute Protein property descriptions cDNA sequences	Secondary structure and contact prediction	MSA Transformer, ProtTrans, SPRoBERTa, TAPE, KeAP, ESM3
	Protein sequence generation	ProGen, ProtGPT2, ESM3
	Protein function prediction	SPRoBERTa, ProtST, PromptProtein, CaLM, ESM3, ProTrek
	Major PTMs prediction	ProteinBERT
	Evolution and mutation prediction	SPRoBERTa, UniRep, ESM-1b, TAPE, PLMsearch, DHR, ESM3, ProTrek
	Biophysical properties prediction	TAPE, PromptProtein, ProTrek
	Protein–protein interaction and binding affinity prediction	KeAP
	Antigen-receptor binding prediction	MHCRoBERTa, BERTMHC, TCR-BERT, SC-AIR-BERT, Antiformer
	Antigen–antibody binding prediction	AbLang, AntiBERTa, EATLM, S^2^ALM
Molecular SMILES	Predicting molecular properties	SMILES-BERT, ChemBERTa, K-BERT
	Generating molecules	MolGPT, UniMoT
Molecular graphs	Predicting molecular properties	MOLE-BERT
Molecular fingerprints and protein sequences	Predicting drug–target interaction	TransDTI, FG-BERT, DrugGPT, DrugReAlign, DrugCLIP
Molecular SMILES and protein sequences	Predicting synergistic effects	SynerGPT, C2P2
scRNA-seq data	Cell clustering	tGPT, scFoundation, UCE, iSEEEK, CellPLM, BioFormers, mcBERT
	Cell type annotation	scBERT, scGPT, CIForm, TOSICA, scTransSort, TransCluster, Geneformer, GeneCompass, scMulan, CellLM, CellPLM, scPRINT, GLEmLN
	New cell type identification	scBERT, TOSICA, UCE, MarsGT
	Batch effect removal	scBERT, scGPT, CIForm, TOSICA, Geneformer, scMulan, iSEEEK, scPRINT, CancerFoundation, mcBERT, CellPLM
	Trajectory inference/Pseudotime analysis	tGPT, scMVP, iSEEEK
	Drug response/sensitivity prediction	scFoundation, CellLM, CancerFoundation
	Gene network inference	scGPT, Geneformer, GeneCompass, iSEEEK, scGREAT, BioFormers, scPRINT, GLEmLN
	Gene perturbation prediction	scGPT, scFoundation, GeneCompass, CellPLM, BioFormers
	Gene expression prediction/Imputation/Denoising	scGPT, scMVP, scFoundation, GeneCompass, CellPLM, BioFormers, scPRINT
	cis-regulatory element identification	scMVP
	Drug dose–response prediction, Gene dosage sensitivity prediction	GeneCompass
scMulti-omics data	Single-cell multi-omics integration	scGPT, scMVP, DeepMAPS, scCLIP
	Biological network inference	DeepMAPS
	Cell–cell communications	
	Translating gene expression to protein abundance	scTranslator, scMoFormer
	Single-cell multimodal prediction	scMoFormer
	Integrative regulatory inference	scTranslator
Single-cell spatial transcriptomics data	Spatial transcriptomics imputation	CellPLM, Nicheformer, SpaFormer
	Spatial clustering and label prediction	Nicheformer, Novae**,** stFormer, SpaGT, scGPT-spatial
	Spatial neighborhood density/composition prediction	Nicheformer
	Gene function prediction	stFormer

### Applications of language models in transcriptomics

The RNA language models take RNA sequences as input and are pretrained using language-model-style objectives to learn sequence representations. Representative models include RNABERT, RNA-FM [3], RNAErnie [8], and HydraRNA [85], which are applied to diverse tasks such as RNA structure prediction and alignment, RNA family clustering, RNA splice site prediction, multiple types of RNA modification prediction, predicting the association between miRNA, lncRNA and disease, identifying lncRNAs and their coding potential, protein expression, and mRNA degradation prediction (Table 3, Supplementary Fig. 2).

#### Secondary structure prediction

Models such as RNABERT [7], RNA-MSM [4], RNA-FM [3], ERNIE-RNA [86], RiNALMo [87], and UNI-RNA [35] utilize sequence and structural information to predict base-pairing, stem loops, pseudoknots, and long-range folding patterns. While these models improve understanding of RNA folding and function, comparative studies suggest that predicted structure often adds only marginal gains over sequence-only representations, highlighting potential redundancy in current structure-aware pipelines [88].

#### RNA splicing prediction

RNA splicing is crucial for gene expression in eukaryotes, and advancements have been made in sequence-based splicing modeling through models like SpliceBERT [36] and UNI-RNA [35]. They capture long-range dependencies and structural features to identify splice sites and alternative splicing events, informing gene regulation and splicing-related disease mechanisms.

#### lncRNAs identification and lncRNAs’ coding potential prediction

lncRNA identification and coding potential prediction rely on task-specialized transformers, such as LncCat [89] and LSCPP-BERT [90], which outperform general embeddings by incorporating ORF-aware or species-specific features.

#### RNA-RBP and RNA–RNA interaction prediction

BERT-RBP [91] predicts RNA-RBP interactions from sequence alone, outperforming existing methods on eCLIP-seq datasets across 154 RBPs, while RNAErnie [8], combined with a hybrid network (CNN, Bi-LSTM, and MLP), forecasts RNA–RNA interactions, revealing regulatory network dependencies.

#### RNA modification prediction

Posttranscriptional RNA modifications, such as N7-methylguanosine (m7G) and 2′-O-methylation (Nm), regulate gene expression and are linked to diseases [92, 93]. Identifying modification sites is essential but challenging, as experimental methods are costly and time-consuming. Computational tools like BERT-m7G [94] and Bert2Ome [95] address this issue. BERT-m7G uses a stacking ensemble approach to identify m7G sites, while Bert2Ome combines BERT and CNN to predict 2′-O-methylation, outperforming previous methods in accuracy and scalability, but remains limited by sparse experimental datasets.

#### Protein expression and mRNA degradation prediction

mRNA vaccines are a cost-effective, rapid, and safe alternative to traditional vaccines, showing high potency [96]. These vaccines work by introducing mRNA that encodes a viral protein. CodonBERT [97], trained on 10 million mRNA sequences using a multi-head attention transformer, predicts protein expression and mRNA stability, optimizing mRNA vaccine design and enhancing efficacy.

#### 5′ UTR-based mean ribosome loading prediction and mRNA subcellular localization prediction

The 5′ UTR sequence plays a critical role in regulating translation efficiency. RNA sequence models like 3UTRBERT [37], UNI-RNA [35], UTR-LM [98], RNA-FM [3], and Nucleotide Transformer [55] have been developed to predict key features of the 5′ UTR, focusing on ribosome loading efficiency and mRNA localization. These models analyze sequence patterns, motifs, and structural elements to predict ribosome loading efficiency and subcellular mRNA localization, providing insights into translation control, RNA stability, and spatial regulation of gene expression.

Despite these successes, benchmarking studies reveal that performance is highly task-dependent [99, 100]. Large, general-purpose models such as RNA-FM and UNI-RNA provide broadly transferable embeddings but often underperform task-specialized architectures on narrowly defined problems, such as RNA modification prediction, RBP binding, and splice-site identification [99, 101]. Generative RNA models (RNA-GPT-style) show promise for sequence generation and exploratory representation learning, yet their utility for supervised functional prediction remains largely untested [102, 103].

In practice, model choice should align with the biological objective. Foundation models are advantageous for exploratory analyses, cross-task embedding transfer, or multi-task generalization [99, 100]. In contrast, task-specialized RNA transformers remain preferable when high accuracy is required for a defined prediction problem, such as modification site identification, splice site modeling, or translation efficiency optimization. These insights underscore the ongoing importance of task-aware, evidence-driven model selection in transcriptomic language model applications [100, 101].

### Applications of language models in proteomics

Pretrained protein language models (PPLMs) and pretrained antibody language models (PALMs) learn rich, context-aware representations of protein and antibody sequences from large-scale datasets. In proteomics, PPLMs are applied to protein structure prediction, sequence generation, function annotation, posttranslational modification (PTM) prediction, mutation effect assessment, biophysical property modeling, protein–protein interaction analysis, and binding affinity estimation. PALMs, trained on curated antibody repertoires such as the Observed Antibody Space (OAS), specialize in immune-related applications, including paratope prediction, antigen-receptor and antigen–antibody binding specificity, B cell maturation analysis, antibody classification, and sequence restoration (Table 3, Supplementary Fig. 3).

#### P‌PLM models and related tasks

Protein modeling and structure prediction remain central. Early PPLMs, such as MSA Transformer [104], leverage interleaved attention over multiple sequence alignments to efficiently improve secondary structure and contact prediction. Scaling-focused models such as ProtTrans [10] and ESM3 [104] demonstrate that larger models and datasets enhance per-residue prediction, while multimodal approaches (e.g. ESM3, CaLM [13]) integrate sequence, structure, and functional annotations for more robust embeddings. Sequence generation models like ProtGPT2 [40] and ProGen [11] facilitate protein design, conditioning sequences on functional tags and generating novel, well-folded proteins validated by AlphaFold. Functional annotation is further supported by models such as ProtST [105] and ProTrek [106], which align sequences, structures, and textual functions via multimodal contrastive learning. PTM and mutation prediction benefit from ProteinBERT [41], UniRep [107], and ESM-1b [39], while biophysical property prediction is benchmarked via TAPE [38] and enhanced with prompt-based strategies such as PromptProtein [108]. Protein–protein interactions and binding affinity are modeled by KeAP [42], employing triplet-based encoding and cross-attention.

Several comparative insights emerge. First, BERT-style architectures dominate PPLMs due to their intuitive pretraining objectives and strong transferability to residue-level tasks, whereas GPT-style models are better suited for generative or design-centric applications. Second, scaling both model parameters and pretraining datasets generally improves downstream performance, but gains are task-dependent. Third, multimodal integration of sequence, structure, and functional text is increasingly central, enabling modular architectures and biologically informed pretraining objectives. These trends underscore the importance of aligning model choice with specific biological tasks (Supplementary Table 5).

#### PALM models and related tasks

Even though antibodies are classified as proteins, the datasets of antibodies and subsequent tasks differ significantly from those of proteins. The OAS database [109] provides a large, continuously updated repository of antibody sequences, enabling the development of PALMs. These models address tasks in therapeutic antibody binding, immune repertoire evolution, and antibody discovery, including paratope prediction, B cell maturation analysis, and sequence classification. In antigen-receptor and antigen–antibody binding prediction, MHCRoBERTa [110] predicts pMHC-I binding from amino acid sequences, while BERTMHC [111] focuses on pMHC-II binding. For adaptive immune receptor specificity, TCR-BERT [112] predicts antigen binding from TCR CDR3 sequences but ignores chain interactions. SC-AIR-BERT [113] addresses this limitation and improves the prediction of both TCR and BCR binding. Antiformer [114] integrates RNA-seq and BCR-seq data within a graph-based framework for antibody development. Antibody-focused PALMs include AbLang [115], which restores missing residues during sequencing; AntiBERTa [116], trained for immunogenicity and binding-site prediction; and EATLM [117], which introduces pretraining tasks such as Ancestor Germline Prediction and Mutation Position Prediction, establishing a benchmark for antibody LMs. Antigen–antibody binding prediction is further advanced by S^2^ALM [118], an ESM-2-based BERT-style masked language model that incorporates structural information via Foldseek 3Di tokens, achieving state-of-the-art performance across multiple antibody tasks. These advances highlight PALMs’ growing role in precision immunology and therapeutic antibody design.

PALMs share a broadly similar development trajectory and set of methodological characteristics with PPLMs, including large-scale pretraining and task-specific adaptation for diverse immunological applications. Model selection for specific antibody-related tasks can be guided by the comparative results summarized in Supplementary Table 5.

### Applications of language models in drug discovery

Drug discovery is resource-intensive and has low success rates, particularly for small-molecule therapeutics, which constitute the majority of approved drugs. Transformer-based language models have emerged as tools to accelerate early-stage discovery by learning latent molecular patterns from SMILES strings, graph-derived sequential encodings, or chemical fingerprints, supporting molecular property prediction, *de novo* molecule generation, drug-target interaction (DTI) prediction, and drug synergy inference (Table 3, Supplementary Fig. 4).

#### Molecular property prediction

Accurate ADMET, PK, and physicochemical property prediction is critical for selecting drug candidates [119, 120]. Early LLMs such as SMILES-BERT [44] replaced classical fingerprints with sequence-based embeddings, while ChemBERTa [121, 122] explored the effects of dataset scale, tokenization, and pretraining. UniMoT [14] further frames molecular property prediction as a comprehension task, tokenizing molecules as a “foreign language” to achieve state-of-the-art results. Graph-aware models like Mole-BERT [123] integrate atom-level masking and contrastive learning to capture intrinsic molecular “grammar,” demonstrating that LLMs can encode structural and chemical patterns. However, performance remains constrained by sparse and heterogeneous annotations, especially for rare or safety-critical endpoints, highlighting the continuing importance of careful dataset curation and task-specific adaptation.

#### *De novo* molecule generation

Models inspired by GPT, such as MolGPT [45] and DrugGPT [124], enable context-aware sequence generation and conditional molecular design. While capable of producing chemically valid and diverse compounds, these models face challenges, including distributional bias, overrepresentation of frequent substructures, and the generation of syntactically correct but chemically implausible molecules. Ensuring drug-likeness, synthesizability, and downstream biological relevance remains an active area of investigation, underscoring the need for integrated validation pipelines.

#### Drug-target interaction prediction

DTI modeling accelerates drug development by predicting binding affinity and selectivity [125, 126]. Transformer-based frameworks, such as DTI-BERT, TransDTI [127], and C2P2 [128], integrate pretrained protein and molecular representations to predict interactions, often improving generalization in small-data settings. DrugReAlign [129] represents an LLM-driven drug repurposing framework that predicts DTIs from a knowledge-based and prompt-driven perspective, rather than relying on task-specific supervised training. DrugCLIP [130] is a novel approach to virtual screening for potential drugs that uses contrastive learning to align representations of binding protein pockets and molecules. Structural information, when available, can be incorporated via 3D convolutions or graph neural networks, but language models remain competitive with sequence-only inputs [131–134]. Interpretability remains a major limitation: most pipelines are black boxes, obscuring which residues, substructures, or interaction motifs drive predictions. Emerging approaches, including structure-aware tokens, attention-based attribution, and cross-modal alignment, provide practical, human-interpretable insights, though an atomic-level mechanistic understanding remains limited.

#### Drug synergy prediction

Combination therapies are increasingly important for complex diseases, yet predicting synergistic effects is challenging due to combinatorial explosion and context dependence [135, 136]. Models such as SynerGPT [46] and DCE-DForest [137] model drug synergy by integrating SMILES-based embeddings with cell-line or gene-expression profiles, capturing higher-order interaction patterns. A dual feature fusion approach [138] further combines hashed atom-pair fingerprints, SMILES encodings, and cell-line gene expression to enhance prediction accuracy. Benchmarks reveal strong dependence on available experimental data and context, limiting extrapolation to rare or novel drug combinations. Nevertheless, in-context learning and instruction-driven LLM frameworks offer scalable strategies for exploring combinatorial therapies with minimal retraining, supporting flexible and biologically consistent predictions.

Across drug discovery tasks, optimal performance depends on task specificity, data quality, and input representation. Foundation models provide broadly transferable embeddings but often underperform task-specialized architectures, particularly for molecular property prediction or conditional generation. Generative language models excel in exploration and design tasks but require careful validation to avoid implausible outputs. Finally, benchmarking highlights persistent challenges in evaluation standardization, interpretability, chemical validity, and generalization, emphasizing the need for principled, task-aligned model selection and integration with domain knowledge to maximize practical utility in early-stage drug discovery.

### Applications of language models in single-cell analysis

Language models have demonstrated significant applications in single-cell analysis, including cell-level, gene-level, and multi-omics tasks (Supplementary Fig. 5). Additionally, this section will explore emerging language models based on spatial transcriptomics (Table 3).

#### Cell-level tasks

Cell-level tasks in single-cell analysis encompass clustering, cell type annotation, novel type discovery, batch effect correction, trajectory inference, and drug response prediction. These tasks typically fine-tune pretrained cell representations. Two main strategies exist: (i) appending a special <cls> token whose embedding, updated through transformer layers, serves as the cell representation; and (ii) generating an embedding matrix where each row corresponds to a cell. The <cls> token approach, as in TOSICA [19], supports probability-based annotation via neural classifiers, while embedding-matrix approaches, exemplified by iSEEK [139], enable clustering, annotation, and trajectory mapping. Models like scBERT [17] and UCE [47] exploit multi-head attention to integrate diverse representation subspaces, capturing subtle distinctions and long-range gene–gene interactions to identify novel states. Batch effects, stemming from species, tissue, operator, or protocol differences, pose persistent challenges. Large pretrained models, such as CIForm [20] mitigate these effects without explicit batch labels, supporting cross-species, multi-organ, and multi-platform integration. Similarly, drug response prediction, structurally akin to cell type annotation, appends a classifier to cell embeddings. Approaches like scFoundation [23] and CellLM [140] leverage robust pretrained representations to improve predictive accuracy across diverse biological contexts.

#### Gene-level tasks

Gene-level tasks in single-cell analysis include expression prediction, GRN inference, perturbation response modeling, and drug dose–response prediction. Transformer-based self-attention mechanisms excel at modeling the context-dependent dynamics of GRNs, identifying which genes influence or respond to others. Models such as Geneformer [15] and scGPT [21] leverage attention matrices to infer regulatory relationships. Geneformer, pretrained on vast single-cell transcriptomes, supports diverse applications, from predicting dosage-sensitive disease genes to modeling chromatin and network dynamics. scGPT functions as a generalizable feature extractor, enabling zero-shot gene expression and perturbation prediction. In scFoundation [23], zero or masked gene expressions are processed via a transformer encoder-decoder and projected to predicted values, informing cell-specific gene graphs for perturbation modeling (e.g. GEARS [141]). Incorporating biological priors further enhances performance: GeneCompass [49] integrates GRNs, promoter data, gene family annotations, and co-expression relationships to improve accuracy across multiple gene-level tasks.

#### scMulti-omics tasks

Multi-omics single-cell analysis aims to integrate transcriptomic, epigenomic, proteomic, and chromatin accessibility data. LLMs provide flexibility for such integration. One approach introduces modality tokens, as in scGPT [18]. Another approach uses modality-specific transformers with shared latent space projections, as implemented in scMVP [142]. Graph-based approaches offer an alternative strategy. For example, DeepMAPS [143] constructs cell and gene graphs to infer networks and cell–cell communication. Cross-modality prediction models, such as scTranslator [144] and scMoFormer [145], extend this idea by using graph transformers and message-passing GNNs. These models predict protein abundance, chromatin accessibility, and other modalities from scRNA-seq. They also incorporate prior knowledge, such as STRING [146], to improve prediction accuracy. These approaches demonstrate that LLMs can extract both global and local features across modalities.

Across single-cell benchmarks (Supplementary Table 5), GPT-based models (e.g. scGPT, scMulan) demonstrate clear advantages mainly in tasks that require global latent alignment and cross-dataset generalization. This is particularly evident in batch effect removal, where scGPT consistently outperforms BERT-style models such as Geneformer and CancerFoundation. In contrast, transformer encoder-based or task-specialized BERT-based architectures perform better in precision-driven tasks. Specifically, for cell type annotation, BERT-based encoder models such as CellPLM and scPRINT demonstrate higher accuracy and robustness than scGPT; for gene expression prediction, GeneCompass and CellPLM outperform the GPT-based approach scGPT; and in gene regulatory network inference, the BERT-style model scPRINT yields superior performance relative to scGPT. These results indicate that current single-cell language models are fundamentally limited by architecture-task mismatch. They are further constrained by insufficient biological inductive priors and weak transferability of frozen embeddings. This highlights the importance of selecting models based on task requirements, rather than relying solely on generative architectures.

#### Spatial transcriptomics tasks

Spatial transcriptomics tasks preserve cells’ native spatial context, providing insights into tissue architecture and cellular interactions. Language models can integrate spatial and molecular features to interpret tissue-specific patterns in high-dimensional data. Nicheformer [51], an LLM for spatial transcriptomics, learns joint representations from multimodal, multi-species, and multi-technology datasets, enabling robust spatial predictions even with limited data. Spaformer [147] addresses two major challenges: encoding spatial coordinates and mitigating data sparsity through transformer-based architectures for spatial information integration and imputation. Graph-based frameworks, like Novae [148], explicitly model local neighborhoods, enabling unsupervised identification of spatial domains while correcting batch effects. SpaGT [149], a spatially informed graph transformer, integrates global self-attention with structure-reinforced local edges to denoise expression data and detect fine-grained spatial patterns. Despite these advances, spatial single-cell language models are nascent, challenged by high dimensionality, sparsity, and irregular tissue structures.

#### Representative applications of scGPT across single-cell tasks

**Figure 5:**
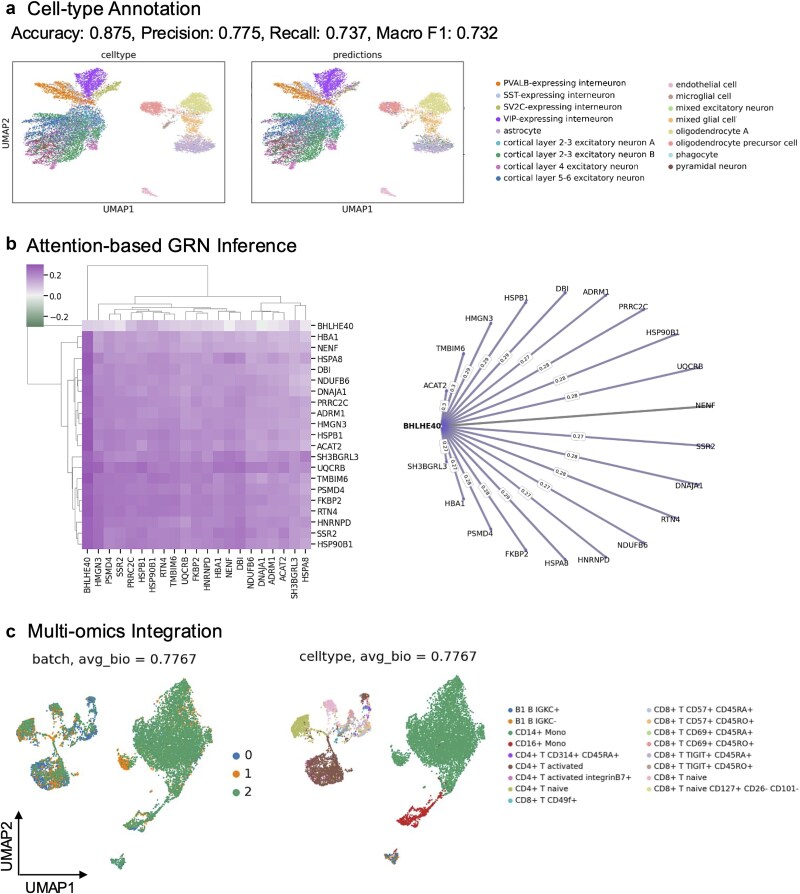
Representative applications of scGPT across single-cell tasks. scGPT is evaluated on three representative tasks spanning cell-level, gene-level, and multi-omics applications. (a) UMAP visualization of ground truth and scGPT-predicted cell types on the multiple sclerosis dataset. (b) Heatmap of the top 20 most influential genes identified by scGPT for GRN inference based on attention-derived signals (left), and the corresponding gene interaction network with edge strengths annotated by rank-normalized attention scores between selected genes and known BHLHE40 target genes (right). (c) UMAP visualization of scGPT-based integration of paired RNA-protein data from the BMMC CITE-seq dataset across three donor batches and multiple immune cell types.

To further illustrate the practical capabilities of single-cell foundation models, we present representative outputs from scGPT across three core tasks, including cell type annotation, gene regulatory network inference, and multi-omics integration (Fig. 5). Figure 5a shows the cell type annotation results on the multiple sclerosis dataset [150], where predicted labels are compared against ground truth annotations. The model achieves Accuracy: 0.875, Precision: 0.775, Recall: 0.737, and Macro F1: 0.732, demonstrating strong agreement with biological labels. For GRN inference, using the fine-tuned model on the Adamson perturbation dataset [151] as an example, we infer cell-state-specific regulatory networks by comparing transcription factor repression (perturbed) and control conditions. scGPT provides attention weights at the single-cell level, which are further aggregated by cell state. Figure 5b shows the top 20 most influenced genes under perturbation versus control, highlighting condition-dependent regulatory rewiring. As a case study, we further examine the overlap between the selected top genes and the known targets of the transcription factor BHLHE40 in external databases, demonstrating the biological consistency of the inferred regulatory signals. Figure 5c illustrates multi-omics integration performance on the BMMC dataset [152] as an example of paired RNA-protein CITE-seq data integration. The dataset includes three donor batches and multiple immune cell subtypes (B, Mono, and T cells). Each cell contains paired transcriptomic and proteomic measurements. The results show that cells from different donors are well mixed in the learned embedding space while preserving clear separation of cell types, indicating successful batch correction and biologically meaningful structure preservation.

## Challenges and perspectives

Despite these advances, several challenges remain that limit the robustness, interpretability, and scalability of language models in biological settings.

### Challenges and limitations

#### Data scarcity, quality, and bias

Most successful biological language models rely on massive, centralized datasets, such as CellXGene for single-cell transcriptomics, UniRef50 for proteins, or large genomic corpora aggregated across species. However, many biologically and clinically important problems operate in regimes of limited, noisy, sparse, or biased data, including rare diseases, patient-specific tumor samples, perturbation screens, and spatial or multi-omic experiments. Across DNA and RNA benchmarks, foundation models pretrained on large generic corpora often transfer poorly to cell-type-specific regulatory tasks or rare variant interpretation when fine-tuning data are scarce [77]. For example, DNABERT performs well at detecting global regulatory signals but shows reduced accuracy for tissue-specific enhancer activity or rare variant effects without substantial task-specific retraining. Cross-species pretraining and parameter-efficient fine-tuning, as used in Nucleotide Transformer, partially mitigate these issues but remain limited in highly cell-type-specific contexts [78, 79]. In single-cell analysis, models such as Geneformer and scFoundation improve robustness by incorporating masked modeling objectives and gene–gene interaction priors. Similar challenges arise in drug discovery, where LLMs trained on public compound-target databases (e.g. ChEMBL [153] or BindingDB [154]) inherit reporting biases and label noise, limiting generalization to novel chemical space.

To address these issues, several data curation and cleaning strategies have proven effective across biological domains. A first widely used approach is rigorous quality control filtering, including removal of low-quality cells or samples (e.g. high mitochondrial content in scRNA-seq, low read depth, or abnormal gene counts) and exclusion of technical artifacts or duplicated entries. A second strategy is batch harmonization and normalization, where methods such as batch-aware scaling, mutual nearest neighbors (MNNs) correction, or latent-space alignment reduce protocol- and platform-induced variation prior to model training. A third approach is bias-aware dataset balancing, including stratified sampling across tissues, species, or disease states to prevent overrepresentation of common cell types or well-studied compounds. In addition, ontology-guided annotation harmonization (e.g. mapping to standardized cell-type or gene-ontology vocabularies) improves label consistency across heterogeneous datasets. For chemical and genomic data, deduplication and structure standardization (e.g. canonical SMILES normalization, RDKit-based canonicalization, and InChIKey-based deduplication) are commonly applied to reduce label noise. Finally, data augmentation and synthetic sampling strategies, such as sequence masking, *in silico* perturbation, or generative oversampling of rare classes, can partially alleviate long-tail distribution issues in underrepresented biological regimes.

#### Computational accessibility and scalability

The computational cost of training and deploying biological language models remains a major barrier to broad adoption and reproducibility [155]. Many state-of-the-art models depend on large parameter counts, extensive GPU resources, and long training cycles. Importantly, the trade-off between model scale, computational cost, and downstream performance has emerged as a central and increasingly critical issue in this field. Several strategies have emerged to mitigate these challenges. One option is to access large foundation models through online or API-based services, lowering the barrier for inference at the cost of ongoing operational expenses and limited control over model internals [156]. A more sustainable solution is parameter-efficient fine-tuning (PEFT), including adapters, LoRA, and prompt-based tuning, which allows large pretrained models to be adapted to new tasks with minimal trainable parameters [157–159]. These approaches have proven effective across domains, from single-cell annotation to protein function prediction [160, 161]. Knowledge distillation and model compression offer an additional pathway [162], enabling smaller, task-specific models to inherit capabilities from large foundation models while substantially reducing training and inference costs.

From a practical perspective, model selection should be guided by the specific task and computational constraints. Large-scale foundation models are most advantageous for tasks that require cross-dataset generalization, multimodal integration, or global representation learning, such as batch effect correction, cross-cohort analysis, and multi-omics integration. In contrast, for well-defined, precision-oriented tasks, such as cell type annotation, gene expression prediction, splicing prediction, or protein-ligand binding, smaller or task-specialized models often achieve comparable or superior performance with significantly lower computational cost. Emerging evidence further suggests that smaller, specialized architectures tailored to specific biological tasks, such as splicing prediction or protein-ligand binding, often outperform general-purpose models while remaining computationally efficient and easier to interpret. These trends indicate that the future of biological language models will depend on modular, task-aware systems rather than indiscriminate scaling.

#### Interpretability, reliability, and model “hallucinations”

Interpretability and trustworthiness remain critical unresolved challenges for biological language models. These models are often treated as black boxes, making it difficult to explain predictions of regulatory interactions, functional annotations, or drug-target associations. This limitation is particularly problematic for high-stakes applications such as drug discovery and precision medicine, where incorrect yet confident predictions can have serious consequences. Moreover, LLMs are prone to hallucinations, generating outputs that are syntactically plausible yet biologically implausible or unsupported by evidence. In genomics, this may appear as spurious regulatory relationships; in protein modeling and drug discovery, it can lead to unrealistic binding hypotheses or chemically infeasible molecules. Recent work in drug-target interaction prediction demonstrates how biologically grounded design choices can partially mitigate these risks. For example, C2P2 [128] transfers interaction knowledge from related biological networks; DrugReAlign [129] incorporates curated drug-target knowledge through prompt-driven reasoning; DrugCLIP [130] aligns molecular representations with binding protein pockets via contrastive learning; and structure-aware models constrain predictions using 3D information. While these approaches do not yield atom-level mechanistic explanations, they restrict the hypothesis space and enable cross-validation with orthogonal evidence, supporting human-in-the-loop screening. More broadly, improving trustworthiness will require biologically grounded interpretability, constraint-aware decoding, tighter integration with mechanistic or physics-based models, and evaluation frameworks that penalize overconfident but incorrect predictions.

**Figure 6:**
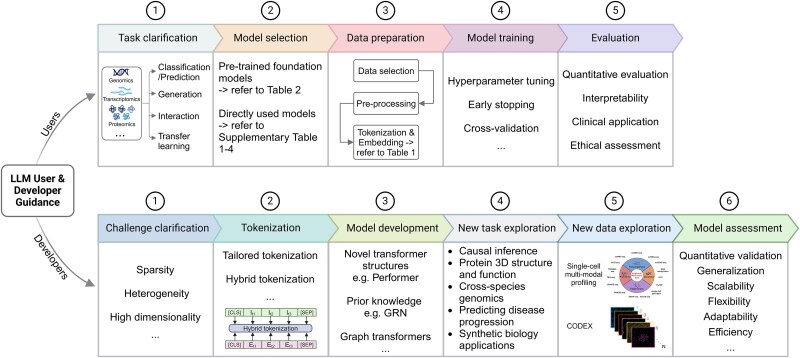
Guidance for users and developers on how to use and develop LLM in practice. Guidance for LLM users includes steps such as clarifying the task, selecting an appropriate model, preparing the dataset, training the model, and evaluating its performance. For LLM developers, the focus involves identifying domain-specific challenges, designing tokenization strategies, advancing model architectures, exploring novel tasks and data types, and assessing model capabilities comprehensively.

Given the limitations outlined above, the effective use of biological language models demands a shift from generic pipelines to task-aware decision-making. Drawing on our experience, we provide practical guidance for both users and developers, emphasizing how to align model choice with biological objectives, integrate multimodal information, and translate representation learning into actionable insight (Fig. 6).

### Perspectives

The rapid adoption of language models in bioinformatics marks a pivotal transition in computational biology, from task-specific predictive models toward more general, integrative reasoning systems. Yet, the most transformative impact of language models lies not in incremental performance but in their potential to reshape how biological knowledge is represented, integrated, and explored. A central frontier is the development of genuinely multimodal biological models that can reason jointly over heterogeneous data types, including genomic and epigenomic profiles, single-cell and spatial transcriptomics, protein structure, imaging data, and clinical narratives. Current approaches often rely on late fusion or simple concatenation of embeddings, which fail to capture the causal and hierarchical relationships linking molecular states to cellular phenotypes and organismal outcomes. Future LLM-based systems must move beyond superficial modality alignment toward architectures that encode cross-scale biological constraints, enabling coherent reasoning across molecular, cellular, tissue, and clinical levels. Such models could, for example, link histopathological image features to cell-state transitions inferred from single-cell data and ultimately to patient-level therapeutic responses. Another major opportunity lies in repositioning language models as engines for biological hypothesis generation, rather than purely predictive tools. By synthesizing knowledge across large corpora of experimental data and literature, language models may help propose testable hypotheses regarding regulatory mechanisms, cell fate decisions, or drug resistance pathways. Crucially, this shift will require models that can express uncertainty, alternative explanations, and causal assumptions, rather than producing single-point predictions. Coupling LLM-driven hypothesis generation with experimental design frameworks, such as perturbation prioritization or adaptive screening, could accelerate discovery while maintaining scientific rigor. Perhaps the most ambitious frontier is the construction of biological digital twins, computational models that simulate the dynamic behavior of cells, tissues, or even organs under genetic, environmental, or therapeutic perturbations. The next phase of biological language models will be defined not by scale alone, but by their ability to integrate multimodal data, generate mechanistic hypotheses, and support dynamic, interpretable models of biological systems.

**Key Points**

- Our study provides a comprehensive overview of large language models (LLMs) in bioinformatics, highlighting their applications across genomics, transcriptomics, proteomics, drug discovery, and single-cell analysis.
- Our study outlines the core technical foundations of LLMs, including tokenization strategies for biological data, transformer architectures, attention mechanisms, and pretraining processes.
- Our study presents an up-to-date overview of existing foundation models and demonstrates their downstream applications in various bioinformatics domains.
- Our study also offers practical guidance for LLM users and developers, emphasizing strategies to enhance performance and promote innovation in biological research.

## Supplementary Material

Supplementary_material_bbag367

## Data Availability

No new data were generated or analyzed in this review.
