# Supplementary material for: Advancing bioinformatics with language models: components, applications, and perspectives
Source: Brief Bioinform. 2026 Jul 10;27(4):bbag367. doi: 10.1093/bib/bbag367 (PMC13354062; doi:10.1093/bib/bbag367)
Supplement: Supplementary_material_bbag367 [file supplementary_material_bbag367.zip › Supplementary figures.docx]

**Supplementary figures**

**
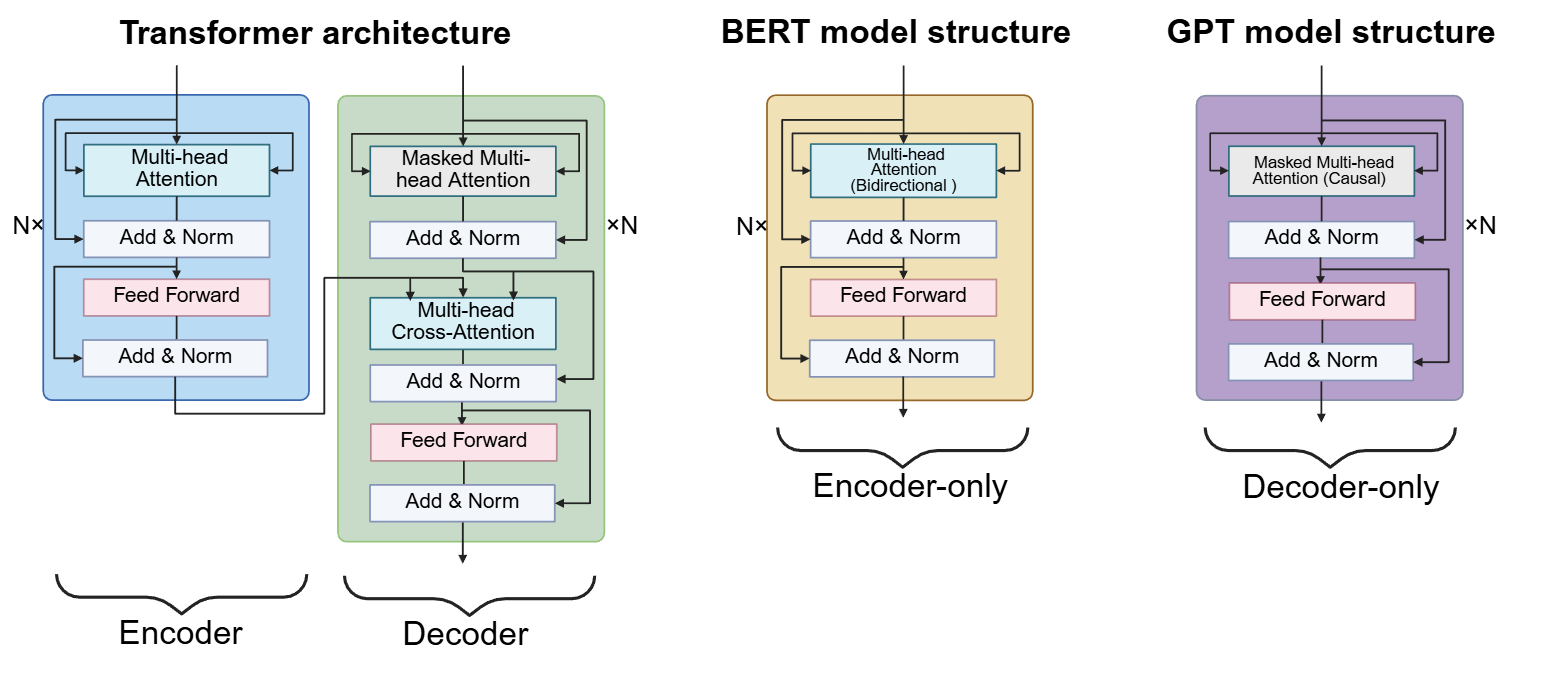
**

**Supplementary figure 1. Comparison of Transformer architecture, BERT model architecture, and GPT model architectures.**


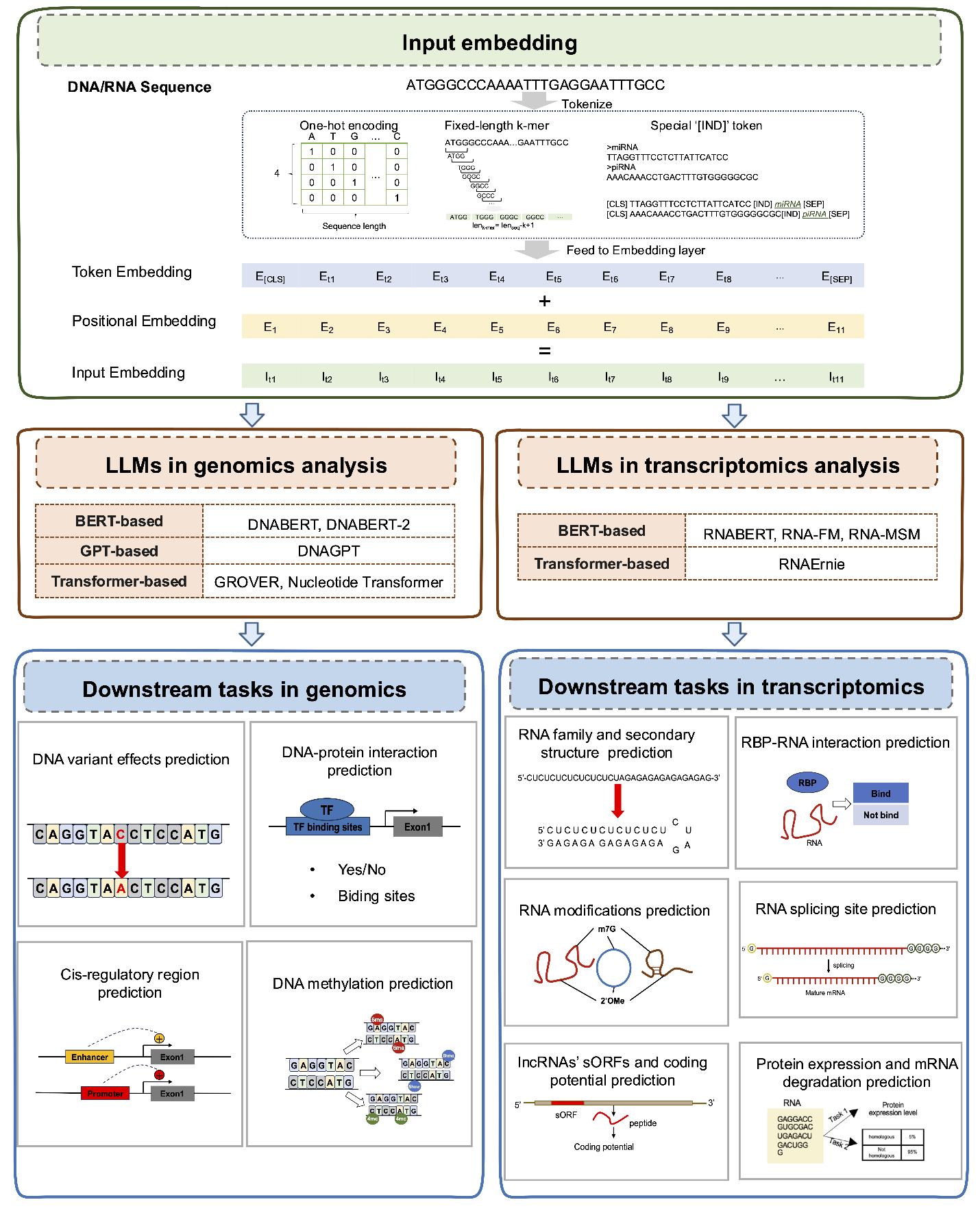


**Supplementary figure 2. Applications of large language models in genomics and transcriptomics.** DNA language models take DNA sequence as input, use transformer, BERT, GPT models to solve multiple biological tasks, including genome-wide variant effects prediction, DNA cis-regulatory regions prediction, DNA-protein interaction prediction, DNA methylation (6mA,4mC 5hmC) prediction, splice sites prediction from DNA sequence. The RNA language models take RNA sequences as input, use transformer, BERT, GPT models to solve multiple biological tasks, including RNA 2D/3D structure prediction, RNA structural alignment,, RNA family clustering, RNA splice sites prediction from RNA sequence, RNA N7-methylguanosine modification prediction, RNA 2’-O-methylation modifications prediction, multiple types of RNA modifications prediction, predicting the association between miRNA, lncRNA and disease, identifying lncRNAs, lncRNAs’ coding potential prediction, protein expression and mRNA degradation prediction.


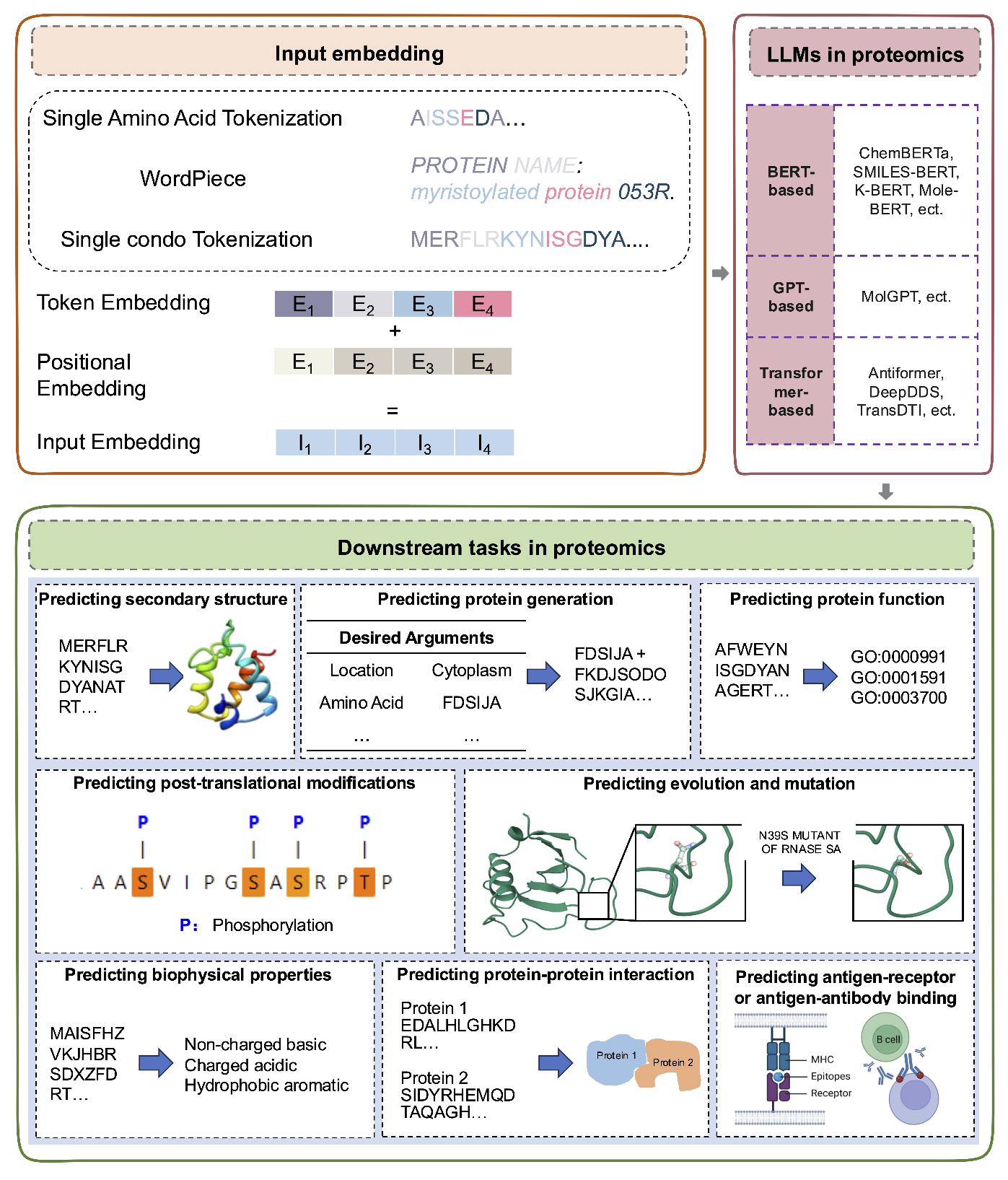


**Supplementary figure 3. Applications of large language models in proteomics.** The protein language models take multiple sequence alignment, protein sequence, gene ontology and protein-relation-attribute as input, use transformer, BERT, GPT models to solve multiple biological tasks, including predicting secondary structure, predicting protein generation, predicting protein function, predicting post-translational modifications, predicting evolution and mutation, predicting biophysical properties, predicting protein-protein interaction and predicting antigen-receptor or antigen-antibody binding.


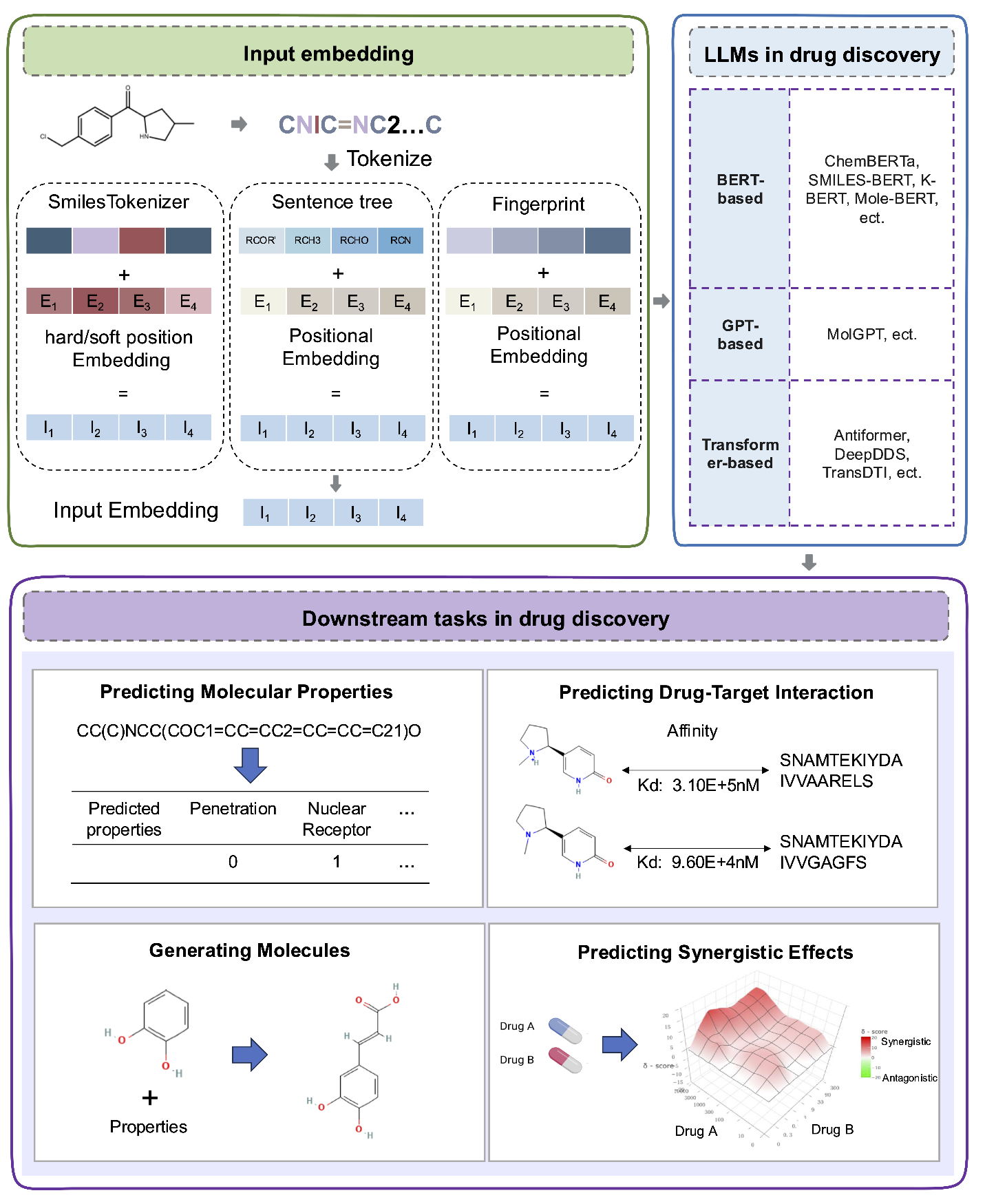


**Supplementary figure 4. Applications of large language models in drug discovery.** The language models for drug discovery take molecular SMILES, protein sequence, molecular fingerprints and molecular graphs as input, use transformer, BERT, GPT models to solve multiple biological tasks, including predicting molecular properties, predicting drug-target interaction, generating molecules and predicting synergistic effects.


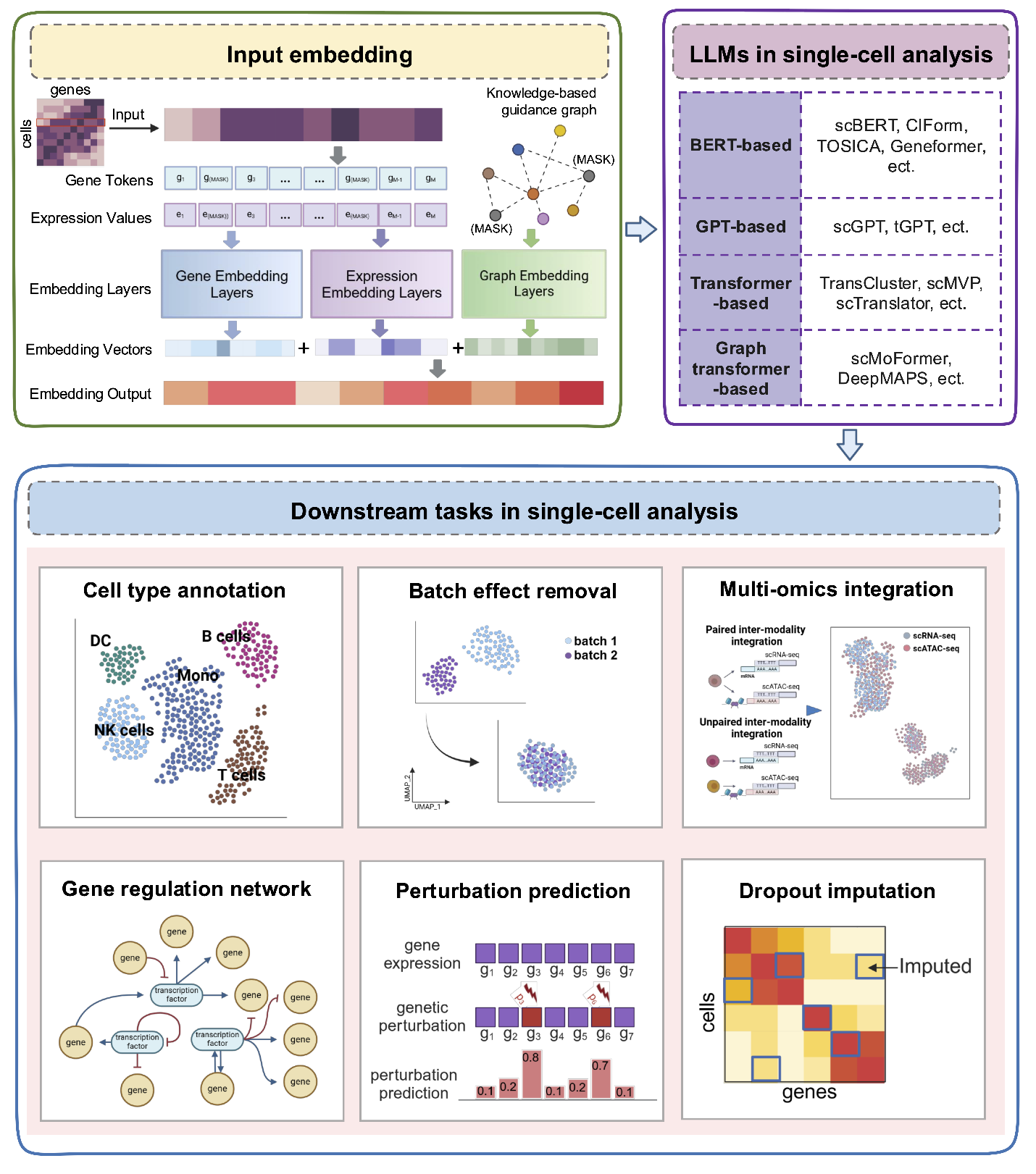


**Supplementary figure 5. Applications of large language models in single-cell analysis.** The single-cell language models take gene expression or single-cell multi-omics data as input, use transformer, BERT, GPT models to solve multiple biological tasks, including cell type annotation, batch effect removal, multi-omics integration, gene regulation network inference perturbation prediction, dropout imputation.
