# Supplementary material for: Advancing bioinformatics with language models: components, applications, and perspectives
Source: Brief Bioinform. 2026 Jul 10;27(4):bbag367. doi: 10.1093/bib/bbag367 (PMC13354062; doi:10.1093/bib/bbag367)
Supplement: Supplementary_material_bbag367 [file supplementary_material_bbag367.zip › Supplementary Table 1.docx]

**Supplementary Table 1. Detailed information of language models for genomic and transcriptomic tasks**

***"Transformer-based" refers exclusively to models using a full encoder-decoder architecture. Models that use only the encoder stack are categorized as "BERT-based", while models using only the decoder stack are categorized as "GPT-based".**

| **Application area** | **Models** | **Ref** | **Publication time** | **Model configuration** | **Architecture** | **Fine-tuning datasets** | | | **Downstream tasks** |
| --- | --- | --- | --- | --- | --- | --- | --- | --- | --- |
|  |  |  |  |  |  | **Data type** | **Source** | **Size** |  |
| DNA sequence language model  RNA sequence language model | DNABERT | [1] | Aug 2021 | 12 layers with 768 hidden units and 12 attention heads in each layer | BERT-based | DNA sequence | Human TATA and non-TATA promoters of 10 000 bp length[2] and ChIP-seq dataset [3] | 3,065 human TATA and 26,533 non-TATA promoter-containing sequences and 690 ChIP-seq dataset covers 161 transcription factor binding profiles in 91 human cell lines | Transcription factor binding sites prediction |
|  |  |  |  |  |  |  | DNA sequence | - | Motif analysis |
|  |  |  |  |  |  |  | Assembly GRCh38 FASTA file [4] | 10,000 donor, acceptor, and non-splice site sequences | Splice donor and acceptor sites prediction |
|  |  |  |  |  |  |  | dbSNP release 153 [5] | 700 million short genetic variants | Identifying effects of genetic variants |
|  | DNABERT-2 | [6] | July 2023 | 12-layer encoder-only Transformer (hidden size 768, 12 attention heads) | BERT-based | DNA sequence | TATA and non-TATA promoters downloaded from Eukaryotic Promoter Database (EPDnew) [2] | 3,065 human TATA and 26,533 non-TATA promoter-containing sequences | Promoter detection and core promoter detection |
|  |  |  |  |  |  |  | ChIP-seq datasets [7] | 161 TF binding profiles in 91 human cell lines(human) and 78 mouse ENCODE ChIP-seq data | Transcription factor binding site prediction |
|  |  |  |  |  |  |  | Ensembl GRCh38 human reference genome [4] | 10,000 splice donors, acceptors, and non-splice site sequences. | Splice site prediction |
|  |  |  |  |  |  |  | Histone modification (Yeast) | H3, H3K14ac, H3K36me3, H3K4me1, H3K4me2, H3K4me3, H3K79me3, H3K9ac, H4, H4ac | Epigenetic marks prediction |
|  |  |  |  |  |  |  | SARS_CoV_2 variants [8] | 9 types of SARS_CoV_2 variants, including *Alpha, Beta,Delta, Eta, Gamma, Iota, Kappa, Lambda and Zeta.* | Covid variant prediction |
|  | Nucleotide Transformer | [9] | Jan 2023 | 2 hidden layers | Transformer-based | DNA sequence | Annotated DNA sequence [10] | 90,000 sequences annotated by Ensembl (“5’ UTR”, “3’ UTR”, “exon”, “intron”, “enhancer”, “promoter”, “CTCF binding site”, “open chromatin”, and “transcription factor binding sites”. ) | Detect known genomic elements |
|  |  |  |  |  |  |  | DNA sequence with SNP [11] | Independent dataset of genetically diverse human genomes, originating from 7 different meta-populations | Detect human genetic variation |
|  |  |  |  |  |  |  | 1000 Genomes Project SNPs [12] | chromosome 22 sequence with 17 variant categories (e.g. stop gained, missense, intergenic) | Predict the impact of mutations |
|  | DNAGPT | [13] | July 2023 | 12 layers of transformer blocks based on unidirectional attention, with each layer containing 12 attention heads and a hidden layer size of 768 | GPT-based | DNA sequence | DNA sequence from DeepGSR [14] | 20,933, 18,693, 12,082, and 27,203 true polyadenylation signals data; and 28,244, 25,205, 17,558, and 30,283 true translation initiation sites for human, mouse, bovine, and fruit fly, respectively which are used as ground-truth, non-genomic signals and regions sequences from the genome sequences and combined them with the true cases | Genomic signals and regions prediction |
|  |  |  |  |  |  |  | DNA sequence from Xpresso [15] | 18,377 and 21,856 promoters as well as the mRNA half-lives in human and mouse respectively and held out 1000 cases in each specie | mRNA expression level prediction |
|  | GROVER | [16] | July 2023 | 12 transformer layers, 5,000 embeddings | BERT-based | DNA sequence | CTCF ChIP-seq data [17] | ~85,000 binding motifs, only ~32,000 are indeed bound by CTCF | Protein-DNA binding prediction |
|  | GPN | [18] | Oct 2023 | 25 convolutional blocks with a feed-forward layer, 512embedding sizes of the pre-trained foundation model | BERT-based | DNA sequence | DNA sequence | - | DNA motifs predictions |
|  |  |  |  |  |  |  | 1001 Genomes Project [12] | 10 million SNPs | Variant effect prediction |
|  | BERT-Promoter | [19] | Aug 2022 | 12 layers, 768-hidden, 12 heads, and 110,000,000 parameters | BERT-based | DNA sequence | ChIP-chip data, gSELEX peaks, ChIP-exo plus RNA-seq [20, 21] | 3382 promoters (1591 strong promoter samples and 1791 weak promoter samples) and 3382 non-promoters | DNA promoter prediction |
|  | TFBert | [22] | Mar 2023 | 12-layer encoder | BERT-based | DNA sequence | ChIP-seq datasets [7] | 690 ChIP-seq dataset contains a training set (80%) and a corresponding test set (20%) | DNA–protein binding sites prediction |
|  | MoDNA | [23] | Aug 2022 | - | BERT-based | DNA sequence | Same experiment data with DNABERT [2, 3] | 3,065 human TATA and 26,533 non-TATA promoter-containing sequences and 690 ChIP-seq dataset covers 161 transcription factor binding profiles in 91 human cell lines | Promoter Prediction |
|  |  |  |  |  |  |  | CHIP-Seq datasets [3] | 690 CHIP-Seq datasets of uniform TFBS contains 161 TFs covering 91 human cell types | Transcription Factor Binding Sites Prediction |
|  | iEnhancer-BERT | [24] | Aug 2022 | 12 layers | BERT-based | DNA sequence | 15 chromatin states of 9 cell types [25] | 2968 samples including 1484 non-enhancers, 742 strong enhancers and 742 weak enhancers | Identifying Enhancers and Their Strength |
|  | BERT6mA | [26] | Mar 2022 | The hidden size is 128 | BERT-based | DNA sequence | Nuclei purification, MNase-seq and ChIP-seq [27-31] | 6mA and non6mA data in 11 species including Arabidopsis thaliana (31873 6mAs and non-6mAs), Caenorhabditis elegans (79616 mAs and non-6mAs), Casuarina equisetifpolia (6066 6mAs and non-6mAs), Drosophila melanogaster (11 191 6mAs and non-6mAs), Fragaria vesca (3102 6mAs and non-6mAs), H. sapiens (18 335 6mAs and non-6mAs), Rosa chinensis (599 6mAs and non-6mAs), Saccharomyces cerevisiae (37866mAs and non-6mAs), Thermus thermophilus (107 600 6mAs and non-6mAs), Ts. SUP5–1 (3379 6mAs and non6mAs) and Xoc. BLS256 (17 215 6mAs and non-6mAs) | DNA N6-methyladenine site prediction |
|  | iDNA-ABF | [32] | Oct 2022 | 12 transformer layers with 768 hidden units and 12 attention heads in each layer | BERT-based | DNA sequence | ChIP-seq data, ATAC-seq data, and histone modifications (HM) data of three human cell lines [33, 34], and DNA methylation dataset from the iDNA-MS [35] | 3 main types of DNA methylation sites (6mA,4mC, and 5hmC) across 12 genomes (1 bacteria and 11 eukaryotes), in total 250,599 positive samples | DNA 6mA, 4mC, and 5hmC prediction |
|  |  |  |  |  |  |  | DNA sequence | - | Motifs analysis |
|  | iDNA-ABT | [36] | Sep 2021 | 12 transformer layers with 12 attention heads in each layer. | BERT-based | DNA sequence | ChIP-seq data, ATAC-seq data, and histone modifications (HM) data of three human cell lines [33, 34] , and DNA methylation dataset from the iDNA-MS [35] | 3 main types of DNA methylation sites (6mA,4mC, and 5hmC) across 12 genomes (1 bacteria and 11 eukaryotes), in total 250,599 positive samples | DNA 6mA,  4mC, and 5hmC prediction |
|  |  |  |  |  |  |  | DNA sequence | - | Motifs analysis |
|  | MuLan-Methyl | [37] | July 2023 | 12 layers in the encoder  stack, 768 hidden units for feed-forward networks, and 12 attention heads. | BERT-based | DNA sequence | ChIP-seq data, ATAC-seq data, and histone modifications (HM) data of three human cell lines [33, 34] , and DNA methylation dataset from the iDNA-MS [35] | 3 main types of DNA methylation sites (6mA,4mC, and 5hmC) across 12 genomes (1 bacteria and 11 eukaryotes), in total 250,599 positive samples | DNA 6mA,  4mC, and 5hmC prediction |
|  | GENA-LM | [38] | Jan 2025 | 12–24 encoder layers, 768–1024 hidden units, and 12–16 attention heads per layer (~110M–336M parameters) | BERT-based | DNA sequence | TSS (transcriptional start sites) from the EPDnew database[39] | Thousands to tens of thousands of promoter sequences, kb-scale per sequence | Promoter prediction |
|  |  |  |  |  |  | DNA sequence | These datasets from DeepSTARR model[40] | thousands of labeled regulatory sequences | Enhancer prediction |
|  |  |  |  |  |  | DNA sequence | DeepSEA dataset[41] | genomic regions with accessibility labels | Chromatin feature accessibility prediction |
|  |  |  |  |  |  | DNA sequence | splice donor and acceptor sites[42] | 5000-bp target region is bracketed by 10 000 bp of context, with 5000 bp on each side | Splice site prediction |
|  |  |  |  |  |  | DNA sequence | APARENT dataset[43] | upstream and downstream segments of the 5’-untranslated regions | Polyadenylation sites prediction |
|  | Evo/Evo2 | [44, 45] | Nov 2024  Feb 2025  biorxiv | ~10B parameters (ultra-long context up to ~131 kb) | GPT- based | DNA sequence | 20 prokaryotic and 16 eukaryotic species were obtained from NCBI [46, 47] | -20nt to +20nt from the first base of the start codon and mutated the wildtype base of each position to each of the three alternative bases to introduce SNVs. | Predict effect of mutations around start codons |
|  |  |  |  |  |  | DNA sequence | Premature stop codons into coding sequences across 5 species | Sequences were randomly subsampled to at most 200,000 per genome. | Predict genetic code usage |
|  |  |  |  |  |  | DNA sequence | cis-regulatory elements (cCREs) from shuffled control sequences; TF binding sites; chromatin accessibility QTLs (CaQTLs) across African populations; Yoruba dynamic sequence QTLs (dsQTLs) | 2.3 million sequences derived from experimentally validated regulatory regions; approximately 577,000 sequences from TF footprinting experiments; chromatin accessibility QTLs (CaQTLs) across African populations,  with over 219,000 variant sites; Yoruba dynamic sequence QTLs (dsQTLs), with  approximately 28,000 sites | Noncoding regulatory sequence prediction including cis-regulatory elements, transcription factor motifs, variant effects |
|  |  |  |  |  |  | DNA sequence | Deep mutational scanning datasets from ProteinGym[48] | ncRNA sequences | protein fitness prediction |
|  |  |  |  |  |  | DNA sequence | Deep mutational scanning datasets on ncRNA | Not explicitly specified | ncRNA fitness prediction |
|  |  |  |  |  |  | DNA sequence | human cell line dataset that leverages metabolic RNA labeling to estimate mRNA  decay rates across the transcriptome | Not explicitly specified | mRNA decay evaluation |
|  |  |  |  |  |  | DNA sequence | 94 available eukaryotic species from PANTHER19.0[49] | Not explicitly specified | Exon/intron classification |
|  |  |  |  |  |  | DNA sequence | Binary essentiality data (labeled as “essential” or “nonessential”) for 56 bacterial genomes | Not explicitly specified | Gene essentiality |
|  |  |  |  |  |  | DNA sequence | Human lncRNA essentiality data | (i) human lncRNA essentiality labels derived from a Cas13 knockdown screen across five cell lines (HAP1, HEK293FT, K562, MDA-MB-231, and THP1), (ii) curated human lncRNA gene annotations, and (iii) genomic DNA sequences centered on Cas13 guide RNA binding sites, including scrambled local mutations and long-range flanking regions extracted from the reference genome. | lncRNA essentiality |
|  |  |  |  |  |  | DNA sequence | Genome-wide variant datasets | Variant effects are assessed in a zero-shot manner using DNA or protein sequence context, evolutionary conservation, and pretrained biological language models. | BRCA1 supervised classification |
|  | RNA-MSM | [50] | Nov 2023 | 12 attention heads with embedding size of 768 | BERT-based | RNA sequence | RNA secondary structure and three-dimensional RNA structures [51] | The training, validation, and test sets have 405, 40, and 70 RNAs. | RNA secondary structure prediction |
|  |  |  |  |  |  |  | RNA secondary structure and three-dimensional RNA structures [51] | The training, validation, and test sets have 405, 40, and 70 RNAs. | RNA solvent accessibility prediction |
|  | RNA-FM | [52] | arXiv posted  Apr 2022 | 12 transformer-based bidirectional encoder blocks and embedding size is 640 | BERT-based | RNA sequence | RNA secondary structure [53, 54] | 37149 structures from 8 RNA types of RNAStralign and 3975 RNA structures from 10 RNA types of ArchiveII | RNA secondary structure prediction |
|  |  |  |  |  |  |  | RNA secondary structure [53, 54] | 37149 structures from 8 RNA types of RNAStralign and 3975 RNA structures from 10 RNA types of ArchiveII | RNA 3D closeness prediction |
|  |  |  |  |  |  |  | whole genome of Severe acute respiratory syndrome coronavirus 2 (SARS-CoV-2) [55] | Whole genome | SARS-CoV-2 genome structure and evolution prediction |
|  |  |  |  |  |  |  | In vivo RNA secondary structure profiles for RNA-protein interaction [56] | - | Protein-RNA interaction prediction |
|  |  |  |  |  |  |  | Human 5’UTR library [57] | 83,919 5’UTRs of 75 different lengths and their corresponding mean ribosome loadings | mRNA 5’ UTR-based mean ribosome loading prediction |
|  | RNABERT | [58] | Feb 2022 | 6 hidden layers of BERT, an embedding layer, one bidrectional-LSTM unit, two dense layers one with ReLU activation and a softmax output layer of LSTM | BERT-based | RNA sequence | RNA (ncRNA) families from RFam database[58] | 31 RNA families | classifying RNA families  RNA secondary structure prediction |
|  | SpliceBERT | [59] | Mar 2024 | 6 transformer encoder layers, 512 hidden layer and 16 attention heads | BERT-based | RNA sequence | Reference genomes in fasta [60] | The pre-mRNA sequences from 72 vertebrate genomes for pre-training | Estimating splice sites |
|  | BERT-m7G | [61] | Aug 2021 | - | BERT-based | RNA sequence | RNA sequence with N7-methylguanosine sites and RNA sequence without N7-methylguanosine sites AlkAniline-Seq, MeRIP-seq, and miCLIP-seq [62] | 741 RNA sequences with N7-methylguanosine sites and 741 RNA sequences without N7-methylguanosine sites | RNA N7-methylguanosine sites prediction |
|  | M6A-BERT-Stacking | [63] | March 2023 | 12 transformer layers with 12 attention heads in each layer. | BERT-based | RNA sequence | RNA sequence with m6A sites and RNA sequence without m6A sites identified from MeRIP, m6A-seq, PA-m6A-seq, and miCLIP [64] | 11 datasets including 3000~ 16000 RNA sequences for each dataset | RNA m6A sites prediction |
|  | Bert2Ome | [65] | May 2023 | 16 heads, 12 layers, and 1024 hidden units | BERT-based | RNA sequence | 2-O-methylation modification sites from RMBase database [66] | 215 positive, 215 negative instances for the training part and 46 positive, 114 negative instances for the testing part. | RNA 2-O-methylation prediction |
|  | Rm-LR | [67] | Sep 2023 | 6 transformer encoder layers, with a hidden layer size of 512 and 16 attention heads | BERT-based | RNA sequence | Transcriptomic-wide profiling data derived from the MultiRM, GEO, RMBase, RADAR [66, 68-70] | 20 different epi-transcriptome profiles based on various base resolution techniques | Multiple types of RNA modifications prediction |
|  | BertNDA | [71] | Nov 2023 | 8 layers | BERT-based | RNA sequence | miRNA-disease associations, lncRNA-disease associations, and miRNA-lncRNA associations [72-75] | 1000 positive pairs and 1000 negative pairs | ncRNA-Disease Association Prediction |
|  | LncCat | [76] | Feb 2023 | - | BERT-based | RNA sequence | lncRNAs and protein-coding transcripts of five species [10, 75, 77] | 22960 coding transcripts and 21081 lncRNAs of human.  20707 coding transcripts and 10707 lncRNAs of mouse.  15891 coding transcripts and 4382 lncRNAs of zebrafish.  4693 coding transcripts and 5377 lncRNAs of wheat  20584 coding transcripts and 3897 lncRNAs of chicken | Identify lncRNA |
|  | LSCPP-BERT | [78] | Dec 2023 | 4 identical layers and each layer is divided into two sublayers | BERT-based | RNA sequence | lncRNAs sequences from multispecies [79] | 593251 plant lncRNAs sequences | lncRNA-sORFs coding potential prediction |
|  | CodonBERT | [80] | Oct 2023 | 12 layers of bidirectional transformer encoders, Each transformer layer with 12 self-attention heads | BERT-based | RNA sequence | mRFP Expression dataset; [81] Fungal expression dataset; E. coli proteins dataset; mRNA stability dataset; Tc-Riboswitches dataset [82] | Experimental data for protein expression (2308 low expression proteins, 2067 medium expression proteins, and 1973 high expression proteins, respectively); | mRNA properties prediction |
|  |  |  |  |  |  |  | SARS-CoV-2 Vaccine degradation dataset | - | Vaccine expression prediction |
|  | RNA-TorsionBERT | [83] | Jun 2024 | 18 layers | BERT-based | RNA sequence | PDB structure and removed the structures from the nonredundant Training | 4,267 structures with sequences from 11 to 508 nucleotides | RNA 3D structure prediction |
|  | UNI-RNA | [84] | Jul 2025 | - | BERT-based | RNA sequence | non-coding RNA sequences from RNAcentral, nucleic acid data from NCBI’s database, and genomic data from repositories such as Genome Warehouse[85-87] | 37,149 RNA structures  7,600 held-out real human 5’UTRs  3 million distinct UTR sequences | RNA secondary structure prediction RNA distance map prediction mRNA 5′-UTR mean ribosome load prediction Alternative polyadenylation isoform prediction RNA splice site prediction ncRNA classification RNA modification prediction |
|  | UTR-LM | [88] | Apr 2024 | Six-layer transformer with 16 multi-head self-attention. | Transformer-based | RNA sequence | Unlabeled 5' UTR sequences from three sources: the Ensembl database | 214,349 unlabeled 5' UTR sequences | Mean ribosome loading prediction.  mRNA expression level and translation efficiency prediction  Internal ribosome entry site identification  Attention-based motif detection |
|  | 3UTRBERT | [89] | Aug 2024 | 12 identical Transformer components，each layer contained a multi-head self-attention module and a position-wise fully connected feed-forward layer | BERT-based | RNA sequence | 3’UTR of human mRNAtranscript  eCLIP datasets  RNA localization data | 108 573 unique mRNA transcripts  1000 samples RNA binding samples with1000 sequences  17 023 mRNAs | mRNA subcellular localization prediction |
|  | RNAErnie | [90] | May 2024 | 12-layer transformer and a hidden state dimension of 768 | BERT-based | RNA sequence | RNA transcript sequences (lncRNAs and protein-coding RNAs) with sequence-level categorical labels | Not explicitly specified | RNA sequence classification. |
|  |  |  |  |  |  | RNA sequence | RNA sequences with annotated secondary structures (base-pairing matrices). | Not explicitly specified | RNA secondary structure prediction |
|  |  |  |  |  |  | RNA sequence | Paired RNA sequence datasets (e.g., miRNA–mRNA, circRNA–lncRNA) with binary interaction labels. | Not explicitly specified | RNA–RNA interaction prediction. |
|  | HydraRNA | [91] | 2025 | hundreds of millions of parameters | BERT-based | RNA sequence | RNA  secondary structure from Singh et al. from bpRNA-1m database[51, 92] | Not explicitly specified | Prediction of RNA secondary structure |
|  |  |  |  |  |  | RNA sequence | 31 CLIP experiments on 19 RBPs including binding sites | Not explicitly specified | Prediction of RNA-binding protein binding site |
|  |  |  |  |  |  | RNA sequence | Human 5’UTRs, with length ranging from 25 to 100 nt[57] | 76,319 random 5’UTRs | Prediction of 5’UTR effect on ribosome loading |
|  |  |  |  |  |  | RNA sequence | Human 3’UTRs | Not explicitly specified | Prediction of 3’UTR effect on mRNA stability |
|  |  |  |  |  |  | RNA sequence | mRNA and protein abundance as well as  their turnover | Not explicitly specified | Prediction of mRNA translation and stability |
|  | RiNALMo | [93] | 2024 | - | BERT-based | RNA sequence | bpRNA database[92] | 13,419 non-redundant RNA secondary structures | Intra-family secondary structure prediction |
|  |  |  |  |  |  | RNA sequence | Benchmark and Rfam family-based datasets[54, 94] | 3,166 train and 430 test RNAs | Inter-family structure prediction |
|  |  |  |  |  |  | RNA sequence | Positive and negative subsets of splice-site sequences[95] | Not explicitly specified | RNA splice-site prediction |
|  |  |  |  |  |  |  | Rfam database[96] | 88 families, short ncRNAs | Predict family of ncRNA |
|  |  |  |  |  |  | RNA sequence | 5’ untranslated region (UTR) of mRNAs[57] | 7,600 UTR sequences with experimentally measured mean ribosome load | mRNA translation prediction |
|  | ERNIE-RNA | [97] | 2022 | 12-layer transformer encoder with hidden size of 768 | BERT-based | RNA sequence | Benchmarks dataset from bpRNA[92], ArchiveII[54], RIVAS[98], and RNA3DB[98] | Not explicitly specified | RNA secondary structure prediction |
|  |  |  |  |  |  | RNA sequence | Benchmark datasets from RNAcontact[99] and PETfold[100] | 301 RNA sequences with more than five annotated spatial contacts, split into 221 training and 80 testing sequences. | RNA contact map prediction |
|  |  |  |  |  |  | RNA sequence | Optimus 5-prime benchmark dataset[57] | 83,919 synthetic random 5’UTRs and 7,600 real human 5’UTRs | 5’UTR sequence mean ribosomal loading (MRL) prediction |
|  |  |  |  |  |  | RNA sequence | PrismNet benchmark dataset[101] | 17 RNA-binding proteins in the HeLa cell | RNA-protein binding prediction |
|  |  |  |  |  |  | RNA sequence | Benchmark framework[102] | 88 predefined ncRNA families | ncRNA family classification |
|  |  |  |  |  |  | RNA sequence | (i) the Spliceator benchmark comprising curated splice sites from over 100 eukaryotic species with four species held out for testing, and (ii) the BEACON benchmark based on the SpliceAI human dataset[95, 103] | 15,000 splice sites from human pre-mRNAs | Multi-species splice site prediction |
|  |  |  |  |  |  | RNA sequence | APARENT dataset[43, 103] | Over 3 million unique synthetic and natural human 3′ UTR sequences from 13 libraries | Alternative polyadenylation prediction |

References

1. Ji, Y., et al., *DNABERT: pre-trained Bidirectional Encoder Representations from Transformers model for DNA-language in genome.* Bioinformatics, 2021. **37**(15): p. 2112-2120.

2. Dreos, R., et al., *EPD and EPDnew, high-quality promoter resources in the next-generation sequencing era.* Nucleic acids research, 2013. **41**(D1): p. D157-D164.

3. Consortium, E.P., *An integrated encyclopedia of DNA elements in the human genome.* Nature, 2012. **489**(7414): p. 57.

4. Cunningham, F., et al., *Ensembl 2019.* Nucleic acids research, 2019. **47**(D1): p. D745-D751.

5. Sherry, S.T., et al., *dbSNP: the NCBI database of genetic variation.* Nucleic acids research, 2001. **29**(1): p. 308-311.

6. Zhou, Z., et al., *Dnabert-2: Efficient foundation model and benchmark for multi-species genome.* arXiv preprint arXiv:2306.15006, 2023.

7. Zeng, H., et al., *Convolutional neural network architectures for predicting DNA–protein binding.* Bioinformatics, 2016. **32**(12): p. i121-i127.

8. Chen, K., H. Zhao, and Y. Yang, *Capturing large genomic contexts for accurately predicting enhancer-promoter interactions.* Briefings in Bioinformatics, 2022. **23**(2): p. bbab577.

9. Dalla-Torre, H., et al., *The nucleotide transformer: Building and evaluating robust foundation models for human genomics.* bioRxiv, 2023: p. 2023.01. 11.523679.

10. Howe, K.L., et al., *Ensembl 2021.* Nucleic acids research, 2021. **49**(D1): p. D884-D891.

11. Bergström, A., et al., *Insights into human genetic variation and population history from 929 diverse genomes.* Science, 2020. **367**(6484): p. eaay5012.

12. Alonso-Blanco, C., et al., *1,135 genomes reveal the global pattern of polymorphism in Arabidopsis thaliana.* Cell, 2016. **166**(2): p. 481-491.

13. Zhang, D., et al., *DNAGPT: A Generalized Pretrained Tool for Multiple DNA Sequence Analysis Tasks.* bioRxiv, 2023: p. 2023.07. 11.548628.

14. Kalkatawi, M., et al., *DeepGSR: an optimized deep-learning structure for the recognition of genomic signals and regions.* Bioinformatics, 2019. **35**(7): p. 1125-1132.

15. Agarwal, V. and J. Shendure, *Predicting mRNA abundance directly from genomic sequence using deep convolutional neural networks.* Cell reports, 2020. **31**(7).

16. Sanabria, M., et al., *DNA language model GROVER learns sequence context in the human genome.* Nature Machine Intelligence, 2024. **6**(8): p. 911-923.

17. de Souza, N., *The ENCODE project.* Nature methods, 2012. **9**(11): p. 1046-1046.

18. Benegas, G., S.S. Batra, and Y.S. Song, *DNA language models are powerful predictors of genome-wide variant effects.* Proceedings of the National Academy of Sciences, 2023. **120**(44): p. e2311219120.

19. Le, N.Q.K., et al., *BERT-Promoter: An improved sequence-based predictor of DNA promoter using BERT pre-trained model and SHAP feature selection.* Computational Biology and Chemistry, 2022. **99**: p. 107732.

20. Gama-Castro, S., et al., *RegulonDB version 9.0: high-level integration of gene regulation, coexpression, motif clustering and beyond.* Nucleic acids research, 2016. **44**(D1): p. D133-D143.

21. Xiao, X., et al., *iPSW (2L)-PseKNC: A two-layer predictor for identifying promoters and their strength by hybrid features via pseudo K-tuple nucleotide composition.* Genomics, 2019. **111**(6): p. 1785-1793.

22. Luo, H., et al., *Improving language model of human genome for DNA–protein binding prediction based on task-specific pre-training.* Interdisciplinary Sciences: Computational Life Sciences, 2023. **15**(1): p. 32-43.

23. An, W., et al. *MoDNA: motif-oriented pre-training for DNA language model*. in *Proceedings of the 13th ACM International Conference on Bioinformatics, Computational Biology and Health Informatics*. 2022.

24. Luo, H., et al. *iEnhancer-BERT: A novel transfer learning architecture based on DNA-Language model for identifying enhancers and their strength*. in *International Conference on Intelligent Computing*. 2022. Springer.

25. Ernst, J., et al., *Mapping and analysis of chromatin state dynamics in nine human cell types.* Nature, 2011. **473**(7345): p. 43-49.

26. Tsukiyama, S., et al., *BERT6mA: prediction of DNA N6-methyladenine site using deep learning-based approaches.* Briefings in Bioinformatics, 2022. **23**(2): p. bbac053.

27. Xiao, C.-L., et al., *N6-methyladenine DNA modification in the human genome.* Molecular cell, 2018. **71**(2): p. 306-318. e7.

28. Ye, G., et al., *De novo genome assembly of the stress tolerant forest species Casuarina equisetifolia provides insight into secondary growth.* The Plant Journal, 2019. **97**(4): p. 779-794.

29. Ye, P., et al., *MethSMRT: an integrative database for DNA N6-methyladenine and N4-methylcytosine generated by single-molecular real-time sequencing.* Nucleic acids research, 2016: p. gkw950.

30. Liu, Z.-Y., et al., *MDR: an integrative DNA N6-methyladenine and N4-methylcytosine modification database for Rosaceae.* Horticulture research, 2019. **6**.

31. Wang, Y., et al., *N6-adenine DNA methylation is associated with the linker DNA of H2A. Z-containing well-positioned nucleosomes in Pol II-transcribed genes in Tetrahymena.* Nucleic acids research, 2017. **45**(20): p. 11594-11606.

32. Jin, J., et al., *iDNA-ABF: multi-scale deep biological language learning model for the interpretable prediction of DNA methylations.* Genome biology, 2022. **23**(1): p. 1-23.

33. Luo, Y., et al., *New developments on the Encyclopedia of DNA Elements (ENCODE) data portal.* Nucleic acids research, 2020. **48**(D1): p. D882-D889.

34. Zhang, J., et al., *An integrative ENCODE resource for cancer genomics.* Nature communications, 2020. **11**(1): p. 3696.

35. Lv, H., et al., *iDNA-MS: an integrated computational tool for detecting DNA modification sites in multiple genomes.* Iscience, 2020. **23**(4).

36. Yu, Y., et al., *iDNA-ABT: advanced deep learning model for detecting DNA methylation with adaptive features and transductive information maximization.* Bioinformatics, 2021. **37**(24): p. 4603-4610.

37. Zeng, W., A. Gautam, and D.H. Huson, *MuLan-Methyl-Multiple Transformer-based Language Models for Accurate DNA Methylation Prediction.* bioRxiv, 2023: p. 2023.01. 04.522704.

38. Fishman, V., et al., *GENA-LM: a family of open-source foundational DNA language models for long sequences.* Nucleic Acids Research, 2025. **53**(2): p. gkae1310.

39. Dreos, R., et al., *The Eukaryotic Promoter Database: expansion of EPDnew and new promoter analysis tools.* Nucleic acids research, 2015. **43**(D1): p. D92-D96.

40. de Almeida, B.P., et al., *DeepSTARR predicts enhancer activity from DNA sequence and enables the de novo design of enhancers.* bioRxiv, 2021: p. 2021.10. 05.463203.

41. Zhou, J. and O.G. Troyanskaya, *Predicting effects of noncoding variants with deep learning–based sequence model.* Nature methods, 2015. **12**(10): p. 931-934.

42. Jaganathan, K., et al., *Predicting splicing from primary sequence with deep learning.* Cell, 2019. **176**(3): p. 535-548. e24.

43. Bogard, N., et al., *A deep neural network for predicting and engineering alternative polyadenylation.* Cell, 2019. **178**(1): p. 91-106. e23.

44. Nguyen, E., et al., *Sequence modeling and design from molecular to genome scale with Evo.* Science, 2024. **386**(6723): p. eado9336.

45. Brixi, G., et al., *Genome modeling and design across all domains of life with Evo 2.* BioRxiv, 2025: p. 2025.02. 18.638918.

46. Hug, L.A., et al., *A new view of the tree of life.* Nature microbiology, 2016. **1**(5): p. 1-6.

47. Hartmann, S., et al., *Phytome: a platform for plant comparative genomics.* Nucleic Acids Research, 2006. **34**(suppl_1): p. D724-D730.

48. Notin, P., et al., *Proteingym: Large-scale benchmarks for protein fitness prediction and design.* Advances in Neural Information Processing Systems, 2023. **36**: p. 64331-64379.

49. Mi, H., et al., *Large-scale gene function analysis with the PANTHER classification system.* Nature protocols, 2013. **8**(8): p. 1551-1566.

50. Zhang, Y., et al., *Multiple sequence alignment-based RNA language model and its application to structural inference.* Nucleic Acids Research, 2024. **52**(1): p. e3-e3.

51. Singh, J., et al., *RNA secondary structure prediction using an ensemble of two-dimensional deep neural networks and transfer learning.* Nature communications, 2019. **10**(1): p. 5407.

52. Chen, J., et al., *Interpretable RNA foundation model from unannotated data for highly accurate RNA structure and function predictions.* bioRxiv, 2022: p. 2022.08. 06.503062.

53. Tan, Z., et al., *TurboFold II: RNA structural alignment and secondary structure prediction informed by multiple homologs.* Nucleic acids research, 2017. **45**(20): p. 11570-11581.

54. Sloma, M.F. and D.H. Mathews, *Exact calculation of loop formation probability identifies folding motifs in RNA secondary structures.* RNA, 2016. **22**(12): p. 1808-1818.

55. Wu, F., et al., *A new coronavirus associated with human respiratory disease in China.* Nature, 2020. **579**(7798): p. 265-269.

56. Sun, L., et al., *Predicting dynamic cellular protein–RNA interactions by deep learning using in vivo RNA structures.* Cell research, 2021. **31**(5): p. 495-516.

57. Sample, P.J., et al., *Human 5′ UTR design and variant effect prediction from a massively parallel translation assay.* Nature biotechnology, 2019. **37**(7): p. 803-809.

58. Akiyama, M. and Y. Sakakibara, *Informative RNA base embedding for RNA structural alignment and clustering by deep representation learning.* NAR genomics and bioinformatics, 2022. **4**(1): p. lqac012.

59. Chen, K., et al., *Self-supervised learning on millions of primary RNA sequences from 72 vertebrates improves sequence-based RNA splicing prediction.* Briefings in Bioinformatics, 2024. **25**(3): p. bbae163.

60. Haeussler, M., et al., *The UCSC genome browser database: 2019 update.* Nucleic acids research, 2019. **47**(D1): p. D853-D858.

61. Zhang, L., et al., *BERT-m7G: a transformer architecture based on BERT and stacking ensemble to identify RNA N7-Methylguanosine sites from sequence information.* Computational and Mathematical Methods in Medicine, 2021. **2021**.

62. Dai, C., et al., *Iterative feature representation algorithm to improve the predictive performance of N7-methylguanosine sites.* Briefings in Bioinformatics, 2021. **22**(4): p. bbaa278.

63. Li, Q., et al., *M6A-BERT-Stacking: A Tissue-Specific Predictor for Identifying RNA N6-Methyladenosine Sites Based on BERT and Stacking Strategy.* Symmetry, 2023. **15**(3): p. 731.

64. Dao, F.-Y., et al., *Computational identification of N6-methyladenosine sites in multiple tissues of mammals.* Computational and structural biotechnology journal, 2020. **18**: p. 1084-1091.

65. Soylu, N.N. and E. Sefer, *BERT2OME: Prediction of 2'-O-methylation Modifications from RNA Sequence by Transformer Architecture Based on BERT.* IEEE/ACM Transactions on Computational Biology and Bioinformatics, 2023.

66. Xuan, J.-J., et al., *RMBase v2. 0: deciphering the map of RNA modifications from epitranscriptome sequencing data.* Nucleic acids research, 2018. **46**(D1): p. D327-D334.

67. Liang, S., et al., *Rm-LR: A long-range-based deep learning model for predicting multiple types of RNA modifications.* Computers in Biology and Medicine, 2023. **164**: p. 107238.

68. Song, Z., et al., *Attention-based multi-label neural networks for integrated prediction and interpretation of twelve widely occurring RNA modifications.* Nature communications, 2021. **12**(1): p. 4011.

69. Barrett, T., et al., *NCBI GEO: archive for functional genomics data sets—update.* Nucleic acids research, 2012. **41**(D1): p. D991-D995.

70. Ramaswami, G. and J.B. Li, *RADAR: a rigorously annotated database of A-to-I RNA editing.* Nucleic acids research, 2014. **42**(D1): p. D109-D113.

71. Ning, Z., et al., *BertNDA: a Model Based on Graph-Bert and Multi-scale Information Fusion for ncRNA-disease Association Prediction.* bioRxiv, 2023: p. 2023.05. 18.541387.

72. Li, Y., et al., *HMDD v2. 0: a database for experimentally supported human microRNA and disease associations.* Nucleic acids research, 2014. **42**(D1): p. D1070-D1074.

73. Jiang, Q., et al., *miR2Disease: a manually curated database for microRNA deregulation in human disease.* Nucleic acids research, 2009. **37**(suppl_1): p. D98-D104.

74. Bao, Z., et al., *LncRNADisease 2.0: an updated database of long non-coding RNA-associated diseases.* Nucleic acids research, 2019. **47**(D1): p. D1034-D1037.

75. Gao, Y., et al., *Lnc2Cancer 3.0: an updated resource for experimentally supported lncRNA/circRNA cancer associations and web tools based on RNA-seq and scRNA-seq data.* Nucleic acids research, 2021. **49**(D1): p. D1251-D1258.

76. Feng, H., et al., *LncCat: An ORF attention model to identify LncRNA based on ensemble learning strategy and fused sequence information.* Computational and Structural Biotechnology Journal, 2023. **21**: p. 1433-1447.

77. O'Leary, N.A., et al., *Reference sequence (RefSeq) database at NCBI: current status, taxonomic expansion, and functional annotation.* Nucleic acids research, 2016. **44**(D1): p. D733-D745.

78. Xia, S., et al. *A multi-granularity information-enhanced pre-training method for predicting the coding potential of sORFs in plant lncRNAs*. in *2023 IEEE International Conference on Bioinformatics and Biomedicine (BIBM)*. 2023. IEEE.

79. Di Marsico, M., et al., *GreeNC 2.0: a comprehensive database of plant long non-coding RNAs.* Nucleic Acids Research, 2022. **50**(D1): p. D1442-D1447.

80. Babjac, A.N., Z. Lu, and S.J. Emrich. *CodonBERT: Using BERT for Sentiment Analysis to Better Predict Genes with Low Expression*. in *Proceedings of the 14th ACM International Conference on Bioinformatics, Computational Biology, and Health Informatics*. 2023.

81. Nieuwkoop, T., et al., *Revealing determinants of translation efficiency via whole-gene codon randomization and machine learning.* Nucleic acids research, 2023. **51**(5): p. 2363-2376.

82. Byrska-Bishop, M., et al., *High-coverage whole-genome sequencing of the expanded 1000 Genomes Project cohort including 602 trios.* Cell, 2022. **185**(18): p. 3426-3440. e19.

83. Bernard, C., et al., *RNA-TorsionBERT: leveraging language models for RNA 3D torsion angles prediction.* bioRxiv, 2024: p. 2024.06. 06.597803.

84. Wen, H., *BPS2025-A unified pre-trained RNA foundation model for dissecting and engineering RNA molecules.* Biophysical Journal, 2025. **124**(3): p. 323a.

85. *RNAcentral: a hub of information for non-coding RNA sequences.* Nucleic Acids Research, 2019. **47**(D1): p. D221-D229.

86. Sayers, E.W., et al., *Database resources of the national center for biotechnology information.* Nucleic acids research, 2022. **50**(D1): p. D20-D26.

87. Chen, M., et al., *Genome Warehouse: a public repository housing genome-scale data.* Genomics, Proteomics and Bioinformatics, 2021. **19**(4): p. 584-589.

88. Chu, Y., et al., *A 5′ UTR language model for decoding untranslated regions of mRNA and function predictions.* Nature Machine Intelligence, 2024. **6**(4): p. 449-460.

89. Yang, Y., et al., *Deciphering 3'UTR Mediated Gene Regulation Using Interpretable Deep Representation Learning.* Advanced Science, 2024. **11**(39): p. 2407013.

90. Wang, N., et al., *Multi-purpose RNA language modelling with motif-aware pretraining and type-guided fine-tuning.* Nature Machine Intelligence, 2024: p. 1-10.

91. Li, G., et al., *HydraRNA: a hybrid architecture based full-length RNA language model.* Genome Biology, 2025. **26**(1): p. 383.

92. Danaee, P., et al., *bpRNA: large-scale automated annotation and analysis of RNA secondary structure.* Nucleic acids research, 2018. **46**(11): p. 5381-5394.

93. Penić, R.J., et al., *Rinalmo: General-purpose rna language models can generalize well on structure prediction tasks.* Nature Communications, 2025. **16**(1): p. 5671.

94. Mathews, D.H., *How to benchmark RNA secondary structure prediction accuracy.* Methods, 2019. **162**: p. 60-67.

95. Scalzitti, N., et al., *Spliceator: multi-species splice site prediction using convolutional neural networks.* BMC bioinformatics, 2021. **22**(1): p. 561.

96. Kalvari, I., et al., *Rfam 13.0: shifting to a genome-centric resource for non-coding RNA families.* Nucleic acids research, 2018. **46**(D1): p. D335-D342.

97. Yin, W., et al., *ERNIE-RNA: an RNA language model with structure-enhanced representations.* Nature Communications, 2025. **16**(1): p. 10076.

98. Rivas, E., R. Lang, and S.R. Eddy, *A range of complex probabilistic models for RNA secondary structure prediction that includes the nearest-neighbor model and more.* Rna, 2012. **18**(2): p. 193-212.

99. Sun, S., et al., *RNA inter-nucleotide 3D closeness prediction by deep residual neural networks.* Bioinformatics, 2021. **37**(8): p. 1093-1098.

100. Seemann, S.E., J. Gorodkin, and R. Backofen, *Unifying evolutionary and thermodynamic information for RNA folding of multiple alignments.* Nucleic Acids Research, 2008. **36**(20): p. 6355-6362.

101. Xu, Y., et al., *PrismNet: predicting protein–RNA interaction using in vivo RNA structural information.* Nucleic Acids Research, 2023. **51**(W1): p. W468-W477.

102. Noviello, T.M.R., et al., *Deep learning predicts short non-coding RNA functions from only raw sequence data.* PLoS computational biology, 2020. **16**(11): p. e1008415.

103. Ren, Y., et al., *Beacon: Benchmark for comprehensive rna tasks and language models.* Advances in Neural Information Processing Systems, 2024. **37**: p. 92891-92921.
