# Supplementary material for: Advancing bioinformatics with language models: components, applications, and perspectives
Source: Brief Bioinform. 2026 Jul 10;27(4):bbag367. doi: 10.1093/bib/bbag367 (PMC13354062; doi:10.1093/bib/bbag367)
Supplement: Supplementary_material_bbag367 [file supplementary_material_bbag367.zip › Supplementary Table 2.docx]

**Supplementary Table 2. Detailed information of language models for proteomic tasks**

***"Transformer-based" refers exclusively to models using a full encoder-decoder architecture. Models that use only the encoder stack are categorized as "BERT-based", while models using only the decoder stack are categorized as "GPT-based".**

| **Application area** | **Models** | **Ref** | **Publication**  **time** | **Model configuration** | **Architecture** | **Datasets** | | | **Downstream tasks** |
| --- | --- | --- | --- | --- | --- | --- | --- | --- | --- |
|  |  |  |  |  |  | **Data type** | **Source** | **Size** |  |
| Protein Large Language Models | MSA Transformer | [1] | Jul 2021 | 100M parameters model with 12 layers, 768 embedding size, and 12 attention heads | BERT-based | MSAs | CAMEO [2] | 131 domains (129 evaluated) | Unsupervised contact prediction, supervised contact prediction, secondary structure prediction |
|  |  |  |  |  |  |  | CASP13-FM [3] | 31 free modeling domains (from 25 targets) |  |
|  |  |  |  |  |  |  | trRosetta training set [4] | 15,051 MSAs and structures (14,842 used) |  |
|  |  |  |  |  |  | Protein sequences | CB513 [5] | 513 protein sequences |  |
|  |  |  |  |  |  |  | Netsurf dataset [6] | 12,185 crystal structures obtained from the PDB [7] |  |
|  | UniRep | [8] | Dec 2019 | 18.2M parameters (a 1,900-hidden unit mLSTM) | LSTM-based | Protein sequences | UniRef50 [9] | 24M | Predicting stability of naturally occurring and de novo designed proteins, Prediction of functional effects of single mutations in diverse proteins |
|  |  |  |  |  |  |  | Mini protein dataset from [10] | 1,432 out of 5,570 test set and 1,416 out of 5,571 validation set |  |
|  |  |  |  |  |  |  | DMS dataset [11] | ~65,420 variants across the 8 proteins |  |
|  |  |  |  |  |  |  | avGFP dataset [12] | 32,400 variants derived from 27 homologs of avGFP (used) |  |
|  | TAPE | [13] | Dec 2019 | 38M parameters | (ResNet, BERT, LSTM)-based | Protein sequences | Netsurf dataset [6] | 12,185 protein sequences | Secondary structure (SS) prediction (structure prediction task), contact prediction (structure prediction task), remote homology detection (evolutionary understanding task), fluorescence landscape prediction (protein engineering task), stability landscape prediction (protein engineering task) |
|  |  |  |  |  |  |  | ProteinNet dataset [14] | ~332,283,871 protein sequences |  |
|  |  |  |  |  |  |  | DeepSF dataset [15] | Training set includes 16,712 proteins spanning 1,195 folds [16]; test datasets include 2,533 protein domains across 550 folds from SCOP 2.06 [16], a subset of SCOP 1.75 and the CASP dataset [17, 18] |  |
|  |  |  |  |  |  |  | avGFP dataset [12] | ~51,715 protein sequences |  |
|  |  |  |  |  |  |  | Dataset from [10] | ~ 46,800 protein sequences |  |
|  | ESM-1b | [19] | Dec 2020 | A model with ∼650M parameters (33 layers) | BERT-based | Protein sequences | SCOPe [20] | 15,297 protein sequences | Remote homology detection, prediction of secondary structure, long-range residue–residue contacts, mutational effect prediction, etc. |
|  |  |  |  |  |  |  | CB513 [5] | 513 protein sequences; |  |
|  |  |  |  |  |  |  | CASP13 [21] | 431 domains |  |
|  |  |  |  |  |  |  | Envision (DMS dataset) [11]  and DeepSequence [22] | Over 700,000 variant effect measurements from over 100 large-scale experimental mutagenesis datasets |  |
|  | ProtTrans | [23] | Aug 2021 | From millions to billions of parameters (224M-11B) | (Transformer-XL, XLNet, BERT, Albert, Electra, T5)-based | Protein sequences | CB513 [5] | 513 protein sequences | Per-residue secondary structure prediction，per-protein localization & membrane prediction |
|  |  |  |  |  |  |  | TS115 [5] | 115 protein sequences |  |
|  |  |  |  |  |  |  | CASP12 [24] | ~102 protein sequences |  |
|  |  |  |  |  |  |  | NEW364 [23] | 364 protein sequences |  |
|  |  |  |  |  |  |  | DeepLoc [25] | ~19,817 protein sequences |  |
|  |  |  |  |  |  |  | SCOPe 2.07 [26] | 14,323 protein sequences (non-redundant at PIDE < 40%) |  |
|  | SPRoBERTa | [27] | Sep 2022 | 12 transformer encoder layers, the embedding size is 768, the feed-forward hidden units are 3072 and the attention heads are 12 | BERT-based | Protein sequences | Netsurf dataset [6] | 12,185 protein sequences | Secondary structure prediction, contact prediction, remote homology prediction, protein function prediction or Gene Ontology (GO) term prediction |
|  |  |  |  |  |  |  | CB513 [5] | 513 protein sequences |  |
|  |  |  |  |  |  |  | CASP12 [24] | ~102 protein sequences |  |
|  |  |  |  |  |  |  | DeepSF dataset [15] | Consistent with the size used TAPE [13] |  |
|  |  |  |  |  |  |  | DeepFRI [28] | ~284,832 protein sequences with GO annotations |  |
|  | PromptProtein | [29] | Sep 2022 | 650M parameters with 33 layers and 20 attention heads. The embedding size is 1280 | BERT-based | Protein sequences | EC dataset from [28] | 19,199 protein sequences | Enzyme commission and Gene Ontology prediction, stability landscape prediction, fluorescence landscape prediction, thermostability landscape prediction, adeno-associated virus (AAV) landscape prediction, GB1 landscape prediction, antibody-antigen affinity prediction |
|  |  |  |  |  |  |  | GO dataset from [28] | 36,641 protein sequences |  |
|  |  |  |  |  |  |  | Protein engineering dataset from TAPE and FLIP [13, 30] | 68,965 protein sequences used in stability prediction, 54,025 used in fluorescence landscape prediction, 28,131 used in thermostability landscape prediction, 82,583 in AAV prediction, 8,733 in GB1 prediction |  |
|  | ProGen | [31] | Mar 2020 | 1.2B parameters. Sequence length is 512. The model has dimension d = 1028, inner dimension f = 512, 36 layers, and 8 heads per layer. Dropout with probability 0.1 follows the residual connections in each layer | GPT-based | Protein sequences, Conditioning tags | Uniparc [32], UniprotKB [33], SWISS-PROT [34], TrEMBL [35], Pfam [36], and NCBI taxonomic information [37] | 281M | Controllable protein generation and two case study: completing VEGFR2 kinase domain, zero-shot fitness selection for protein GB1 |
|  | Tranception | [38] | Jun 2022 | 700M parameters | GPT-based | Protein sequences | Protein Gym (a DMS dataset) [38] | ~1.8M protein sequences | Fitness prediction |
|  | ProtGPT2 | [39] | Jul 2022 | 738M parameters. 36 layers with a model dimensionality of 1280. | GPT-based | Protein sequences | UniRef50 [40] | 10,000 protein sequences | Sequence dataset generation, homology detection, disorder prediction |
|  |  |  |  |  |  |  | ProtGPT2 dataset [39] | Generated 10,000 protein sequences |  |
|  | ProteinBERT | [41] | Feb 2022 | 16M parameters. The model architecture consists of two almost parallel paths: one for local representations with d=128 and the other for global representations with d=512 | BERT-based | Protein sequences, Gene Ontology (GO) annotations | Secondary structure dataset from | 8,678 sequences (train) [13, 42] | (Secondary structure, disorder, Remote homology, fold classes, signal peptide, major PTMs, neuropeptide cleavage, fluorescence, stability) prediction |
|  |  |  |  |  |  |  | Disorder dataset from | 8,678 sequences (train) [42] |  |
|  |  |  |  |  |  |  | Remote homology dataset from | 12,312 sequences (train) [13, 43, 44] |  |
|  |  |  |  |  |  |  | Fold classes dataset from | 15,680 sequences (train) [43, 44] |  |
|  |  |  |  |  |  |  | Signal peptide dataset from | 16,606 sequences (train) [45] |  |
|  |  |  |  |  |  |  | Major PTMs dataset from | 43,356 sequences (train) [46] |  |
|  |  |  |  |  |  |  | Neuropeptide cleavage dataset from | 2,727 sequences (train) [47, 48] |  |
|  |  |  |  |  |  |  | Fluorescence dataset from | 21,446 sequences (train) [12, 13] |  |
|  |  |  |  |  |  |  | Stability dataset from | 53,679 sequences (train) [10] |  |
|  | ProtST | [49] | Jan 2023 | Depends on the parameters of the chosen language model | Multi-models-based (Protein Language Models and Biomedical Language Models) | Protein sequences, property descriptions | ProtDescribe [50, 51] | 553,052 aligned pairs of protein sequence and property description | Protein localization prediction, fitness landscape prediction, protein function annotation (totally 11 downstream tasks) |
|  | KeAP | [52] | Jan 2023 | Depends on the parameters of the chosen language model | Multi-cascade Bert-like network | Triplet in the format of (Protein, Relation, Attribute) | ProteinKG25 [53] | 5M with nearly 600k protein, 50k attribute terms, and 31 relation terms included | Amino acid contact prediction, protein homology detection, protein stability prediction, protein-protein interaction identification, protein-protein binding affinity prediction, and semantic similarity inference |
|  | CaLM | [54] | Jan 2024 | 86M parameters. 12 transformer layers contain 12 attention heads, with dimension 768. Similar to architectures of ESM family | BERT-based | Protein-coding DNA (cDNA) | cDNA dataset obtained from the European Nucleotide Archive with a timestamp of April 2022 | 9,858,385 cDNA sequences of seven model organisms | Melting point prediction, solubility prediction, subcellular localization prediction and function prediction |
|  |  |  |  |  |  |  | Melting temperature dataset [30, 55] | - |  |
|  |  |  |  |  |  |  | Subcellular localization dataset [30, 56] | - |  |
|  |  |  |  |  |  |  | Solubility dataset [57] | - |  |
|  |  |  |  |  |  |  | Gene ontology dataset [58] | - |  |
|  |  |  |  |  |  |  | Transcriptomics dataset [59] | - |  |
|  |  |  |  |  |  |  | Proteomics dataset [60] | - |  |
|  | PLMSearch | [61] | Mar 2024 | ESM-1b (650M parameters) [19] and ProtT5-XL-UniRef50 (3B parameters) [23] | BERT-based | Protein sequences | SCOPe40 [20, 62], New protein search test, Swiss-Prot [34], CATHS40 [63] | ~489,764 sequences for training | Homologous protein search |
|  | DHR | [64] | Jul 2024 | 2 ESM-1b (650M parameters per encoder) [19] | BERT-based | Protein sequences | UniRef [9],  SCOPe [20, 62] | ~2 M | Protein homolog detection |
|  | ESM3 | [65] | Jan 2025 | ESM3-small：1.4B parameters, 48 layers, embedding size d_model=  1536, d_head=64，context length = 2048;  ESM3- medium：7.7B parameters, 96 layers, embedding size d_model=  2560, d_head= 128，context length = 2048;  ESM3- large：98.5B parameters, 216 layers, embedding size d_model=  6144, d_head= 128，context length = 2048. | BERT-based, multimodal-based (track-tokenized) | Protein sequence, protein structure, derived structural labels, function conditioning, residue-level functional annotations | UniRef [9] | 83M sequences (70% clustered); 156M sequences (90% clustered); 54.6B tokens | Protein contact prediction, Single-sequence protein structure prediction, Protein structure confidence calibration, Protein function prediction, Zero-shot mutational effect / fitness prediction, Ligand-binding motif scaffolding, Secondary-structure–conditioned protein generation, etc. |
|  |  |  |  |  |  |  | MGnify [66] | 372M sequences (70% clustered); 621M sequences (90% clustered); 105.5B tokens |  |
|  |  |  |  |  |  |  | JGI [67] | 2029M sequences (70% clustered); 256B tokens |  |
|  |  |  |  |  |  |  | OAS [68] | 1192M sequences (95% clustered); 132B tokens |  |
|  |  |  |  |  |  |  | PDB [69] | 203K chains; 0.054B tokens |  |
|  |  |  |  |  |  |  | PDB Clustered [69] | 46K chains (70% clustered); 100K chains (100%); 0.027B tokens |  |
|  |  |  |  |  |  |  | AlphaFoldDB [70] | 36M structures (70% clustered); 69M structures (90% clustered); 40.5B tokens |  |
|  |  |  |  |  |  |  | ESMAtlas [71] | 87M structures (70% clustered); 179M structures (90% clustered); 23.5B tokens |  |
|  |  |  |  |  |  |  | AFDB inverse folded [65] | 111M unique samples; 33,300M unique tokens |  |
|  |  |  |  |  |  |  | ESMAtlas inverse folded [65] | 251M unique samples; 57,730M unique tokens |  |
|  |  |  |  |  |  |  | CAMEO test set [2] | 902 proteins |  |
|  |  |  |  |  |  |  | CASP14 [72] | 71 proteins |  |
|  |  |  |  |  |  |  | CASP15 [73] | 70 proteins |  |
|  |  |  |  |  |  |  | ProteinGym (DMS benchmark collection) [74] | 217 DMS datasets (28 viral; 189 non-viral/other) |  |
|  |  |  |  |  |  |  | BioLip ligand-binding motif set (tertiary coordination evaluation) [75] | 46 proteins with ligand-binding sites |  |
|  | ProTrek | [76] | Oct 2025 | ProTrek Large: sequence encoder 650M, structure encoder 150M, text/function encoder 130M parameters;  ProTrek Compact: sequence encoder 35M, structure encoder 35M, text/function encoder 130M parameters | BERT-based multimodal-based (ESM-2 + BERT (structure) + PubMedBERT” triple-encoder contrastive model) | Protein sequences, potein structure, protein function text / natural-language descriptions | SWISS-PROT [35] | 14 million high-precision protein–text pairs (curated) | Thermostability prediction, subcellular localization prediction, binary localization prediction, structure class prediction, structural similarity prediction, binding site detection (residue-level), metal ion binding prediction, fluorescence (variant effect) prediction, stability (variant effect) prediction, β-lactamase (variant effect / fitness) prediction, AAV (adeno-associated virus) variant effect prediction |
|  |  |  |  |  |  |  | TrEMBL50 [77] | 25 million “noisy” protein–text pairs, obtained by scoring/filtering a pool of 300 million pairs from TrEMBL50 (clustered at 50% sequence identity) |  |
|  |  |  |  |  |  |  | UniProt [9] | negative set: 100,000 randomly sampled proteins as unknown negatives (for retrieval evaluation). |  |
|  |  |  |  |  |  |  | OMG_prot50 [78] | 200 million proteins (used for the UDG mining experiment) |  |
|  |  |  |  |  |  |  | ProTrek web platform [76] | over 5 billion proteins, integrating seven databases: SWISS-PROT, UniRef50, Protein Data Bank [69], Open MetaGenomic (OMG), MGnify [66], global ocean microbiome protein catalog [79], National Center for Biotechnology Information [80] |  |
| Antibody  Large Language Models | MHCRoBERTa | [81] | Dec 2021 | Model with 12 multi-heads and 5 self-attention layers. | ROBERTa-based | Protein sequences | UniProtKB [82] | 565,254 protein sequences | Predicting the binding of peptide and major histocompatibility complex (MHC) |
|  |  |  |  |  |  |  | Immune Epitope Database (IEDB) [83] | MHCclass I transmembrane proteins containing HLA-A (1,777 sequences)，HLA-B (2,100 sequences) and HLA-C(1,931 sequences) |  |
|  | BERTMHC | [84] | Jun 2021 | The model has 12 layers with 12 self-attention heads in each layer | BERT-based | Protein sequences | The data from Kamilla Kjærgaard Jensen [85] | 2,413additional MHC–peptide pairs covering 47 MHC class II alleles. | Predicting precisely the binding and presentation of peptides to major histocompatibility complex (MHC) alleles |
|  |  |  |  |  |  |  | Immune Epitope Database (IEDB) [83] | 95,638 peptides |  |
|  | TCR-BERT | [86] | BioRxiv posted Nov 2021 | 12 stacked transformer blocks with 8 attention heads, utilizing a hidden representation dimensionality of 768 and featuring 12 transformer layers. | BERT-based | Protein sequences | Pan immune repertoire database (PIRD) [87] | 47,040 TRB sequences and 4,607 TRA sequences. | Antigen specificity classification |
|  |  |  |  |  |  |  | VDJdb [88] | 58,795 human TCRs and 3,353 mouse TCRs. |  |
|  |  |  |  |  |  |  | TCRdb [89] | 139,00,913 TRB sequences of unknown antigen binding affinity. |  |
|  | AntiFormer | [90] | July 2024 | 12 stacking layers | BERT-based | Protein sequences Gene expression | The OAS database[91] | 55 BCR-seq datasets containing 600 million sequences | Binding specificity prediction |
|  | SC-AIR-BERT | [92] | May 2023 | 6 standard transformer layers and each layer has 4 attention heads, the hidden representation dimensionality is 512 and the inter mediate representation dimensionality is 2048 | BERT-based | Protein sequences | VDJdb [88] | 23,358 unique paired TCRs | Binding specificity prediction |
|  |  |  |  |  |  |  | Immune Epitope Database (IEDB) [83] | 18,662 paired TCR sand 589 paired BCRs |  |
|  |  |  |  |  |  |  | huARdb [93] | 612,077 high confidence paired full-length α/β chains of TCR sequences |  |
|  |  |  |  |  |  |  | CoV-AbDab [94] | 1,105,906 paired antibody heavy/light chains |  |
|  | AbLang | [95] | Jun 2022 | Consists of three modules (Each of AbRep’s 12 transformer blocks has 12 attenuated heads, an inner hidden size of 3072 and a hidden size of 768. From AbRep, the rescodings (768 values for each residue) are obtained. AbHead follows the design of RoBERTa’s [96] head model, with a hidden size of 768) | BERT-based | Human antibody sequences | Observed Antibody Space (OAS) database [97] | Training sets of 14,126 724 heavy and 187,068 light sequences, and two evaluation sets of 100,000 heavy and 50,000 light sequences | Sequence specific predictions, residue specific predictions, amino acid predictions |
|  |  |  |  |  |  |  | Data from [98] | 10,000 naive and 10,000 memory B-cell sequences |  |
|  | AntiBERTa |  | Jul 2022 | 86M parameters. A 12-layer transformer model. Attention heads is 12, embedding dimension is 768, feedforward layer dimension is 3072 | BERT-based | Human antibody sequences | SAbDab [99] | Training/validation/test split of 720/90/90 | Trace the B cell origin of the antibody, quantify immunogenicity, predict the antibody’s binding site |
|  |  |  |  |  |  |  | BCR repertoire dataset [98] | - |  |
|  |  |  |  |  |  |  | TheraSAbDab [100] | 191 non-redundant therapeutic antibodies |  |
|  | EATLM | [101] | Jan 2023 | 86M parameters (12 layers, 12 heads, and 768 hidden states) | BERT-based | Human antibody sequences | Dataset from [102] | Training/validation/test split of 15,128/3,242/3,242 | Accurate antigen-binding prediction， paratope prediction， B cell analysis， antibody discovery |
|  |  |  |  |  |  |  | Paratope data from [103] | 1,662 CDR segments on 277 antibodies |  |
|  |  |  |  |  |  |  | Data from [104] | 88,094 sequences with 6 maturation stages |  |
|  |  |  |  |  |  |  | A subset of the OAS database [97] | Antibody sequences from 133 SARS-CoV-2 patients and 87 health persons |  |
|  | S^2^ALM | [105] | Aug 2025 | 650M trainable parameters; 33 encoder blocks, 20 self-attention heads per block, hidden size = 1280, Max sequence length = 1024 | BERT-based | Antibody sequences & structures; additional protein sequences & structures | UniRef50 [9] | 65 million protein sequences | Antigen binding capacity prediction, B cell maturation state classification, antibody paratope prediction, antigen–antibody binding affinity prediction, computational antibody CDR-H3 design (sequence infilling) |
|  |  |  |  |  |  |  | PDB [7] | 0.2 million experimentally determined protein 3D structures |  |
|  |  |  |  |  |  |  | AlphaFoldDB [106] | 10 million computationally predicted protein 3D structures (randomly sampled) |  |
|  |  |  |  |  |  |  | OAS [68] | 216,437,989 unique antibody sequences; and 10 million sequences randomly selected for training |  |
|  |  |  |  |  |  |  | SabDab [99] | 8850 antibody structures |  |
|  |  |  |  |  |  |  | ABodyBuilder2 [107] | 150K computationally predicted antibody structures |  |
|  |  |  |  |  |  |  | IgFold [108] | 1.4M computationally predicted antibody structures |  |
|  |  |  |  |  |  |  | HER2–trastuzumab antigen binding dataset [102, 109] | 21,612 unique antibody sequences, split 15,128 / 3,242 / 3,242 (train/val/test) |  |
|  |  |  |  |  |  |  | B cell maturation dataset [104, 109] | 88,094 antibody sequences, 6 maturation states |  |
|  |  |  |  |  |  |  | Paratope dataset from [109, 110] | 277 antibody sequences and 900 antibody sequences with token-wise paratope annotations |  |
|  |  |  |  |  |  |  | BioMap [111] | 1,706 antigen–antibody pairing data points with labeled binding affinity values |  |
|  |  |  |  |  |  |  | CoV-AbDab [94, 112] | coronavirus-binding antibodies/nanobodies (sequences & structures), split 2,282 / 291 / 291 (train/val/test) samples |  |

**References**

1. Rao, R.M., et al. *MSA transformer*. in *International Conference on Machine Learning*. 2021.

2. Haas, J., et al., *Continuous Automated Model EvaluatiOn (CAMEO) complementing the critical assessment of structure prediction in CASP12.* Proteins: Structure, Function, and Bioinformatics, 2018. **86**: p. 387-398.

3. Shrestha, R., et al., *Assessing the accuracy of contact predictions in CASP13.* Proteins: Structure, Function, and Bioinformatics, 2019. **87**(12): p. 1058-1068.

4. Yang, J., et al., *Improved protein structure prediction using predicted interresidue orientations.* Proceedings of the National Academy of Sciences, 2020. **117**(3): p. 1496-1503.

5. Cuff, J.A. and G.J. Barton, *Evaluation and improvement of multiple sequence methods for protein secondary structure prediction.* Proteins: Structure, Function, and Bioinformatics, 1999. **34**(4): p. 508-519.

6. Klausen, M.S., et al., *NetSurfP-2.0: Improved prediction of protein structural features by integrated deep learning.* Proteins: Structure, Function, and Bioinformatics, 2019. **87**(6): p. 520-527.

7. Berman, H.M., et al., *The protein data bank.* Nucleic acids research, 2000. **28**(1): p. 235-242.

8. Alley, E.C., et al., *Unified rational protein engineering with sequence-based deep representation learning.* Nature methods, 2019. **16**(12): p. 1315-1322.

9. Suzek, B.E., et al., *UniRef clusters: a comprehensive and scalable alternative for improving sequence similarity searches.* Bioinformatics, 2015. **31**(6): p. 926-932.

10. Rocklin, G.J., et al., *Global analysis of protein folding using massively parallel design, synthesis, and testing.* Science, 2017. **357**(6347): p. 168-175.

11. Gray, V.E., et al., *Quantitative missense variant effect prediction using large-scale mutagenesis data.* Cell systems, 2018. **6**(1): p. 116-124.

12. Sarkisyan, K.S., et al., *Local fitness landscape of the green fluorescent protein.* Nature, 2016. **533**(7603): p. 397-401.

13. Rao, R., et al., *Evaluating protein transfer learning with TAPE.* Advances in neural information processing systems, 2019. **32**.

14. AlQuraishi, M., *ProteinNet: a standardized data set for machine learning of protein structure.* BMC bioinformatics, 2019. **20**: p. 1-10.

15. Hou, J., B. Adhikari, and J. Cheng, *DeepSF: deep convolutional neural network for mapping protein sequences to folds.* Bioinformatics, 2018. **34**(8): p. 1295-1303.

16. Murzin, A.G., et al., *SCOP: a structural classification of proteins database for the investigation of sequences and structures.* Journal of molecular biology, 1995. **247**(4): p. 536-540.

17. Kinch, L.N., et al., *CASP9 target classification.* PROTEINS: structure, function, and bioinformatics, 2011. **79**(S10): p. 21-36.

18. Kinch, L.N., et al., *CASP 11 target classification.* Proteins: Structure, Function, and Bioinformatics, 2016. **84**: p. 20-33.

19. Rives, A., et al., *Biological structure and function emerge from scaling unsupervised learning to 250 million protein sequences.* Proceedings of the National Academy of Sciences, 2021. **118**(15): p. e2016239118.

20. Fox, N.K., S.E. Brenner, and J.-M. Chandonia, *SCOPe: Structural Classification of Proteins—extended, integrating SCOP and ASTRAL data and classification of new structures.* Nucleic acids research, 2014. **42**(D1): p. D304-D309.

21. Moult, J., et al., *Critical assessment of methods of protein structure prediction: Progress and new directions in round XI.* Proteins: Structure, Function, and Bioinformatics, 2016. **84**: p. 4-14.

22. Riesselman, A.J., J.B. Ingraham, and D.S. Marks, *Deep generative models of genetic variation capture the effects of mutations.* Nature methods, 2018. **15**(10): p. 816-822.

23. Elnaggar, A., et al., *ProtTrans: Towards Cracking the Language of Lifes Code Through Self-Supervised Deep Learning and High Performance Computing.* IEEE Transactions on Pattern Analysis and Machine Intelligence, 2021: p. 1-1.

24. Abriata, L.A., et al., *Assessment of hard target modeling in CASP12 reveals an emerging role of alignment-based contact prediction methods.* Proteins: Structure, Function, and Bioinformatics, 2018. **86**: p. 97-112.

25. Almagro Armenteros, J.J., et al., *DeepLoc: prediction of protein subcellular localization using deep learning.* Bioinformatics, 2017. **33**(21): p. 3387-3395.

26. Chandonia, J.-M., N.K. Fox, and S.E. Brenner, *SCOPe: classification of large macromolecular structures in the structural classification of proteins—extended database.* Nucleic acids research, 2019. **47**(D1): p. D475-D481.

27. Wu, L., et al., *SPRoBERTa: protein embedding learning with local fragment modeling.* Briefings in Bioinformatics, 2022. **23**(6): p. bbac401.

28. Gligorijevi, V., et al., *Structure-based protein function prediction using graph convolutional networks.* Nature communications, 2021. **12**(1): p. 3168.

29. Wang, Z., et al. *Multi-level Protein Structure Pre-training via Prompt Learning*. in *The Eleventh International Conference on Learning Representations*. 2022.

30. Dallago, C., et al., *FLIP: Benchmark tasks in fitness landscape inference for proteins.* bioRxiv, 2021: p. 2021-11.

31. Madani, A., et al., *Progen: Language modeling for protein generation.* arXiv preprint arXiv:2004.03497, 2020.

32. Leinonen, R., et al., *UniProt archive.* Bioinformatics, 2004. **20**(17): p. 3236-3237.

33. Bairoch, A., et al., *The universal protein resource (UniProt).* Nucleic acids research, 2005. **33**(suppl\_1): p. D154-D159.

34. Bairoch, A., et al., *Swiss-Prot: juggling between evolution and stability.* Briefings in bioinformatics, 2004. **5**(1): p. 39-55.

35. Boeckmann, B., et al., *The SWISS-PROT protein knowledgebase and its supplement TrEMBL in 2003.* Nucleic acids research, 2003. **31**(1): p. 365-370.

36. Bateman, A., et al., *The Pfam protein families database.* Nucleic acids research, 2004. **32**(suppl\_1): p. D138-D141.

37. Federhen, S., *The NCBI taxonomy database.* Nucleic acids research, 2012. **40**(D1): p. D136-D143.

38. Notin, P., et al. *Tranception: protein fitness prediction with autoregressive transformers and inference-time retrieval*. in *International Conference on Machine Learning*. 2022.

39. Ferruz, N., S. Schmidt, and B. Hcker, *ProtGPT2 is a deep unsupervised language model for protein design.* Nature communications, 2022. **13**(1): p. 4348.

40. *UniProt: the universal protein knowledgebase in 2021.* Nucleic acids research, 2021. **49**(D1): p. D480-D489.

41. Brandes, N., et al., *ProteinBERT: a universal deep-learning model of protein sequence and function.* Bioinformatics, 2022. **38**(8): p. 2102-2110.

42. Moult, J., et al., *Critical assessment of methods of protein structure prediction (CASP)—Round XII.* Proteins: Structure, Function, and Bioinformatics, 2018. **86**: p. 7-15.

43. Andreeva, A., et al., *SCOP2 prototype: a new approach to protein structure mining.* Nucleic acids research, 2014. **42**(D1): p. D310-D314.

44. Andreeva, A., et al., *The SCOP database in 2020: expanded classification of representative family and superfamily domains of known protein structures.* Nucleic acids research, 2020. **48**(D1): p. D376-D382.

45. Armenteros, J.J.A., et al., *SignalP 5.0 improves signal peptide predictions using deep neural networks.* Nature biotechnology, 2019. **37**: p. 420-423.

46. Hornbeck, P.V., et al., *PhosphoSitePlus, 2014: mutations, PTMs and recalibrations.* Nucleic acids research, 2015. **43**(D1): p. D512-D520.

47. Ofer, D. and M. Linial, *ProFET: Feature engineering captures high-level protein functions.* Bioinformatics, 2015. **31**(21): p. 3429-3436.

48. Brandes, N., D. Ofer, and M. Linial, *ASAP: a machine learning framework for local protein properties.* Database, 2016. **2016**: p. baw133.

49. Xu, M., et al. *Protst: Multi-modality learning of protein sequences and biomedical texts*. in *International Conference on Machine Learning*. 2023. PMLR.

50. Bairoch, A. and R. Apweiler, *The SWISS-PROT protein sequence database and its supplement TrEMBL in 2000.* Nucleic acids research, 2000. **28**(1): p. 45-48.

51. Xu, M., et al., *Protst: Multi-modality learning of protein sequences and biomedical texts.* arXiv preprint arXiv:2301.12040, 2023.

52. Zhou, H.-Y., et al., *Protein Representation Learning via Knowledge Enhanced Primary Structure Modeling.* bioRxiv, 2023: p. 2023-01.

53. Zhang, N., et al., *Ontoprotein: Protein pretraining with gene ontology embedding.* arXiv preprint arXiv:2201.11147, 2022.

54. Outeiral, C. and C.M. Deane, *Codon language embeddings provide strong signals for use in protein engineering.* Nature Machine Intelligence, 2024. **6**(2): p. 170-179.

55. Jarzab, A., et al., *Meltome atlas—thermal proteome stability across the tree of life.* Nature methods, 2020. **17**(5): p. 495-503.

56. Thumuluri, V., et al., *DeepLoc 2.0: multi-label subcellular localization prediction using protein language models.* Nucleic acids research, 2022. **50**(W1): p. W228-W234.

57. Sridharan, S., et al., *Proteome-wide solubility and thermal stability profiling reveals distinct regulatory roles for ATP.* Nature communications, 2019. **10**(1): p. 1155.

58. Unsal, S., et al., *Learning functional properties of proteins with language models.* Nature Machine Intelligence, 2022. **4**(3): p. 227-245.

59. Uhln, M., et al., *Tissue-based map of the human proteome.* Science, 2015. **347**(6220): p. 1260419.

60. Wang, M., et al., *PaxDb, a database of protein abundance averages across all three domains of life.* Molecular \& cellular proteomics, 2012. **11**(8): p. 492-500.

61. Liu, W., et al., *PLMSearch: Protein language model powers accurate and fast sequence search for remote homology.* Nature communications, 2024. **15**(1): p. 2775.

62. Chandonia, J.-M., et al., *SCOPe: improvements to the structural classification of proteins--extended database to facilitate variant interpretation and machine learning.* Nucleic acids research, 2022. **50**(D1): p. D553-D559.

63. Sillitoe, I., et al., *CATH: increased structural coverage of functional space.* Nucleic acids research, 2021. **49**(D1): p. D266-D273.

64. Hong, L., et al., *Fast, sensitive detection of protein homologs using deep dense retrieval.* Nature Biotechnology, 2024: p. 1-13.

65. Hayes, T., et al., *Simulating 500 million years of evolution with a language model.* Science, 2025. **387**(6736): p. 850-858.

66. Richardson, L., et al., *MGnify: the microbiome sequence data analysis resource in 2023.* Nucleic acids research, 2023. **51**(D1): p. D753-D759.

67. Grigoriev, I.V., et al., *The genome portal of the department of energy joint genome institute.* Nucleic acids research, 2012. **40**(D1): p. D26-D32.

68. Olsen, T.H., F. Boyles, and C.M. Deane, *Observed Antibody Space: A diverse database of cleaned, annotated, and translated unpaired and paired antibody sequences.* Protein Science, 2022. **31**(1): p. 141-146.

69. Burley, S.K., et al., *RCSB Protein Data Bank: biological macromolecular structures enabling research and education in fundamental biology, biomedicine, biotechnology and energy.* Nucleic acids research, 2019. **47**(D1): p. D464-D474.

70. Varadi, M., et al., *AlphaFold Protein Structure Database in 2024: providing structure coverage for over 214 million protein sequences.* Nucleic acids research, 2024. **52**(D1): p. D368-D375.

71. Lin, Z., et al., *Evolutionary-scale prediction of atomic-level protein structure with a language model.* Science, 2023. **379**(6637): p. 1123-1130.

72. Kryshtafovych, A., et al., *Critical assessment of methods of protein structure prediction (CASP)—Round XIV.* Proteins: Structure, Function, and Bioinformatics, 2021. **89**(12): p. 1607-1617.

73. Kryshtafovych, A., et al., *New prediction categories in CASP15.* Proteins: Structure, Function, and Bioinformatics, 2023. **91**(12): p. 1550-1557.

74. Notin, P., et al., *Proteingym: Large-scale benchmarks for protein fitness prediction and design.* Advances in Neural Information Processing Systems, 2023. **36**: p. 64331-64379.

75. Zhang, C., et al., *BioLiP2: an updated structure database for biologically relevant ligand--protein interactions.* Nucleic Acids Research, 2024. **52**(D1): p. D404-D412.

76. Su, J., et al., *A trimodal protein language model enables advanced protein searches.* Nature Biotechnology, 2025: p. 1-7.

77. Consortium, U., *UniProt: a hub for protein information.* Nucleic acids research, 2015. **43**(D1): p. D204-D212.

78. Cornman, A., et al., *The OMG dataset: An Open MetaGenomic corpus for mixed-modality genomic language modeling.* bioRxiv, 2024: p. 2024-08.

79. Chen, J., et al., *Global marine microbial diversity and its potential in bioprospecting.* Nature, 2024. **633**(8029): p. 371-379.

80. Pruitt, K.D., et al., *NCBI Reference Sequences (RefSeq): current status, new features and genome annotation policy.* Nucleic acids research, 2012. **40**(D1): p. D130-D135.

81. Wang, F., et al., *MHCRoBERTa: pan-specific peptide-MHC class I binding prediction through transfer learning with label-agnostic protein sequences.* Brief Bioinform, 2022. **23**(3).

82. Boutet, E., et al., *UniProtKB/Swiss-Prot: the manually annotated section of the UniProt KnowledgeBase*, in *Plant bioinformatics: methods and protocols*. 2007, Springer. p. 89-112.

83. Vita, R., et al., *The Immune Epitope Database (IEDB): 2018 update.* Nucleic Acids Res, 2019. **47**(D1): p. D339-D343.

84. Cheng, J., et al., *BERTMHC: improved MHC–peptide class II interaction prediction with transformer and multiple instance learning.* Bioinformatics, 2021. **37**(22): p. 4172-4179.

85. *Improved methods for predicting peptide binding affinity to MHCclass II molecules.* 2017.

86. Wu, K., et al., *TCR-BERT: learning the grammar of T-cell receptors for flexible antigenbinding analyses.* 2021.

87. Zhang, W., et al., *PIRD: Pan Immune Repertoire Database.* Bioinformatics, 2020. **36**(3): p. 897-903.

88. Bagaev, D.V., et al., *VDJdb in 2019: database extension, new analysis infrastructure and a T-cell receptor motif compendium.* Nucleic Acids Research, 2020. **48**(D1): p. D1057-D1062.

89. Chen, S.Y., et al., *TCRdb: a comprehensive database for T-cell receptor sequences with powerful search function.* Nucleic Acids Res, 2021. **49**(D1): p. D468-D474.

90. Wang, Q., et al., *AntiFormer: graph enhanced large language model for binding affinity prediction.* Briefings in Bioinformatics, 2024. **25**(5).

91. Olsen, T.H., F. Boyles, and C.M.J.P.S. Deane, *Observed Antibody Space: A diverse database of cleaned, annotated, and translated unpaired and paired antibody sequences.* 2022. **31**(1): p. 141-146.

92. Zhao, Y., et al., *SC-AIR-BERT: a pre-trained single-cell model for predicting the antigen-binding specificity of the adaptive immune receptor.* Brief Bioinform, 2023. **24**(4).

93. Wu, L., et al., *huARdb: human Antigen Receptor database for interactive clonotype-transcriptome analysis at the single-cell level.* Nucleic Acids Res, 2022. **50**(D1): p. D1244-D1254.

94. Raybould, M.I.J., et al., *CoV-AbDab: the coronavirus antibody database.* Bioinformatics, 2021. **37**(5): p. 734-735.

95. Olsen, T.H., I.H. Moal, and C.M. Deane, *AbLang: an antibody language model for completing antibody sequences.* Bioinformatics Advances, 2022. **2**(1): p. vbac046.

96. Liu, Y., et al., *Roberta: A robustly optimized bert pretraining approach.* arXiv preprint arXiv:1907.11692, 2019.

97. Kovaltsuk, A., et al., *Observed antibody space: a resource for data mining next-generation sequencing of antibody repertoires.* The Journal of Immunology, 2018. **201**(8): p. 2502-2509.

98. Ghraichy, M., et al., *Different B cell subpopulations show distinct patterns in their IgH repertoire metrics.* Elife, 2021. **10**: p. e73111.

99. Dunbar, J., et al., *SAbDab: the structural antibody database.* Nucleic acids research, 2014. **42**(D1): p. D1140-D1146.

100. Marks, C., et al., *Humanization of antibodies using a machine learning approach on large-scale repertoire data.* Bioinformatics, 2021. **37**(22): p. 4041-4047.

101. Wang, D., F. Ye, and H. Zhou, *On pre-trained language models for antibody.* bioRxiv, 2023: p. 2023-01.

102. Mason, D.M., et al., *Optimization of therapeutic antibodies by predicting antigen specificity from antibody sequence via deep learning.* Nature Biomedical Engineering, 2021. **5**(6): p. 600-612.

103. Liberis, E., et al., *Parapred: antibody paratope prediction using convolutional and recurrent neural networks.* Bioinformatics, 2018. **34**(17): p. 2944-2950.

104. Mroczek, E.S., et al., *Differences in the composition of the human antibody repertoire by B cell subsets in the blood.* Frontiers in immunology, 2014. **5**: p. 96.

105. Yin, M., et al., *S2alm: Sequence-structure pre-trained large language model for comprehensive antibody representation learning.* Research, 2025. **8**: p. 0721.

106. Varadi, M., et al., *AlphaFold Protein Structure Database: massively expanding the structural coverage of protein-sequence space with high-accuracy models.* Nucleic acids research, 2022. **50**(D1): p. D439-D444.

107. Abanades, B., et al., *ImmuneBuilder: Deep-Learning models for predicting the structures of immune proteins.* Communications Biology, 2023. **6**(1): p. 575.

108. Ruffolo, J.A., et al., *Fast, accurate antibody structure prediction from deep learning on massive set of natural antibodies.* Nature communications, 2023. **14**(1): p. 2389.

109. Wang, D., F. Ye, and H. Zhou, *On pre-trained language models for antibody.* arXiv preprint arXiv:2301.12112, 2023.

110. Leem, J., et al., *Deciphering the language of antibodies using self-supervised learning.* Patterns, 2022. **3**(7).

111. Chen, B., et al., *xTrimoPGLM: unified 100B-scale pre-trained transformer for deciphering the language of protein.* arXiv preprint arXiv:2401.06199, 2024.

112. Jin, W., et al., *Iterative refinement graph neural network for antibody sequence-structure co-design.* arXiv preprint arXiv:2110.04624, 2021.
