# Supplementary material for: Advancing bioinformatics with language models: components, applications, and perspectives
Source: Brief Bioinform. 2026 Jul 10;27(4):bbag367. doi: 10.1093/bib/bbag367 (PMC13354062; doi:10.1093/bib/bbag367)
Supplement: Supplementary_material_bbag367 [file supplementary_material_bbag367.zip › Supplementary Table 3.docx]

**Supplementary Table 3. Detailed information of language models for drug-discovery tasks**

***"Transformer-based" refers exclusively to models using a full encoder-decoder architecture. Models that use only the encoder stack are categorized as "BERT-based", while models using only the decoder stack are categorized as "GPT-based".**

| **Application area** | **Models** | **Ref** | **Publication**  **time** | **Model configuration** | **Architecture** | **Datasets** | | | **Downstream**  **tasks** |
| --- | --- | --- | --- | --- | --- | --- | --- | --- | --- |
|  |  |  |  |  |  | **Data type** | **Source** | **Size** |  |
| Drug discovery language models | SMILES-BERT | [1] | Sep 2019 | 6 Transformer encoder layers, the feed-forward hidden units are 1024 and the attention heads are 4 | BERT-based | SMILES | ZINC [2] | 18.69 M used, totally more than 35 M | LogP prediction, PM2 prediction, and molecular property prediction |
|  | ChemBERTa | [3] | arXiv posted Oct 2020 | Implementation of RoBERTa uses 12 attention heads and 6 layers, resulting in 72 distinct attention mechanisms. | BERT-based | SMILES | PubChem [4] | Curated a dataset of 77 M unique SMILES from PubChem, divided this dataset into subsets of 100 K, 250 K, 1 M, and 10 M. | Binary classification prediction of barrier permeability properties, binary classification of clinical trial toxicity, whether the compound inhibits HIV replication for binary classification between active and inactive |
|  | ChemBERTa-2 | [5] | arXiv posted Sep 2022 | Implementation of RoBERTa uses 12 attention heads and 6 layers, resulting in 72 distinct attention mechanisms. | BERT-based | SMILES | PubChem | Over a large corpus of 77 million SMILES strings | brain  penetrability, toxicity, solubility, and on-target inhibition |
|  | UniMoT | [6] | arXiv posted Aug 2024 | Based on a 7B-parameter Llama-2 language model with parameter-efficient fine-tuning (LoRA). | GPT-based | SMILES | MoleculeNet [7]  PubChem  Mol-Instructions [8] | MoleculeNet: 17 curated datasets comprising over 800 prediction tasks and approximately 700,000 compounds  PubChem: A large-scale molecular database containing over 77 million SMILES strings  Mol-Instructions: A biomolecular instruction dataset comprising approximately 706,000 instructions | Molecule Comprehension Tasks  Molecule Generation Tasks |
|  | MOLE-BERT | [9] | Apr 2023 | A 5-layer Graph Isomorphism Networks (GINs) whose hidden dimension is 300 | BERT-based, graph-based | Molecular graphs | ZINC15 [10] | 2 million molecules sampled from the dataset | Related tasks on 8 drug-discovery-related datasets including barrier permeability properties and clinical trial toxicity |
|  | MolGPT | [11] | Oct 2021 | 6M parameters. Each self-attention layer returns a vector of size 256. The hidden layer of the neural network outputs a vector of size 1024. The final layer of the fully connected neural network returns a vector of size 256. 8 decoder blocks | GPT-based | SMILES | MOSES [12] and GuacaMol [13] | 1.9 M molecules, 1.6 M molecules | Generating molecules |
|  | DrugGPT | [14] | bioRxiv posted June 2023 | DrugGPT is built upon a GPT-2–style autoregressive framework, with architectural details largely inherited from the base GPT-2 model. | GPT-based | SMILES | ZINC20 [15]  jglaser/  binding_affinity database [14] | 2 billion compounds  1,836,729 protein sequences | Chemical space exploration  SMILES-based molecule generation  Protein-conditioned ligand generation  Sequence-to-sequence, autoregressive |
|  | DTI-BERT | [16] | Jun 2022 | The proteins can be represented via 1024-D vectors (dimensionality of the features extracted by the ProtBert model). Drug molecular fingerprints are represented by 128-D vectors through semi decomposition process discrete wavelet transform (DWT). Secondly, the 1152-D vectors (a concatenation of protein sequence feature and drug feature) are fed into the feature extraction model to generate interaction information | BERT-based | Molecular fingerprints and protein sequence pairs | DrugBanks [17], BRENDA, SuperTarget, and KEGG BRITE [18] | 4,803 drug-target pairs in positive subsets 9,606 synthesized negative pairs | A pair belongs to an interactive drug-target pair or non-interactive drug-target pair |
|  | TransDTI | [19] | Jan 2022 | Consist of SMILES-BERT and fine-tuned large protein models. | BERT-based | Molecular SMILES and protein sequence pairs | KIBA [20] , gold-standard external data sets from DTI-MLCD [21] | 30, 474 compounds, 961 targets and 61, 624 interactions | A three-classification based on binding affinity |
|  | C2P2 | [22] | Jul 2022 | Consist of ChemBERTa and ESM model | BERT-based | Molecular SMILES and protein sequence pairs | STRING [23], STITCH [24], Davis [25], and PDBBind v2019 [26, 27] | Over 67.6 million proteins with over 20 billion protein–protein pairs, over 0.5 million chemicals with over 1.6 billion interactions, 30,056 interactions, 14,011 interactions | Binding affinity prediction |
|  | Hyeunseok Kang et al. | [28] | Aug 2022 | Consist of ChemBERTa and ProtBERT model | BERT-based | Molecular SMILES and protein sequence pairs | BIOSNAP [29], DAVIS [25] and BindingDB [30] | 27,482 interactions, 11,103 interactions, 32,601 interactions | Binding affinity prediction |
|  | DrugReAlign | [31] | Oct 2024 | - | GPT-based | natural-language representations of structural and interaction data | NR and GPCR [32] | enzymes (445 drugs, 664 targets, 2,926 interactions), ion channels (210 drugs, 204 targets, 1,476 interactions), GPCRs (223 drugs, 95 targets, 635 interactions), and nuclear receptors (54 drugs, 26 targets, 90 interactions) | Target-specific drug repurposing  Drug ranking |
|  | DrugCLIP | [33] | Jul 2024 | - | Dual-encoder representation model | SMILES  and disease codes | DrugBank  ZINC  ICD-10 Coding System [34] | 4,803 drug-target pairs in positive subsets 9,606 synthesized negative pairs | Drug Molecule Representation  Disease Code Representation |

**References**

1. Wang, S., et al. *Smiles-bert: large scale unsupervised pre-training for molecular property prediction*. in *Proceedings of the 10th ACM international conference on bioinformatics, computational biology and health informatics*. 2019.

2. Irwin, J.J., et al., *ZINC: a free tool to discover chemistry for biology.* Journal of chemical information and modeling, 2012. **52**(7): p. 1757-1768.

3. Chithrananda, S., G. Grand, and B. Ramsundar, *ChemBERTa: large-scale self-supervised pretraining for molecular property prediction.* arXiv preprint arXiv:2010.09885, 2020.

4. Kim, S., et al., *PubChem 2019 update: improved access to chemical data.* Nucleic acids research, 2019. **47**(D1): p. D1102-D1109.

5. *ChemBERTa-2: Towards Chemical Foundation Models.* 2022.

6. *UniMoT: Unified Molecule-Text Language Model with Discrete Token Representation.*

7. Wu, Z., et al., *MoleculeNet: a benchmark for molecular machine learning.* 2018. **9**(2): p. 513-530.

8. *MOL-INSTRUCTIONS: A LARGE-SCALE BIOMOLECULAR INSTRUCTION DATASET FOR LLMS.*

9. Xia, J., et al. *Mole-bert: Rethinking pre-training graph neural networks for molecules*. in *The Eleventh International Conference on Learning Representations*. 2022.

10. Sterling, T. and J.J. Irwin, *ZINC 15--ligand discovery for everyone.* Journal of chemical information and modeling, 2015. **55**(11): p. 2324-2337.

11. Bagal, V., et al., *MolGPT: molecular generation using a transformer-decoder model.* Journal of Chemical Information and Modeling, 2021. **62**(9): p. 2064-2076.

12. Polykovskiy, D., et al., *Molecular sets (MOSES): a benchmarking platform for molecular generation models.* Frontiers in pharmacology, 2020. **11**: p. 565644.

13. Brown, N., et al., *GuacaMol: benchmarking models for de novo molecular design.* Journal of chemical information and modeling, 2019. **59**(3): p. 1096-1108.

14. Li, Y., et al., *Druggpt: A gpt-based strategy for designing potential ligands targeting specific proteins.* bioRxiv, 2023: p. 2023.06. 29.543848.

15. Irwin, J.J., et al., *ZINC20-A Free Ultralarge-Scale Chemical Database for Ligand Discovery.* J Chem Inf Model, 2020. **60**(12): p. 6065-6073.

16. Zheng, J., X. Xiao, and W.-R. Qiu, *DTI-BERT: identifying drug-target interactions in cellular networking based on BERT and deep learning method.* Frontiers in Genetics, 2022. **13**: p. 859188.

17. Wishart, D.S., et al., *DrugBank 5.0: a major update to the DrugBank database for 2018.* Nucleic acids research, 2018. **46**(D1): p. D1074-D1082.

18. Hu, J., et al., *GPCR--drug interactions prediction using random forest with drug-association-matrix-based post-processing procedure.* Computational biology and chemistry, 2016. **60**: p. 59-71.

19. Kalakoti, Y., S. Yadav, and D. Sundar, *TransDTI: transformer-based language models for estimating DTIs and building a drug recommendation workflow.* ACS omega, 2022. **7**(3): p. 2706-2717.

20. Tang, J., et al., *Making sense of large-scale kinase inhibitor bioactivity data sets: a comparative and integrative analysis.* Journal of Chemical Information and Modeling, 2014. **54**(3): p. 735-743.

21. Chu, Y., et al., *DTI-MLCD: predicting drug-target interactions using multi-label learning with community detection method.* Briefings in bioinformatics, 2021. **22**(3): p. bbaa205.

22. Nguyen, T.M., T. Nguyen, and T. Tran, *Mitigating cold-start problems in drug-target affinity prediction with interaction knowledge transferring.* Briefings in Bioinformatics, 2022. **23**(4): p. bbac269.

23. Szklarczyk, D., et al., *The STRING database in 2021: customizable protein--protein networks, and functional characterization of user-uploaded gene/measurement sets.* Nucleic acids research, 2021. **49**(D1): p. D605-D612.

24. Kuhn, M., et al., *STITCH: interaction networks of chemicals and proteins.* Nucleic acids research, 2007. **36**(suppl\_1): p. D684-D688.

25. Davis, M.I., et al., *Comprehensive analysis of kinase inhibitor selectivity.* Nature Biotechnology, 2011. **29**(11): p. 1046-1051.

26. Wang, R., et al., *The PDBbind database: Collection of binding affinities for protein- ligand complexes with known three-dimensional structures.* Journal of medicinal chemistry, 2004. **47**(12): p. 2977-2980.

27. Wang, R., et al., *The PDBbind database: methodologies and updates.* Journal of medicinal chemistry, 2005. **48**(12): p. 4111-4119.

28. Kang, H., et al., *Fine-tuning of bert model to accurately predict drug--target interactions.* Pharmaceutics, 2022. **14**(8): p. 1710.

29. Zitnik, M., R. Sosic, and J. Leskovec, *BioSNAP Datasets: Stanford biomedical network dataset collection.* Note: <http://snap>. stanford. edu/biodata Cited by, 2018. **5**(1).

30. Liu, T., et al., *BindingDB: a web-accessible database of experimentally determined protein--ligand binding affinities.* Nucleic acids research, 2007. **35**(suppl\_1): p. D198-D201.

31. Wei, J., et al., *DrugReAlign: a multisource prompt framework for drug repurposing based on large language models.* BMC Biol, 2024. **22**(1): p. 226.

32. Yamanishi, Y., et al., *Prediction of drug-target interaction networks from the integration of chemical and genomic spaces.* Bioinformatics, 2008. **24**(13): p. i232-40.

33. Fu, T., et al., *DrugCLIP: Contrastive Drug-Disease Interaction For Drug*, in *Proceedings of the 10th ACM International Conference on Bioinformatics, Computational Biology and Health Informatics*. 2019. p. 542-542.

34. Anker, S.D., J.E. Morley, and S. von Haehling, *Welcome to the ICD-10 code for sarcopenia.* J Cachexia Sarcopenia Muscle, 2016. **7**(5): p. 512-514.
