# Supplementary material for: Advancing bioinformatics with language models: components, applications, and perspectives
Source: Brief Bioinform. 2026 Jul 10;27(4):bbag367. doi: 10.1093/bib/bbag367 (PMC13354062; doi:10.1093/bib/bbag367)
Supplement: Supplementary_material_bbag367 [file supplementary_material_bbag367.zip › Supplementary Table 4.docx]

**Supplementary Table 4. Detailed information of language models for single-cell tasks**

***"Transformer-based" refers exclusively to models using a full encoder-decoder architecture. Models that use only the encoder stack are categorized as "BERT-based", while models using only the decoder stack are categorized as "GPT-based".**

| **Application area** | **Models** | **Ref** | **Publication time** | **Model configuration** | **Architecture** | **Fine-tuning datasets** | | | **Downstream tasks** |
| --- | --- | --- | --- | --- | --- | --- | --- | --- | --- |
|  |  |  |  |  |  | **Data type** | **Source** | **Size** |  |
| Single-cell large language models | scBERT | [1] | Sep 2022 | six Performer encoder layers and ten heads for each layer, 200 dimensions of gene embedding using gene2vec | BERT-based | scRNA-seq | The Panglao dataset [2] | 209 human single-cell datasets comprising 74 tissues with 1,126,580 cells | Cell type annotation, novel cell type discovery, robustness to batch effects and model interpretability |
|  |  |  |  |  |  |  | Zheng68k dataset [3] | 68,450 cells |  |
|  |  |  |  |  |  |  | Pancreas datasets [4-7] | - |  |
|  |  |  |  |  |  |  | MacParland dataset [8] | 8,444 cells |  |
|  |  |  |  |  |  |  | Heart datasets [9, 10] | 451,513 cells for pretraining and the 287,269 cells for benchmarking |  |
|  |  |  |  |  |  |  | Lung dataset [11] | 39,778 cells |  |
|  |  |  |  |  |  |  | Human Cell Atlas dataset [12] | 84,363 cells from 27 cell types among 15 major organs |  |
|  | scGPT | [13] | Feb 2024 | 12 stacked transformer blocks with 8 attention heads, 512 embedding sizes of the pre-trained foundation model, 512 hidden sizes of the fully connected layer | GPT-based | scRNA-seq | CELLxGENE scRNA-seq human PBMC Collection [14] | 33 million human PBMC scRNA-seq samples | Gene network inference |
|  |  |  |  |  |  |  | PBMC 10K dataset [15] | Two scRNA-seq data of 7,982 cells and 4,008 cells. | Multi-batch integration |
|  |  |  |  |  |  |  | Immune Human dataset [16] | 33,506 cells |  |
|  |  |  |  |  |  |  | hPancreas dataset [4-7, 17, 18] | 10,600 cells in the reference set and 4,218 cells in the query set | Cell type annotation |
|  |  |  |  |  |  |  | Adamson dataset [19] | 87 unique one-gene perturbations, each replicated in around 100 cells | Genetic perturbation prediction |
|  |  |  |  |  |  |  | Norman dataset [20] | 131 two-gene perturbations and 105 one-gene perturbations. Each perturbation is replicated in around 300-700 cells. |  |
|  |  |  |  |  |  | Single-cell multi-omics | 10X Multiome PBMC [21] | 9,631 cells | Multi-omic integration |
|  |  |  |  |  |  |  | ASAP PBMC [22] | Four datasets each contain 5,023, 3,666, 3,517, and 4,849 cells respectively |  |
|  | CIForm | [23] | July 2023 | 64 attention heads | BERT-based | scRNA-seq | Pancreas datasets [4-7] | - | Cell type annotation |
|  |  |  |  |  |  |  | Immune datasets [24-26] | - |  |
|  |  |  |  |  |  |  | Brain datasets [27-29] | - |  |
|  |  |  |  |  |  |  | Tabula Muris dataset [30] | Nearly 100000 cells from 20 organs and tissues |  |
|  |  |  |  |  |  |  | Zheng68k dataset [3] | 68,450 cells |  |
|  |  |  |  |  |  |  | ZhangTdataset [31] | 8,530 cells from 20 subtypes |  |
|  |  |  |  |  |  |  | Allen mouse brain dataset [32] | 12832cells |  |
|  | TOSICA | [17] | Jan 2023 | - | BERT-based | scRNA-seq | human pancreas (hPancreas) [4-7] | 10,600 cells for training and 4,218 for testing | Cell type annotation, new cell type discovery and batch correction |
|  |  |  |  |  |  |  | human bone (hBone, GSE152805) [33] | 14,615 cells for training and 11,525 for testing |  |
|  |  |  |  |  |  |  | human artery (hArtery, GSE159677) [34] | 10,960 cells for training and 35,399 for testing |  |
|  |  |  |  |  |  |  | mouse brain (mBrain) [28-30, 35] | 48,801 cells for training and 7,394 for testing |  |
|  |  |  |  |  |  |  | mouse pancreas (mPancreas, GSE132188) [36] | 25,465 cells for training and 10,886 for testing |  |
|  |  |  |  |  |  |  | mouse  atlas (mAtlas, GSE132042) [37] | 78,672 cells for training and 277,541 for testing |  |
|  | scTransSort | [38] | Mar 2023 | 12 layers of transformer | BERT-based | scRNA-seq | human cell atlas dataset | 295,805 cells from 35 tissues | Cell type annotation |
|  |  |  |  |  |  |  | mouse cell atlas dataset | 105,148 cells from 26 tissues and 103,148 cells from 26 tissues |  |
|  |  |  |  |  |  |  | data processed by Shao X et al. [39] | - |  |
|  | TransCluster | [40] | Oct 2022 | 5 attention heads | improved Transformer model | scRNA-seq | Human training data [41] | - | Cell type Identification |
|  |  |  |  |  |  |  | The Shao dataset [39] | - |  |
|  |  |  |  |  |  |  | The Baron dataset [4] | - |  |
|  | Geneformer | [42] | May 2023 | six transformer encoder units, input size of 2,048, 256 embedding dimensions, four attention heads per layer and feed forward size of 512 | BERT-based | scRNA-seq | iPSC differentiation data | Assayed in parallel on the Drop-seq (single cell) or DroNc-seq (single nucleus) platform | Batch effect removal, cell type annotation |
|  |  |  |  |  |  |  | Huggingface Dataset [43] | a largescale pretraining corpus, Genecorpus-30M, comprising 29.9 million human single-cell transcriptomes | discover key network regulators and candidate therapeutic targets |
|  | tGPT | [44] | May 2023 | 8 transformer decoder blocks with 1024 hidden units and 16 attention heads | GPT-based | scRNA-seq | Human Cell Atlas Census of Immune Cells (HCA) [45] | 282,588 Bone marrow cells from 64 healthy donors in Human Cell Atlas (HCA) project | single-cell clustering, inference of developmental lineage |
|  |  |  |  |  |  |  | Human cell Landscape (HCL) [41] | 586,135 human cells |  |
|  |  |  |  |  |  |  | Tabula Mursi dataset [30] | 54,865 cells |  |
|  |  |  |  |  |  |  | Macaque Retina dataset [46] | 124,965 cells |  |
|  |  |  |  |  |  | Bulk RNA-seq | The Cancer Genome Atlas (TCGA) [47] | 9,318 bulk samples | interrogation of feature representation of bulk tissues in relation to genomic alterations, prognosis and treatment response of immunotherapy |
|  |  |  |  |  |  |  | Genotype-Tissue Expression Project (GTEx) [48] | 11,688 bulk samples |  |
|  | DeepMAPS | [49] | Feb 2023 | - | BERT-based,  graph transformer | scRNA-seq | Multiple scRNA-seq data [4, 16] | Three scRNA-seq datasets with 20,125 cells, 14,878 cells and 16,382 cells | Cell clustering, infer cell-type-specific biological networks from scMulti-omics data |
|  |  |  |  |  |  | Single-cell multi-omics | CITE-seq data [50] | Three CITE-seq datasets with 25,171 cells, 32,029 cells and 16,750 cells |  |
|  |  |  |  |  |  |  | scRNA-ATAC-seq data | Four scRNA-ATAC-seq datasets with 3,009 cells, 11,898 cells, 3,233 cells and 10,970 cells |  |
|  | scMVP | [51] | Jan 2022 | 8 self-attention heads and each head takes 16-dimension feature | Transformer-based | Paired scRNA-seq and scATAC-seq data | sci-CAR cell line dataset [52] | 293T cell line, 3T3 cell line, 293T/3T3 cell mixture, and A549 cell line treated with dexamethasone (DEX) for 0 h, 1 h, and 3 h | dimensionality reduction, cell clustering, and developmental trajectory inference and generate separate imputations for differential analysis and cis-regulatory element identification |
|  |  |  |  |  |  |  | Paired-seq cell line dataset [53] | derived from HEK293, HepG2, and their cell  line mixture |  |
|  |  |  |  |  |  |  | SNARE-seq cell line dataset [54] | 5,081 cells |  |
|  |  |  |  |  |  |  | SHARE-seq [55] | 67,418 cells |  |
|  | scTranslator | [56] | Nov 2025 | 8-headed attention mechanism in each sub-layer, 117  million parameters. | Transformer-based | Bulk datasets | The Cancer Genome Atlas (TCGA) data [57-60] | 31 cancer types and 18,227 samples in total | translate single-cell transcriptome to proteome, predict protein abundance |
|  |  |  |  |  |  |  | The data from Clinical Proteomic Tumor Analysis Consortium (CPTAC) [61-67] |  |  |
|  |  |  |  |  |  |  | The dataset from Broad Institute [68, 69] |  |  |
|  |  |  |  |  |  |  | The dataset from Memorial Sloan Kettering Cancer Center (MSKCC) [70] |  |  |
|  |  |  |  |  |  | Single-cell datasets | The Seurat v4 PBMCs dataset [71] | 161,764 human peripheral blood mononuclear cells |  |
|  |  |  |  |  |  |  | The REAP-seq PBMCs dataset [72] | 4,330 PBMCs with simultaneous measurements of 44 proteins and 21,005 transcriptome genes |  |
|  |  |  |  |  |  |  | The CITE-seq CBMCs dataset [73] | simultaneous measurements of 13 cellular proteins and 16,508 transcriptome genes. This dataset includes 8,005 cord blood mononuclear cells (CBMCs) |  |
|  |  |  |  |  |  |  | The single-cell pan-cancer dataset [74] | 65,698 myeloid cells with only single-cell transcriptome data, involving 15,844 genes |  |
|  | scFoundation | [75] | June 2024 | 100 million parameters | BERT-based | Single-cell datasets | Baron dataset [4] | - | gene expression enhancement, tissue drug response prediction, cell clustering, single-cell drug response classification, and single-cell perturbation prediction |
|  |  |  |  |  |  |  | Zheng68K dataset [3] | 68,450 cells |  |
|  |  |  |  |  |  |  | Cancer drug response dataset [76] | - |  |
|  |  |  |  |  |  |  | Single cell drug response classification dataset | - |  |
|  |  |  |  |  |  |  | Perturbation dataset [77] | - |  |
|  | scMoFormer | [78] | Oct 2023 | Graph transformer | Transformer-based | Single-cell multi-omics | Joint measurements of gene expression and surface protein levels datasets from the NeurIPS multimodal single-cell integration competition of the year 2021 [25] and 2022 | - | use gene expression (RNA) to predict surface protein  level, protein levels to gene expression, gene expression to chromatin accessibility and chromatin accessibility to gene expression |
|  | GeneCompass | [79] | Sep 2024 | 12-layer transformer framework, 100 million parameters | BERT-based | single-cell transcriptomes | CHIP-Atlas related to PBMC cells on GSE43036 [80] | - | Gene embedding analysis, Gene expression profiling prediction |
|  |  |  |  |  |  |  | human multiple sclerosis (hMS), lung (hLung) and liver (hLiver) datasets, and mouse brain (mBrain), lung (mLung) and pancreas (mPancreas) datasets. | - | cell type annotation |
|  |  |  |  |  |  |  | Immune Human [16] | 33,506 cells | GRN inference |
|  |  |  |  |  |  |  | dataset provided by Srivatsan et al. [81] | - | Drug dose response prediction |
|  |  |  |  |  |  |  | predefined dosage-sensitive and nonsensitive gene datasets [42] | - | Gene dosage sensitivity predictions. |
|  |  |  |  |  |  |  | Norman dataset [20] | 131 two-gene perturbations and 105 one-gene perturbations. Each perturbation is replicated in around 300-700 cells. | In silico perturbation |
|  |  |  |  |  |  |  | mouse embryonic stem cells (ESCs) [82] | - | In silico quantitative perturbation |
|  | scMulan | [83] | BioRxiv posted Jan 2024 | 24-layer transformer, 368 million parameters | GPT-based | single-cell transcriptomic data | AHCA_BoneMarrow [12] | 3,000 bone marrow cells from a single adult donor | zero-shot cell type annotation |
|  |  |  |  |  |  |  | Simonson2023 [84] | 60,345 cells from 8 human left ventricle samples |  |
|  |  |  |  |  |  |  | Suo2022 [85] | 140,000 liver cells from 14 fetal donors at various developmental stages |  |
|  |  |  |  |  |  |  | Intestine_HCL_55k dataset [41] | 55,214 intestinal cells across 24 cell types |  |
|  |  |  |  |  |  |  | Immune cell dataset [86] | 274,346 cells spanning 18 batches | batch integration |
|  |  |  |  |  |  |  | Lung dataset [16] | 32,472 cells from 16 donors |  |
|  |  |  |  |  |  |  | six organs within the hECA-10M dataset [83] | 3,000 conditions | conditional cell  generation |
|  | UCE | [87] | BioRxiv posted Nov 2023 | a 33-layer model consisting of over 650 million parameters | BERT-based | single-cell gene expression dataset | Tabula Sapiens v2 dataset | human data from 581,430 cells, 27 tissues, batches and 162 unique cell types | zero-shot embedding of new datasets |
|  |  |  |  |  |  |  | a dataset of green monkey lymph node and lung cells [88] | 17 cell types | Cell type embedding for new species |
|  |  |  |  |  |  |  | naked mole rat spleen and circulating immune cells [89] | 24 cell types |  |
|  |  |  |  |  |  |  | two distinct chicken datasets (chick retina [90] and developing chick heart [91]) | 15 cell types in chicken heart dataset |  |
|  |  |  |  |  |  |  | mouse renal cells [92] |  | decode the function of newly discovered cell types |
|  | CellLM | [93] | arXiv posted June 2023 | Performer model, 10 layers, 16 attention heads, over 50 million parameters | BERT-based | scRNA-seq data | Zheng68k dataset [3] | 68,450 human peripheral blood mononuclear cells (PBMCs) with 11 highly related cell types | Cell type annotation |
|  |  |  |  |  |  |  | The pancreas Baron dataset [4] | 8,562 cells categorized into 13 different cell types |  |
|  |  |  |  |  |  |  | Human lung cancer cells (GSE149383) [94] | 2,739 cells | Single-cell drug sensitivity prediction |
|  |  |  |  |  |  |  | human oral squamous cancer cells (GSE117872) [95-97] | 1,302 cells |  |
|  |  |  |  |  |  | Single-omics cell line data | cell lines integrating from CCLE [98] and GDSC [99] | 555 cell lines and 223 drugs | Single-omics cell line drug sensitivity prediction |
|  | scCLIP | [100] | Oct 2023 | Vanilla transformers | transformer-based encoders | Paired scRNA and scATAC-seq data | Fetal Atlas [101, 102] | 377*,* 134 cells | integration of multi-modal single-cell sequencing data |
|  |  |  |  |  |  |  | Brain [103] | - |  |
|  | iSEEEK | [104] | Jan 2022 | 8 transformer layers each with 576 hidden units and 8 attention heads | BERT-based | single-cell expression data | PBMCs [105] | 43,073 cells | Cell clustering |
|  |  |  |  |  |  |  | Human Cell Atlas Census of Immune Cells (HCA) [45] | 282,588 Bone marrow cells from 64 healthy donors in Human Cell Atlas (HCA) project |  |
|  |  |  |  |  |  |  | Tabula Muris dataset [30] | Nearly 100000 cells from 20 organs and tissues |  |
|  |  |  |  |  |  |  | Zheng68k dataset [3] | 68,579 cells |  |
|  |  |  |  |  |  |  | the dataset of FACS-sorted CD4/8+ T cells [31, 106, 107] | 12,670 CD4+ and 9,012 CD8+ T cells | Identify gene–gene interaction networks |
|  | CellPLM | [108] | BioRxiv posted Oct 2023 | over 80 million parameters | BERT-based | scRNA-seq and spatially-resolved transcriptomic (SRT) data | dataset from Li et al. [109] | 48*,* 082 cells | zero-shot clustering |
|  |  |  |  |  |  |  | PBMC 5K and Jurkat from 10x Genomics | 33,538 cells in PBMC 5K and 32,738 cells in Jurkat | scRNA-seq denoising |
|  |  |  |  |  |  |  | two spatial transcriptomic datasets at single-cell resolution, i.e., Lung2 and Liver2 | 836,739 cells in Lung2 and 598,141 cells in Liver2 | spatial transcriptomic imputation |
|  |  |  |  |  |  |  | hPancreas [17] and Multiple Sclerosis (MS) [110] | - | cell type annotation |
|  |  |  |  |  |  |  | the Adamson Perturb-Seq dataset [19] and the Norman Perturb-Seq dataset [20] | 87 one-gene perturbations in the Adamson Perturb-Seq dataset, and 131 two-gene perturbations and 105 one-gene perturbations in the Norman Perturb-Seq dataset | perturbation prediction |
|  | scGREAT | [111] | Feb 2024 | - | Transformer-based | single-cell transcriptomics | human embryonic stem cells (hESC) (GSE75748) [112] | - | gene regulatory network inference |
|  |  |  |  |  |  |  | human mature hepatocytes (hHEP) (GSE81252) [113, 114] | - |  |
|  |  |  |  |  |  |  | mouse dendritic cells (mDC) (GSE48968) [115] | - |  |
|  |  |  |  |  |  |  | mouse embryonic stem cells (mESC) (GSE98664) [116] | - |  |
|  |  |  |  |  |  |  | mouse hematopoietic stem cells with erythroid-lineage (mHSC-E)  mouse hematopoietic stem cells with granulocyte-monocyte-lineage (mHSC-GM)  mouse hematopoietic stem cells with lymphoid-lineage (mHSC-L) (GSE81682) [117] | - |  |
|  | BioFormers | [118] | bioRxiv posted Dec 2023 | An 8-layer transformer encoder model with 8 self-attention heads per layer and a hidden state dimension of 512 | BERT-based | scRNA-seq | PBMC [15] | 7,982 cells and 3,346 genes | Cell clustering and identification |
|  |  |  |  |  |  |  | PBMC 4k and 8k [119] | 11,990 cells and 2,000 HVGs | Gene expression prediction |
|  |  |  |  |  |  |  | Perturb-seq dataset [19] | 87 single-gene perturbations, with ~100 cells per perturbation and a control set of at least 7,000 unperturbed cells | genetic perturbation prediction and gene network inference |
|  | scPRINT | [120] | April  2025 | 2M to 100M parameters, 4-layer transformer encoder model with 2 self-attention heads per layer | BERT-based | scRNA-seq | three test datasets of kidney, retina, and colon tissues [121-123] | comprising 26 cell types | gene network inference, denoising, batch effect correction, and cell label prediction |
|  |  |  |  |  |  |  | perturb-seq [124] and ChIP-seq [125] | - |  |
|  |  |  |  |  |  |  | 3 test datasets of ciliary body, colon, and retina [122, 126, 127] | - |  |
|  |  |  |  |  |  |  | premalignant neoplasms from human prostate tissues [128] | - |  |
|  | ScRAT | [129] | Feb 2024 | one-layer transformer encoder model with 8 self-attention heads | Transformer-based | scRNA-seq | COMBAT [130] and Haniffa datasets [131] | 835,937 and 528,438 cells | disease diagnosis |
|  |  |  |  |  |  |  | SC4 with COVID samples [132] | 501,943 cells for severity prediction  1,289,496 cells for stage prediction | predict severity and stage |
|  | CancerFoundation | [133] | bioRxiv posted Nov 2024 | 6 transformer layers, 10.8 million parameters | BERT-based | scRNA-seq | glioblastoma dataset [134] | four distinct malignant cell states | batch integration |
|  |  |  |  |  |  |  | CCLE [98] and GDSC [99] | - | Drug response prediction |
|  |  |  |  |  |  | bulk RNA-seq data | TCGA data [135, 136] | 21 cancer types | Survival prediction |
|  | mcBERT | [137] | bioRxiv posted Nov 2024 | 12 blocks, each with 12  attention heads | BERT-based | scRNA-seq | Heart [9, 84, 138-141] | Refer to Table 1 in [137] | Patient-level representation,  disease clustering, phenotypical interpretation/batch effect removal |
|  |  |  |  |  |  |  | Kidney [142-146] |  |  |
|  |  |  |  |  |  |  | PBMC [130, 147, 148] |  |  |
|  |  |  |  |  |  |  | Lung [149] |  |  |
|  | MarsGT | [150] | Jan 2024 | 3 layers and 8 heads | graph transformer | matched scRNA-seq and scATAC-seq data | mouse retina data [151] | 9,383 cells | Rare population inference |
|  |  |  |  |  |  |  | human lymph node from 10x Genomics | 14,566 cells |  |
|  |  |  |  |  |  |  | human melanoma [152] | 8 melanoma patients' PBMCs and 2 healthy donor PBMCs |  |
|  | GLEmLN | [153] | bioRxiv posted Sep 2025 | 3 blocks and 8 heads | graph transformer | scRNA-seq | Immune Human dataset [16] | 33,506 cells | Cell type annotation |
|  |  |  |  |  |  |  | Immune Human dataset [16] and Cancer Infiltrating Myeloid dataset (https://drive.google.com/drive/folders/1VbpApQufZq8efFGakW3y8QDDpY9MBoDS) | - | Cell type annotation, Graph Structure Understanding |
|  | Nicheformer | [154] | Oct 2025 | 12 transformer encoder units with 16 attention heads per layer and a feed-forward network size of 1,024 to generate a 512-dimensional embedding of the pretraining dataset, resulting in altogether 49.3M parameters. | BERT-based | single-cell and spatial transcriptomics data | MERFISH mouse brain [155] | 4.3 million cells across 59 tissue sections | Label prediction (cell type, niche and region labels) |
|  |  |  |  |  |  |  | CosMx human liver [156] | 332,877 healthy cells and 460,441 cancer cells | niche label prediction (healthy data only), niche composition prediction |
|  |  |  |  |  |  |  | CosMx human lung [156] | five different donors (301,611, 89,975, 227,110, 71,304 and 81,236 cells, respectively) | niche composition prediction |
|  |  |  |  |  |  |  | Xenium human lung from 10X Genomics | 295,883 healthy cells and 531,165 cancer cells | neighborhood density prediction |
|  |  |  |  |  |  |  | Xenium human colon from 10X Genomics | 275,822 healthy cells and 587,115 cancer cells |  |
|  | SpaFormer | [157] | arXiv posted  Feb 2023 | 2 layers and 8 heads | Transformer-based | Spatial transcriptomics data | Lung 5 data generated by the CosMX platform [156] | 99,656 cells | spatial transcriptomic imputation |
|  |  |  |  |  |  |  | Kidney 1139 data generated by the CosMX platform [156] | 61,283 cells |  |
|  |  |  |  |  |  |  | Liver normal generated by the CosMX platform [156] | 305,730 cells |  |
|  | Novae | [158] | Dec 2025 | Layers and heads vary among datasets | GAT-based | Spatial transcriptomics data | Mouse brain by Xenium platform( <https://www.10xgenomics.com/datasets/xenium-in-situ-analysis-of-alzheimers-disease-mouse-model-brain-coronal-sections-from-one-hemisphere-over-a-time-course-1-standard>) | 270,984 cells | Spatial domain identification |
|  |  |  |  |  |  |  | Multi-modal data(include ST, Phenocycler) <https://www.10xgenomics.com/datasets/xenium-human-lung-cancer-post-xenium-technote> | 388,175 cells |  |
|  | stFormer | [159] | bioRxiv posted Aug 2025 | 6 transformer blocks | BERT-based | Single-cell spatial transcriptomics data | myocardial Visium section (<https://cellxgene.cziscience.com/collections/8191c283-0816-424b-9b61-c3e1d6258a77>) | 4,992 cells | Batch effect removal, gene function prediction |
|  | SpaGT | [160] | April  2025 | Graph transformer | Graph transformer | Spatial transcriptomics data | DLPFC datasets [161] | 4000 spots per slide | spatial domain identification |
|  |  |  |  |  |  |  | MERFISH dataset [162] | 4,096 cells | spatial domain identification |
|  |  |  |  |  |  |  | TNBC dataset [163] | 1,848 spots | spatial domain identification |
|  |  |  |  |  |  |  | Embryo dataset [164] | 41,786 spots | spatial domain identification |
|  | scGPT-spatial | [165] | bioRxiv posted Feb 2025 | 12 stacked transformer blocks with 8 attention heads, 512 embedding sizes of the pre-trained foundation model, 512 hidden sizes of the fully connected layer | GPT-based | Spatial transcriptomics data | DLPFC datasets [161] | 4000 spots per slides | Spatial clustering |

**References**

1. Yang, F., et al., *scBERT as a large-scale pretrained deep language model for cell type annotation of single-cell RNA-seq data.* Nature Machine Intelligence, 2022. **4**(10): p. 852-866.

2. Franzen, O., L.M. Gan, and J.L.M. Bjorkegren, *PanglaoDB: a web server for exploration of mouse and human single-cell RNA sequencing data.* Database (Oxford), 2019. **2019**.

3. Zheng, G.X., et al., *Massively parallel digital transcriptional profiling of single cells.* Nat Commun, 2017. **8**: p. 14049.

4. Baron, M., et al., *A Single-Cell Transcriptomic Map of the Human and Mouse Pancreas Reveals Inter- and Intra-cell Population Structure.* Cell Syst, 2016. **3**(4): p. 346-360 e4.

5. Muraro, M.J., et al., *A Single-Cell Transcriptome Atlas of the Human Pancreas.* Cell Syst, 2016. **3**(4): p. 385-394 e3.

6. Segerstolpe, A., et al., *Single-Cell Transcriptome Profiling of Human Pancreatic Islets in Health and Type 2 Diabetes.* Cell Metab, 2016. **24**(4): p. 593-607.

7. Xin, Y., et al., *RNA Sequencing of Single Human Islet Cells Reveals Type 2 Diabetes Genes.* Cell Metab, 2016. **24**(4): p. 608-615.

8. MacParland, S.A., et al., *Single cell RNA sequencing of human liver reveals distinct intrahepatic macrophage populations.* Nat Commun, 2018. **9**(1): p. 4383.

9. Litvinukova, M., et al., *Cells of the adult human heart.* Nature, 2020. **588**(7838): p. 466-472.

10. Tucker, N.R., et al., *Transcriptional and Cellular Diversity of the Human Heart.* Circulation, 2020. **142**(5): p. 466-482.

11. Lukassen, S., et al., *SARS-CoV-2 receptor ACE2 and TMPRSS2 are primarily expressed in bronchial transient secretory cells.* EMBO J, 2020. **39**(10): p. e105114.

12. He, S., et al., *Single-cell transcriptome profiling of an adult human cell atlas of 15 major organs.* Genome Biol, 2020. **21**(1): p. 294.

13. Cui, H., et al., *scGPT: toward building a foundation model for single-cell multi-omics using generative AI.* Nat Methods, 2024. **21**(8): p. 1470-1480.

14. (n.d.)., C.Z.I., *CZ CELLxGENE Discover*. 2022, <https://cellxgene.cziscience.com/>.

15. Gayoso, A., et al., *A Python library for probabilistic analysis of single-cell omics data.* Nat Biotechnol, 2022. **40**(2): p. 163-166.

16. Luecken, M.D., et al., *Benchmarking atlas-level data integration in single-cell genomics.* Nat Methods, 2022. **19**(1): p. 41-50.

17. Chen, J., et al., *Transformer for one stop interpretable cell type annotation.* Nat Commun, 2023. **14**(1): p. 223.

18. Lawlor, N., et al., *Single-cell transcriptomes identify human islet cell signatures and reveal cell-type-specific expression changes in type 2 diabetes.* Genome Res, 2017. **27**(2): p. 208-222.

19. Adamson, B., et al., *A Multiplexed Single-Cell CRISPR Screening Platform Enables Systematic Dissection of the Unfolded Protein Response.* Cell, 2016. **167**(7): p. 1867-1882 e21.

20. Norman, T.M., et al., *Exploring genetic interaction manifolds constructed from rich single-cell phenotypes.* Science, 2019. **365**(6455): p. 786-793.

21. Cusanovich, D.A., et al., *Multiplex single cell profiling of chromatin accessibility by combinatorial cellular indexing.* Science, 2015. **348**(6237): p. 910-4.

22. Mimitou, E.P., et al., *Scalable, multimodal profiling of chromatin accessibility, gene expression and protein levels in single cells.* Nat Biotechnol, 2021. **39**(10): p. 1246-1258.

23. Xu, J., et al., *CIForm as a Transformer-based model for cell-type annotation of large-scale single-cell RNA-seq data.* Brief Bioinform, 2023. **24**(4).

24. Sun, Z., et al., *A Bayesian mixture model for clustering droplet-based single-cell transcriptomic data from population studies.* Nat Commun, 2019. **10**(1): p. 1649.

25. Oetjen, K.A., et al., *Human bone marrow assessment by single-cell RNA sequencing, mass cytometry, and flow cytometry.* JCI Insight, 2018. **3**(23).

26. Dahlin, J.S., et al., *A single-cell hematopoietic landscape resolves 8 lineage trajectories and defects in Kit mutant mice.* Blood, 2018. **131**(21): p. e1-e11.

27. Zeisel, A., et al., *Molecular Architecture of the Mouse Nervous System.* Cell, 2018. **174**(4): p. 999-1014 e22.

28. Saunders, A., et al., *Molecular Diversity and Specializations among the Cells of the Adult Mouse Brain.* Cell, 2018. **174**(4): p. 1015-1030 e16.

29. Rosenberg, A.B., et al., *Single-cell profiling of the developing mouse brain and spinal cord with split-pool barcoding.* Science, 2018. **360**(6385): p. 176-182.

30. Tabula Muris, C., et al., *Single-cell transcriptomics of 20 mouse organs creates a Tabula Muris.* Nature, 2018. **562**(7727): p. 367-372.

31. Zhang, L., et al., *Lineage tracking reveals dynamic relationships of T cells in colorectal cancer.* Nature, 2018. **564**(7735): p. 268-272.

32. Tasic, B., et al., *Shared and distinct transcriptomic cell types across neocortical areas.* Nature, 2018. **563**(7729): p. 72-78.

33. Chou, C.H., et al., *Synovial cell cross-talk with cartilage plays a major role in the pathogenesis of osteoarthritis.* Sci Rep, 2020. **10**(1): p. 10868.

34. Alsaigh, T., et al., *Decoding the transcriptome of calcified atherosclerotic plaque at single-cell resolution.* Commun Biol, 2022. **5**(1): p. 1084.

35. Zeisel, A., et al., *Brain structure. Cell types in the mouse cortex and hippocampus revealed by single-cell RNA-seq.* Science, 2015. **347**(6226): p. 1138-42.

36. Bastidas-Ponce, A., et al., *Comprehensive single cell mRNA profiling reveals a detailed roadmap for pancreatic endocrinogenesis.* Development, 2019. **146**(12).

37. Tabula Muris, C., *A single-cell transcriptomic atlas characterizes ageing tissues in the mouse.* Nature, 2020. **583**(7817): p. 590-595.

38. Jiao, L., et al., *scTransSort: Transformers for Intelligent Annotation of Cell Types by Gene Embeddings.* Biomolecules, 2023. **13**(4).

39. Shao, X., et al., *scDeepSort: a pre-trained cell-type annotation method for single-cell transcriptomics using deep learning with a weighted graph neural network.* Nucleic Acids Res, 2021. **49**(21): p. e122.

40. Song, T., et al., *TransCluster: A Cell-Type Identification Method for single-cell RNA-Seq data using deep learning based on transformer.* Front Genet, 2022. **13**: p. 1038919.

41. Han, X., et al., *Construction of a human cell landscape at single-cell level.* Nature, 2020. **581**(7808): p. 303-309.

42. Theodoris, C.V., et al., *Transfer learning enables predictions in network biology.* Nature, 2023. **618**(7965): p. 616-624.

43. Lhoest, Q., et al., *Datasets: A community library for natural language processing.* arXiv preprint arXiv:2109.02846, 2021.

44. Shen, H., et al., *Generative pretraining from large-scale transcriptomes for single-cell deciphering.* iScience, 2023. **26**(5): p. 106536.

45. Regev, A., et al., *The human cell atlas white paper.* arXiv preprint arXiv:1810.05192, 2018.

46. Peng, Y.R., et al., *Molecular Classification and Comparative Taxonomics of Foveal and Peripheral Cells in Primate Retina.* Cell, 2019. **176**(5): p. 1222-1237 e22.

47. Johnson, W.E., C. Li, and A. Rabinovic, *Adjusting batch effects in microarray expression data using empirical Bayes methods.* Biostatistics, 2007. **8**(1): p. 118-27.

48. Huang, T.X. and L. Fu, *The immune landscape of esophageal cancer.* Cancer Commun (Lond), 2019. **39**(1): p. 79.

49. Ma, A., et al., *Single-cell biological network inference using a heterogeneous graph transformer.* Nat Commun, 2023. **14**(1): p. 964.

50. Luecken, M.D., et al. *A sandbox for prediction and integration of DNA, RNA, and proteins in single cells*. in *Thirty-fifth conference on neural information processing systems datasets and benchmarks track (Round 2)*. 2021.

51. Li, G., et al., *A deep generative model for multi-view profiling of single-cell RNA-seq and ATAC-seq data.* Genome Biol, 2022. **23**(1): p. 20.

52. Cao, J., et al., *Joint profiling of chromatin accessibility and gene expression in thousands of single cells.* Science, 2018. **361**(6409): p. 1380-1385.

53. Zhu, C., et al., *An ultra high-throughput method for single-cell joint analysis of open chromatin and transcriptome.* Nat Struct Mol Biol, 2019. **26**(11): p. 1063-1070.

54. Chen, S., B.B. Lake, and K. Zhang, *High-throughput sequencing of the transcriptome and chromatin accessibility in the same cell.* Nat Biotechnol, 2019. **37**(12): p. 1452-1457.

55. Ma, S., et al., *Chromatin Potential Identified by Shared Single-Cell Profiling of RNA and Chromatin.* Cell, 2020. **183**(4): p. 1103-1116 e20.

56. Liu, L., et al., *A pre-trained large generative model for translating single-cell transcriptomes to proteomes.* Nat Biomed Eng, 2025.

57. Cancer Genome Atlas Research, N., *Comprehensive molecular characterization of clear cell renal cell carcinoma.* Nature, 2013. **499**(7456): p. 43-9.

58. Ciriello, G., et al., *Comprehensive Molecular Portraits of Invasive Lobular Breast Cancer.* Cell, 2015. **163**(2): p. 506-19.

59. Fishbein, L., et al., *Comprehensive Molecular Characterization of Pheochromocytoma and Paraganglioma.* Cancer Cell, 2017. **31**(2): p. 181-193.

60. Kahles, A., et al., *Comprehensive Analysis of Alternative Splicing Across Tumors from 8,705 Patients.* Cancer Cell, 2018. **34**(2): p. 211-224 e6.

61. Cao, L., et al., *Proteogenomic characterization of pancreatic ductal adenocarcinoma.* Cell, 2021. **184**(19): p. 5031-5052 e26.

62. Dou, Y., et al., *Proteogenomic Characterization of Endometrial Carcinoma.* Cell, 2020. **180**(4): p. 729-748 e26.

63. Gillette, M.A., et al., *Proteogenomic Characterization Reveals Therapeutic Vulnerabilities in Lung Adenocarcinoma.* Cell, 2020. **182**(1): p. 200-225 e35.

64. Krug, K., et al., *Proteogenomic Landscape of Breast Cancer Tumorigenesis and Targeted Therapy.* Cell, 2020. **183**(5): p. 1436-1456 e31.

65. Petralia, F., et al., *Integrated Proteogenomic Characterization across Major Histological Types of Pediatric Brain Cancer.* Cell, 2020. **183**(7): p. 1962-1985 e31.

66. Satpathy, S., et al., *A proteogenomic portrait of lung squamous cell carcinoma.* Cell, 2021. **184**(16): p. 4348-4371 e40.

67. Wang, L.B., et al., *Proteogenomic and metabolomic characterization of human glioblastoma.* Cancer Cell, 2021. **39**(4): p. 509-528 e20.

68. Encyclopedia, T.C.C.L., et al., *Consistency of drug profiles and predictors in large-scale cancer cell line data.* Nature, 2015. **528**(7580): p. 84.

69. Nusinow, D.P., et al., *Quantitative Proteomics of the Cancer Cell Line Encyclopedia.* Cell, 2020. **180**(2): p. 387-402 e16.

70. Pietzak, E.J., et al., *Genomic Differences Between "Primary" and "Secondary" Muscle-invasive Bladder Cancer as a Basis for Disparate Outcomes to Cisplatin-based Neoadjuvant Chemotherapy.* Eur Urol, 2019. **75**(2): p. 231-239.

71. Hao, Y., et al., *Integrated analysis of multimodal single-cell data.* Cell, 2021. **184**(13): p. 3573-3587 e29.

72. Peterson, V.M., et al., *Multiplexed quantification of proteins and transcripts in single cells.* Nat Biotechnol, 2017. **35**(10): p. 936-939.

73. Stoeckius, M., et al., *Simultaneous epitope and transcriptome measurement in single cells.* Nat Methods, 2017. **14**(9): p. 865-868.

74. Cheng, S., et al., *A pan-cancer single-cell transcriptional atlas of tumor infiltrating myeloid cells.* Cell, 2021. **184**(3): p. 792-809 e23.

75. Hao, M., et al., *Large-scale foundation model on single-cell transcriptomics.* Nat Methods, 2024. **21**(8): p. 1481-1491.

76. Liu, Q., et al., *DeepCDR: a hybrid graph convolutional network for predicting cancer drug response.* Bioinformatics, 2020. **36**(Suppl_2): p. i911-i918.

77. Roohani, Y., K. Huang, and J. Leskovec, *Predicting transcriptional outcomes of novel multigene perturbations with GEARS.* Nat Biotechnol, 2023.

78. Tang, W., et al. *Single-cell multimodal prediction via transformers*. in *Proceedings of the 32nd ACM International Conference on Information and Knowledge Management*. 2023.

79. Yang, X., et al., *GeneCompass: deciphering universal gene regulatory mechanisms with a knowledge-informed cross-species foundation model.* Cell Res, 2024. **34**(12): p. 830-845.

80. Qiao, Y., et al., *Synergistic activation of inflammatory cytokine genes by interferon-gamma-induced chromatin remodeling and toll-like receptor signaling.* Immunity, 2013. **39**(3): p. 454-69.

81. Srivatsan, S.R., et al., *Massively multiplex chemical transcriptomics at single-cell resolution.* Science, 2020. **367**(6473): p. 45-51.

82. Garipler, G., et al., *The BTB transcription factors ZBTB11 and ZFP131 maintain pluripotency by repressing pro-differentiation genes.* Cell Rep, 2022. **38**(11): p. 110524.

83. Bian, H., et al. *scMulan: a multitask generative pre-trained language model for single-cell analysis*. in *International Conference on Research in Computational Molecular Biology*. 2024. Springer.

84. Simonson, B., et al., *Single-nucleus RNA sequencing in ischemic cardiomyopathy reveals common transcriptional profile underlying end-stage heart failure.* Cell Rep, 2023. **42**(2): p. 112086.

85. Suo, C., et al., *Mapping the developing human immune system across organs.* Science, 2022. **376**(6597): p. eabo0510.

86. Lotfollahi, M., et al., *Mapping single-cell data to reference atlases by transfer learning.* Nat Biotechnol, 2022. **40**(1): p. 121-130.

87. Rosen, Y., et al., *Universal cell embeddings: A foundation model for cell biology.* bioRxiv, 2023: p. 2023.11. 28.568918.

88. Dominguez Conde, C., et al., *Cross-tissue immune cell analysis reveals tissue-specific features in humans.* Science, 2022. **376**(6594): p. eabl5197.

89. Speranza, E., et al., *Single-cell RNA sequencing reveals SARS-CoV-2 infection dynamics in lungs of African green monkeys.* Sci Transl Med, 2021. **13**(578).

90. Hilton, H.G., et al., *Single-cell transcriptomics of the naked mole-rat reveals unexpected features of mammalian immunity.* PLoS Biol, 2019. **17**(11): p. e3000528.

91. Yamagata, M., W. Yan, and J.R. Sanes, *A cell atlas of the chick retina based on single-cell transcriptomics.* Elife, 2021. **10**.

92. Orozco, L.D., et al., *Integration of eQTL and a Single-Cell Atlas in the Human Eye Identifies Causal Genes for Age-Related Macular Degeneration.* Cell Rep, 2020. **30**(4): p. 1246-1259 e6.

93. Zhao, S., J. Zhang, and Z. Nie, *Large-scale cell representation learning via divide-and-conquer contrastive learning.* arXiv preprint arXiv:2306.04371, 2023.

94. Aissa, A.F., et al., *Single-cell transcriptional changes associated with drug tolerance and response to combination therapies in cancer.* Nat Commun, 2021. **12**(1): p. 1628.

95. Sharma, A., et al., *Longitudinal single-cell RNA sequencing of patient-derived primary cells reveals drug-induced infidelity in stem cell hierarchy.* Nat Commun, 2018. **9**(1): p. 4931.

96. Ravasio, A., et al., *Single-cell analysis of EphA clustering phenotypes to probe cancer cell heterogeneity.* Commun Biol, 2020. **3**(1): p. 429.

97. Suphavilai, C., et al., *Predicting heterogeneity in clone-specific therapeutic vulnerabilities using single-cell transcriptomic signatures.* Genome Med, 2021. **13**(1): p. 189.

98. Barretina, J., et al., *The Cancer Cell Line Encyclopedia enables predictive modelling of anticancer drug sensitivity.* Nature, 2012. **483**(7391): p. 603-7.

99. Iorio, F., et al., *A Landscape of Pharmacogenomic Interactions in Cancer.* Cell, 2016. **166**(3): p. 740-754.

100. Xiong, L., T. Chen, and M. Kellis. *scCLIP: Multi-modal Single-cell Contrastive Learning Integration Pre-training*. in *NeurIPS 2023 AI for Science Workshop*.

101. Cao, J., et al., *A human cell atlas of fetal gene expression.* Science, 2020. **370**(6518).

102. Domcke, S., et al., *A human cell atlas of fetal chromatin accessibility.* Science, 2020. **370**(6518).

103. Anderson, A.G., et al., *Single nucleus multiomics identifies ZEB1 and MAFB as candidate regulators of Alzheimer's disease-specific cis-regulatory elements.* Cell Genom, 2023. **3**(3): p. 100263.

104. Shen, H., et al., *A universal approach for integrating super large-scale single-cell transcriptomes by exploring gene rankings.* Brief Bioinform, 2022. **23**(2).

105. Kang, H.M., et al., *Multiplexed droplet single-cell RNA-sequencing using natural genetic variation.* Nat Biotechnol, 2018. **36**(1): p. 89-94.

106. Guo, X., et al., *Global characterization of T cells in non-small-cell lung cancer by single-cell sequencing.* Nat Med, 2018. **24**(7): p. 978-985.

107. Zheng, C., et al., *Landscape of Infiltrating T Cells in Liver Cancer Revealed by Single-Cell Sequencing.* Cell, 2017. **169**(7): p. 1342-1356 e16.

108. Wen, H., et al., *CellPLM: pre-training of cell language model beyond single cells.* bioRxiv, 2023: p. 2023.10. 03.560734.

109. Li, Y., et al., *Single-Cell Transcriptome Analysis Reveals Dynamic Cell Populations and Differential Gene Expression Patterns in Control and Aneurysmal Human Aortic Tissue.* Circulation, 2020. **142**(14): p. 1374-1388.

110. Schirmer, L., et al., *Neuronal vulnerability and multilineage diversity in multiple sclerosis.* Nature, 2019. **573**(7772): p. 75-82.

111. Wang, Y., et al., *scGREAT: Transformer-based deep-language model for gene regulatory network inference from single-cell transcriptomics.* iScience, 2024. **27**(4): p. 109352.

112. Chu, L.F., et al., *Single-cell RNA-seq reveals novel regulators of human embryonic stem cell differentiation to definitive endoderm.* Genome Biol, 2016. **17**(1): p. 173.

113. Mora-Bermudez, F., et al., *Differences and similarities between human and chimpanzee neural progenitors during cerebral cortex development.* Elife, 2016. **5**.

114. Camp, J.G., et al., *Multilineage communication regulates human liver bud development from pluripotency.* Nature, 2017. **546**(7659): p. 533-538.

115. Shalek, A.K., et al., *Single-cell RNA-seq reveals dynamic paracrine control of cellular variation.* Nature, 2014. **510**(7505): p. 363-9.

116. Hayashi, T., et al., *Single-cell full-length total RNA sequencing uncovers dynamics of recursive splicing and enhancer RNAs.* Nat Commun, 2018. **9**(1): p. 619.

117. Nestorowa, S., et al., *A single-cell resolution map of mouse hematopoietic stem and progenitor cell differentiation.* Blood, 2016. **128**(8): p. e20-31.

118. Amara-Belgadi, S., et al., *BIOFORMERS: A SCALABLE FRAMEWORK FOR EXPLORING BIOSTATES USING TRANSFORMERS.* bioRxiv, 2023: p. 2023.11. 29.569320.

119. Zheng, G.X., et al., *Massively parallel digital transcriptional profiling of single cells.* Nature communications, 2017. **8**(1): p. 14049.

120. Kalfon, J., et al., *scPRINT: pre-training on 50 million cells allows robust gene network predictions.* Nat Commun, 2025. **16**(1): p. 3607.

121. Kong, L., et al., *The landscape of immune dysregulation in Crohn's disease revealed through single-cell transcriptomic profiling in the ileum and colon.* Immunity, 2023. **56**(2): p. 444-458 e5.

122. Wang, S.K., et al., *Single-cell multiome of the human retina and deep learning nominate causal variants in complex eye diseases.* Cell Genom, 2022. **2**(8).

123. Marshall, J.L., et al., *High-resolution Slide-seqV2 spatial transcriptomics enables discovery of disease-specific cell neighborhoods and pathways.* iScience, 2022. **25**(4): p. 104097.

124. Dixit, A., et al., *Perturb-Seq: Dissecting Molecular Circuits with Scalable Single-Cell RNA Profiling of Pooled Genetic Screens.* Cell, 2016. **167**(7): p. 1853-1866 e17.

125. Park, P.J., *ChIP-seq: advantages and challenges of a maturing technology.* Nat Rev Genet, 2009. **10**(10): p. 669-80.

126. Burclaff, J., et al., *A Proximal-to-Distal Survey of Healthy Adult Human Small Intestine and Colon Epithelium by Single-Cell Transcriptomics.* Cell Mol Gastroenterol Hepatol, 2022. **13**(5): p. 1554-1589.

127. van Zyl, T., et al., *Cell atlas of the human ocular anterior segment: Tissue-specific and shared cell types.* Proc Natl Acad Sci U S A, 2022. **119**(29): p. e2200914119.

128. Joseph, D.B., et al., *Single-cell analysis of mouse and human prostate reveals novel fibroblasts with specialized distribution and microenvironment interactions.* J Pathol, 2021. **255**(2): p. 141-154.

129. Mao, Y., et al., *Phenotype prediction from single-cell RNA-seq data using attention-based neural networks.* Bioinformatics, 2024. **40**(2).

130. julian.knight@well.ox.ac.uk, C.O.-M.-o.B.A.C.E.a. and C.O.-M.-o.B.A. Consortium, *A blood atlas of COVID-19 defines hallmarks of disease severity and specificity.* Cell, 2022. **185**(5): p. 916-938 e58.

131. Stephenson, E., et al., *Single-cell multi-omics analysis of the immune response in COVID-19.* Nat Med, 2021. **27**(5): p. 904-916.

132. Ren, X., et al., *COVID-19 immune features revealed by a large-scale single-cell transcriptome atlas.* Cell, 2021. **184**(7): p. 1895-1913 e19.

133. Theus, A., et al., *CancerFoundation: A single-cell RNA sequencing foundation model to decipher drug resistance in cancer.* bioRxiv, 2024: p. 2024.11. 01.621087.

134. Neftel, C., et al., *An Integrative Model of Cellular States, Plasticity, and Genetics for Glioblastoma.* Cell, 2019. **178**(4): p. 835-849 e21.

135. Wissel, D., et al., *Survboard: standardised benchmarking for multi-omics cancer survival models.* bioRxiv, 2022: p. 2022.11. 18.517043.

136. Cancer Genome Atlas Research, N., et al., *The Cancer Genome Atlas Pan-Cancer analysis project.* Nat Genet, 2013. **45**(10): p. 1113-20.

137. Querfurth, B.v., et al., *mcBERT: Patient-Level Single-cell Transcriptomics Data Representation.* bioRxiv, 2024: p. 2024.11. 04.621897.

138. Chaffin, M., et al., *Single-nucleus profiling of human dilated and hypertrophic cardiomyopathy.* Nature, 2022. **608**(7921): p. 174-180.

139. Koenig, A.L., et al., *Single-cell transcriptomics reveals cell-type-specific diversification in human heart failure.* Nat Cardiovasc Res, 2022. **1**(3): p. 263-280.

140. Reichart, D., et al., *Pathogenic variants damage cell composition and single cell transcription in cardiomyopathies.* Science, 2022. **377**(6606): p. eabo1984.

141. Kuppe, C., et al., *Spatial multi-omic map of human myocardial infarction.* Nature, 2022. **608**(7924): p. 766-777.

142. Lake, B.B., et al., *An atlas of healthy and injured cell states and niches in the human kidney.* Nature, 2023. **619**(7970): p. 585-594.

143. Kuppe, C., et al., *Decoding myofibroblast origins in human kidney fibrosis.* Nature, 2021. **589**(7841): p. 281-286.

144. Muto, Y., et al., *Defining cellular complexity in human autosomal dominant polycystic kidney disease by multimodal single cell analysis.* Nat Commun, 2022. **13**(1): p. 6497.

145. Wilson, P.C., et al., *Multimodal single cell sequencing implicates chromatin accessibility and genetic background in diabetic kidney disease progression.* Nat Commun, 2022. **13**(1): p. 5253.

146. Muto, Y., et al., *Single cell transcriptional and chromatin accessibility profiling redefine cellular heterogeneity in the adult human kidney.* Nat Commun, 2021. **12**(1): p. 2190.

147. Perez, R.K., et al., *Single-cell RNA-seq reveals cell type-specific molecular and genetic associations to lupus.* Science, 2022. **376**(6589): p. eabf1970.

148. Yoshida, M., et al., *Local and systemic responses to SARS-CoV-2 infection in children and adults.* Nature, 2022. **602**(7896): p. 321-327.

149. Sikkema, L., et al., *An integrated cell atlas of the lung in health and disease.* Nat Med, 2023. **29**(6): p. 1563-1577.

150. Wang, X., et al., *MarsGT: Multi-omics analysis for rare population inference using single-cell graph transformer.* bioRxiv, 2023.

151. Dou, J., et al., *Bi-order multimodal integration of single-cell data.* Genome Biol, 2022. **23**(1): p. 112.

152. Boukhaled, G.M., et al., *Pre-encoded responsiveness to type I interferon in the peripheral immune system defines outcome of PD1 blockade therapy.* Nat Immunol, 2022. **23**(8): p. 1273-1283.

153. Zhang, M., et al., *GREmLN: A Cellular Regulatory Network-Aware Transcriptomics Foundation Model.* bioRxiv, 2025: p. 2025.07. 03.663009.

154. Tejada-Lapuerta, A., et al., *Nicheformer: a foundation model for single-cell and spatial omics.* Nat Methods, 2025. **22**(12): p. 2525-2538.

155. Yao, Z., et al., *A high-resolution transcriptomic and spatial atlas of cell types in the whole mouse brain.* Nature, 2023. **624**(7991): p. 317-332.

156. He, S., et al., *High-plex multiomic analysis in FFPE at subcellular level by spatial molecular imaging. bioRxiv 467020.* 2021.

157. Wen, H., et al., *Single cells are spatial tokens: Transformers for spatial transcriptomic data imputation.* arXiv preprint arXiv:2302.03038, 2023.

158. Blampey, Q., et al., *Novae: a graph-based foundation model for spatial transcriptomics data.* Nat Methods, 2025. **22**(12): p. 2539-2550.

159. Cao, S., et al., *stformer: a foundation model for spatial transcriptomics. bioRxiv.* 2024.

160. Bao, X., et al., *Spatially informed graph transformers for spatially resolved transcriptomics.* Commun Biol, 2025. **8**(1): p. 574.

161. Maynard, K.R., et al., *Transcriptome-scale spatial gene expression in the human dorsolateral prefrontal cortex.* Nat Neurosci, 2021. **24**(3): p. 425-436.

162. Codeluppi, S., et al., *Spatial organization of the somatosensory cortex revealed by osmFISH.* Nat Methods, 2018. **15**(11): p. 932-935.

163. Coutant, A., et al., *Spatial Transcriptomics Reveal Pitfalls and Opportunities for the Detection of Rare High-Plasticity Breast Cancer Subtypes.* Lab Invest, 2023. **103**(12): p. 100258.

164. Sunkin, S.M., et al., *Allen Brain Atlas: an integrated spatio-temporal portal for exploring the central nervous system.* Nucleic Acids Res, 2013. **41**(Database issue): p. D996-D1008.

165. Wang, C., et al., *scGPT-spatial: Continual pretraining of single-cell foundation model for spatial transcriptomics.* bioRxiv, 2025: p. 2025.02. 05.636714.
