# Supplementary material for: Advancing bioinformatics with language models: components, applications, and perspectives
Source: Brief Bioinform. 2026 Jul 10;27(4):bbag367. doi: 10.1093/bib/bbag367 (PMC13354062; doi:10.1093/bib/bbag367)
Supplement: Supplementary_material_bbag367 [file supplementary_material_bbag367.zip › Supplementary Table 5.docx]

**Supplementary Table 5. Benchmarking of existing language models in bioinformatics.**

| **Application area** | **Input data** | **Biological tasks** | | **Benchmarking datasets** | **Performance rank**  **(descending order)** | **References** |
| --- | --- | --- | --- | --- | --- | --- |
| Genomics | DNA sequence | Genome-wide variant effects prediction | | GTEx fine-mapped eQTLs (PIP >0.9 vs <0.01); ClinVar / OMIM pathogenic variants vs common gnomAD variants; Cross-task benchmark suites | Alignment-based / conservation-aware models (GPN-MSA, CADD) > short-context DNA LLMs (DNABERT-2) > long-context DNA LLMs (Nucleotide Transformer, HyenaDNA) | [1-3] |
|  |  | DNA cis-regulatory regions prediction | | ENCODE SCREEN v3 promoter/enhancer annotations; Regulatory genomics benchmarks | Fine-tuned DNA LLMs ≈ classical supervised baselines (e.g., DeepSEA) > frozen DNA LLM embeddings | [1-3] |
|  |  | DNA–protein interaction prediction | | ChIP-seq–derived TF binding benchmarks; Cross-task regulatory benchmarks | Fine-tuned DNA LLMs ≈ task-specific supervised models > frozen embeddings | [2, 3] |
|  |  | DNA methylation prediction | | CpG methylation and modification benchmarks; Cross-task benchmark suites | Short-context, base-pair–resolution DNA LLMs (DNABERT-2) > long-context DNA LLMs | [3, 4] |
|  |  | DNA level splice site identification | | Splice-site prediction benchmarks (donor/acceptor classification); Cross-task benchmark suites | Short-context, base-pair–resolution DNA LLMs (DNABERT-2) > long-context DNA LLMs | [3, 4] |
| Transcriptomics | RNA sequence | RNA secondary structure prediction | | ArchiveII; bpRNA; RNAStrAlign (base-pair–level structure recovery) | RNA-FM ≈ RNA-MSM ≈ RNABERT ≈ UNI-RNA > classical folding & CNN/RNN baselines | [5-7] |
|  |  | RNA splicing prediction | | Human splice junction datasets; donor/acceptor classification; alternative splicing events | SpliceBERT ≈ UNI-RNA > CNN/RNN baselines | [5, 6, 8] |
|  |  | lncRNA identification & coding potential prediction | | Cross-species lncRNA datasets; Ribo-seq–validated sORF datasets | LncCat, LSCPP-BERT > ORF-based & feature-engineered baselines | [5, 6] |
|  |  | RNA–protein interaction prediction (RBP binding) | | eCLIP-seq benchmarks across ~150 RBPs | BERT-RBP > sequence-based DL baselines | [5, 6, 8] |
|  |  | RNA–RNA interaction prediction | | miRNA–mRNA and lncRNA–RNA interaction benchmarks (experimentally validated pairs) | RNAErnie-based models > hybrid DL baselines | [5, 8] |
|  |  | RNA modification site prediction | | m7G datasets; Nm (2′-O-methylation) benchmarks | BERT-m7G, Bert2Ome > traditional ML baselines | [5, 6] |
|  |  | Protein expression & mRNA degradation prediction | | Large-scale mRNA expression datasets; mRNA stability / half-life benchmarks | CodonBERT > regression & k-mer baselines | [5, 8] |
|  |  | UTR-based translation efficiency & mRNA localization | | Ribosome loading benchmarks; subcellular localization datasets | UTR-LM ≈ RNA-FM ≈ UNI-RNA > CNN baselines | [5, 6, 8] |
| Proteomics | Protein sequences, MSAs, Gene ontology annotations, Triplets of protein-relation-attribute, Protein property descriptions, cDNA, sequences | Secondary structure and contact prediction | Unsupervised contact prediction | trRosetta test set | MSA Transformer > ESM-1b > ProTrans-T5 > ProtBERT-BFD > TAPE | [9] |
|  |  |  | Supervised contact prediction | CASP14, CASP15, CAMEO test set | ESM3 98B > ESM3 7B > ESM3 1.4B > ESM2 3B | [10] |
|  |  |  | Secondary structure prediction (8-state, Q8) | CB513 | ProtT5-XL-U50 > ProtT5-XXL-U50 > ProtT5-XL-BFD > ESM-1b > ProtT5-XXL-BFD > ProtBert-BFD > ProtBert > ProtAlbert > ProtElectra > ProtXLNet > ProtTXL > ProtTXL-BFD | [11] |
|  |  |  | Secondary Structure Prediction | CB513 | SPRoBERTa-10k (UniRef50) > SPRoBERTa-30k (UniRef50) > RoBERTa Base (UniRef50) > SPRoBERTa-50k (Pfam) > SPRoBERTa-30k (Pfam) > SPRoBERTa-10k (Pfam) > RoBERTa Base (Pfam) > ProteinBERT > TAPE | [12] |
|  |  |  | Single-sequence structure prediction | CASP14, CASP15, CAMEO | ESM3 98B > ESM3 7B > ESM3 1.4B Overtrained > ESM3 1.4B Open > ESM3 1.4B | [10] |
|  |  | Protein sequence generation | Tertiary coordination/motif scaffolding benchmark（prompted generation） | Held-out 46 ligand-binding motifs | ESM3 98B Finetuned (With CoT) > ESM3 98B Finetuned (Without CoT) > ESM3 7B Finetuned (With CoT) > ESM3 7B Finetuned (Without CoT) > ESM3 1.4B Finetuned (With CoT) > ESM3 1.4B Finetuned (Without CoT) | [10] |
|  |  | Protein function prediction | Protein function prediction | UniRef proteins with InterProScan function annotations | ESM3 98B > ESM3 7B > ESM3 1.4B | [10] |
|  |  |  | Subcellular localization prediction | SaprotHub | ProTrek > ProtST-ESM-2 > ESM-2 > ProtST-ESM-1b > ESM-3 | [13] |
|  |  |  | Enzyme Commission (EC) numbers prediction | EC dataset from DeepFRI | PromptProtein > ESM-1b > ProtBERT-BFD > LM-GVP | [14] |
|  |  |  | Gene Ontology term prediction-Biological Process (GO-BP) | DeepFRI benchmarks | ProtST-ESM-2 > ESM-2 > ESM-1b > ProtST-ESM-1b > ProtST-ProtBert > OntoProtein > ProtBert | [15] |
|  |  |  | subcellular localization prediction | SwissProt localization dataset | CaLM > other similarly sized pLMs (ESM/ProtTrans), and is competitive with larger pLMs | [16] |
|  |  | Evolution and mutation prediction | Zero-shot mutational effect / fitness prediction | ProteinGym: 217 DMS datasets (28 viral + 189 non-viral) | ESM3 1.4B > ESM2 | [10] |
|  |  |  | Fluorescence (variant effect) prediction | SaprotHub | ProTrek > ProtST-ESM-2 > ESM-3 > ESM-2 > ProtST-ESM-1b | [13] |
|  |  |  | Remote homology detection | SCOP/SCOPe remote homology benchmark | TAPE > SeqVec > ESM-1b > UniRep | [17] |
|  |  |  | Remote homologous protein search | SCOPe40-test all-versus-all search test | PLMSearch > SS-predictor | [18] |
|  |  | Biophysical properties prediction | Thermostability prediction | SaprotHub | ESM-3 > ProTrek > ProtST-ESM-1b > ESM-2 > ProtST-ESM-2 | [13] |
|  |  |  | Stability landscape prediction | STABILITY dataset from TAPE | PromptProtein > ProtBERT-BFD > ESM-1v > ESM-1b | [14] |
|  |  | Protein-protein interaction and binding affinity prediction | Predict binding affinity | SKEMPI dataset | ESM-1b > KeAP > ProtBert > OntoProtein | [19] |
|  |  | Antigen-Receptor binding prediction | Antibody binding affinity prediction | OAS database | AntiFormer > AntiBERTa > AntiBERTy | [20] |
|  |  |  | TCR–antigen-binding specificity prediction | 10× PBMC-TCR dataset, COVID19-TCR dataset | SC-AIR-BERT > TCR-BERT | [21] |
|  |  |  | MHC class II peptide binding affinity prediction | binding dataset curated by Jensen et al. | BERTMHC > NetMHCIIpan3.2 | [22] |
|  |  | Antigen-Antibody binding prediction | Antigen binding capacity prediction (binary) | HER2–trastuzumab CDR-H3 variant dataset | S²ALM > EATLM > MSA-1b > AbLang-H = AntiBERTa > ESM-1b = AbLang-L | [23] |
|  |  |  | Antigen–antibody binding affinity prediction (regression) | BioMap | S²ALM > AntiBERTa2 > ESM-F > Vanilla BERT | [23] |
|  |  |  | Antibody paratope prediction | Two paratope datasets from Leem et al, and Wang et al. | S²ALM > (EATLM = MSA-1b) > ESM-1b > AbLang-L > AntiBERTa > AbLang-H | [23] |
| Drug discovery | Molecular SMILES, Molecular graphs, Molecular fingerprints and protein sequences, Molecular SMILES and protein sequences | Predicting Molecular Properties | | BACE, Clearance, Delaney, Lipophilicity, BBBP, ClinTox, HIV, Delaney, and Tox21 datasets | ChemBERTa-2 > ChemBERTa-1 | [24] |
|  |  |  |  | BACE, HIV, MUV, Avg.Bio, Tox21, ToxCast, Avg.Tox, BBBP, CYP450 | MolecularGPT > LLaMA2 > Galactica | [25] |
|  |  | Predicting Drug-Target Interaction | | KIBA, DTI-MLCD | TransDTI > ProtAlbert > ProtBert-BFD > ProtBert > ESM-1b > ESM-1v > ESM-1 | [26] |
|  |  |  |  | Clinical Trial Records, DrugBank, ZINC Molecule Database, ICD-10 Coding System | DrugCLIP > BPMF > DeepDDI | [27] |
|  |  |  |  | GPCR, NR | DrugReAlign > medllama3-v20 > GPT-4 > GPT-3.5 | [28] |
|  |  | Predicting Synergistic Effects | | DrugCombDB, | SynerGPT > MAML-DeepDDS > DeepDDS > SciBERT | [29] |
| Single-cell analysis | scRNA-seq data | Cell clustering | | Tabula Sapiens v1 and v2 (35549404) | UCE > Geneformer >scGPT | [30] |
|  |  |  |  | dataset from Li et al. (2020) | CellPLM > scGPT > Geneformer | [31] |
|  |  | Cell type annotation | | human multiple sclerosis (hMS) dataset | GeneCompass > scGPT > Geneformer | [32] |
|  |  |  |  | hMS, hLung, and hLiver datasets, and mBrain, mLung, and mPancreas datasets | GeneCompass > Geneformer > TOSICA |  |
|  |  |  |  | myeloid, MS and human pancreas datasets | scGPT > scBERT > TOSICA | [33] |
|  |  |  |  | AHCA_BoneMarrow | scMulan > Geneformer > scGPT | [34] |
|  |  |  |  | Simonson2023, and Su2022 datasets | scMulan > scGPT > Geneformer |  |
|  |  |  |  | hPancreas (Chen et al., 2023) and Multiple Sclerosis (MS) (Schirmer et al., 2019) datasets | CellPLM > scGPT > scBERT | [31] |
|  |  |  |  | Zheng68K and Baron datasets | CellLM > scBERT | [35] |
|  |  |  |  | Human Immune Cells | GLEmLN > scGPT > scFoundation > Geneformer | [36] |
|  |  | Batch effect removal | | human pancreas and lung datasets | scPRINT > Geneformer >scGPT | [35] |
|  |  |  |  | glioblastoma dataset (Neftel et al., 2019) | scGPT > CancerFoundation | [37] |
|  |  |  |  | Lung dataset | scGPT_FT > scMulan_zeroshot > scGPT_zeroshot > Geneformer | [34] |
|  |  |  |  | COVID-19 dataset | scMulan_zeroshot > scGPT_FT > scGPT_zeroshot > Geneformer |  |
|  |  |  |  | dataset from Li et al. (2020) | CellPLM > scGPT and Geneformer | [31] |
|  |  | Drug response/sensitivity prediction | | Cancer Cell Line Encyclopedia (CCLE) (Barretina et al., 2012) and the Genomics of Cancer Drug Sensitivity (GDSC) (Iorio et al., 2016) databases | CancerFoundation > scFoundation > DeepCDR | [37] |
|  |  |  |  | GSE149383 and GSE117872 | CellLM > scBERT | [35] |
|  |  | Gene regulatory network inference | | three randomly selected test datasets of kidney, retina, and colon tissues comprising 26 cell types57,58,59 | scPRINT > scGPT > Geneformer v2 | [35] |
|  |  |  |  | Immune Human dataset | GeneCompass > scGPT > Geneformer | [32] |
|  |  |  |  | Immune Human dataset and Cancer Infiltrating Myeloid dataset | GLEmLN > scFoundation > Geneformer > scGPT | [36] |
|  |  | Gene expression prediction/Imputation/Denoising | | Norman dataset | GeneCompass > scGPT > Geneformer | [32] |
|  |  |  |  | PBMC 5K and Jurkat datasets | CellPLM > scGPT | [31] |
|  |  | Drug dose-response prediction | | Dataset from Srivatsan et al | GeneCompass = Geneformer > scGPT | [32] |
|  |  | Gene dosage sensitivity prediction | | 10,000 random single-cell transcriptomes | GeneCompass > Geneformer |  |

**References**

1. Marroquin, E.M., et al., *The Human Genomics Long-Range Benchmark: Advancing DNA Language Models.* 2025.

2. Tang, Z., et al., *Evaluating the representational power of pre-trained DNA language models for regulatory genomics.* Genome Biology, 2025. **26**(1): p. 203.

3. Feng, H., et al., *Benchmarking DNA foundation models for genomic and genetic tasks.* Nature communications, 2025. **16**(1): p. 10780.

4. Cherednichenko, O., A. Herbert, and M. Poptsova, *Benchmarking DNA large language models on quadruplexes.* Computational and Structural Biotechnology Journal, 2025. **27**: p. 992-1000.

5. Wang, H., et al., *A Comparative Review of RNA Language Models.* arXiv preprint arXiv:2505.09087, 2025.

6. Wang, H., et al., *RNAscope: Benchmarking RNA Language Models for RNA Sequence Understanding.* 2025.

7. Zablocki, L.I., et al., *Comprehensive benchmarking of large language models for RNA secondary structure prediction.* Briefings in Bioinformatics, 2025. **26**(2): p. bbaf137.

8. Ren, Y., et al., *Beacon: Benchmark for comprehensive rna tasks and language models.* Advances in Neural Information Processing Systems, 2024. **37**: p. 92891-92921.

9. Rao, R.M., et al. *MSA transformer*. in *International Conference on Machine Learning*. 2021.

10. Hayes, T., et al., *Simulating 500 million years of evolution with a language model.* Science, 2025. **387**(6736): p. 850-858.

11. Elnaggar, A., et al., *ProtTrans: Towards Cracking the Language of Lifes Code Through Self-Supervised Deep Learning and High Performance Computing.* IEEE Transactions on Pattern Analysis and Machine Intelligence, 2021: p. 1-1.

12. Wu, L., et al., *SPRoBERTa: protein embedding learning with local fragment modeling.* Briefings in Bioinformatics, 2022. **23**(6): p. bbac401.

13. Su, J., et al., *A trimodal protein language model enables advanced protein searches.* Nature Biotechnology, 2025: p. 1-7.

14. Wang, Z., et al. *Multi-level Protein Structure Pre-training via Prompt Learning*. in *The Eleventh International Conference on Learning Representations*. 2022.

15. Xu, M., et al. *Protst: Multi-modality learning of protein sequences and biomedical texts*. in *International Conference on Machine Learning*. 2023. PMLR.

16. Outeiral, C. and C.M. Deane, *Codon language embeddings provide strong signals for use in protein engineering.* Nature Machine Intelligence, 2024. **6**(2): p. 170-179.

17. Rives, A., et al., *Biological structure and function emerge from scaling unsupervised learning to 250 million protein sequences.* Proceedings of the National Academy of Sciences, 2021. **118**(15): p. e2016239118.

18. Liu, W., et al., *PLMSearch: Protein language model powers accurate and fast sequence search for remote homology.* Nature communications, 2024. **15**(1): p. 2775.

19. Zhou, H.-Y., et al., *Protein Representation Learning via Knowledge Enhanced Primary Structure Modeling.* bioRxiv, 2023: p. 2023-01.

20. Wang, Q., et al., *AntiFormer: graph enhanced large language model for binding affinity prediction.* Briefings in Bioinformatics, 2024. **25**(5).

21. Zhao, Y., et al., *SC-AIR-BERT: a pre-trained single-cell model for predicting the antigen-binding specificity of the adaptive immune receptor.* Brief Bioinform, 2023. **24**(4).

22. Cheng, J., et al., *BERTMHC: improved MHC–peptide class II interaction prediction with transformer and multiple instance learning.* Bioinformatics, 2021. **37**(22): p. 4172-4179.

23. Yin, M., et al., *S2alm: Sequence-structure pre-trained large language model for comprehensive antibody representation learning.* Research, 2025. **8**: p. 0721.

24. *ChemBERTa-2: Towards Chemical Foundation Models.* 2022.

25. *MolecularGPT: Open Large Language Model (LLM).* 2024.

26. Kalakoti, Y., S. Yadav, and D. Sundar, *TransDTI: Transformer-Based Language Models for Estimating DTIs and Building a Drug Recommendation Workflow.* ACS Omega, 2022. **7**(3): p. 2706-2717.

27. Fu, T., et al., *DrugCLIP: Contrastive Drug-Disease Interaction For Drug*, in *Proceedings of the 10th ACM International Conference on Bioinformatics, Computational Biology and Health Informatics*. 2019. p. 542-542.

28. Wei, J., et al., *DrugReAlign: a multisource prompt framework for drug repurposing based on large language models.* BMC Biol, 2024. **22**(1): p. 226.

29. *SynerGPT: In-Context Learning for Personalized Drug Synergy Prediction and Drug Design.*

30. Yang, F., et al., *scBERT as a large-scale pretrained deep language model for cell type annotation of single-cell RNA-seq data.* Nature Machine Intelligence, 2022. **4**(10): p. 852-866.

31. Wen, H., et al., *CellPLM: pre-training of cell language model beyond single cells.* bioRxiv, 2023: p. 2023.10. 03.560734.

32. Yang, X., et al., *GeneCompass: deciphering universal gene regulatory mechanisms with a knowledge-informed cross-species foundation model.* Cell Res, 2024. **34**(12): p. 830-845.

33. Cui, H., et al., *scGPT: toward building a foundation model for single-cell multi-omics using generative AI.* Nat Methods, 2024. **21**(8): p. 1470-1480.

34. Bian, H., et al. *scMulan: a multitask generative pre-trained language model for single-cell analysis*. in *International Conference on Research in Computational Molecular Biology*. 2024. Springer.

35. Zhao, S., J. Zhang, and Z. Nie, *Large-scale cell representation learning via divide-and-conquer contrastive learning.* arXiv preprint arXiv:2306.04371, 2023.

36. Zhang, M., et al., *GREmLN: A Cellular Regulatory Network-Aware Transcriptomics Foundation Model.* bioRxiv, 2025: p. 2025.07. 03.663009.

37. Theus, A., et al., *CancerFoundation: A single-cell RNA sequencing foundation model to decipher drug resistance in cancer.* bioRxiv, 2024: p. 2024.11. 01.621087.
